# The Potential Tumor-Suppressor DHRS7 Inversely Correlates with EGFR Expression in Prostate Cancer Cells and Tumor Samples

**DOI:** 10.3390/cancers14133074

**Published:** 2022-06-23

**Authors:** Simon Stücheli, Selene Araya, Caner Ercan, Seraina O. Moser, John Gallon, Paul Jenö, Salvatore Piscuoglio, Luigi Terracciano, Alex Odermatt

**Affiliations:** 1 Division of Molecular and Systems Toxicology, Department of Pharmaceutical Sciences, University of Basel, 4056 Basel, Switzerland; simon.stuecheli@unibas.ch (S.S.); selene.araya@lonza.com (S.A.); serainaolivia.moser@unibas.ch (S.O.M.); 2 Institute of Medical Genetics and Pathology, University Hospital Basel, 4031 Basel, Switzerland; caner.ercan@usb.ch (C.E.); s.piscuoglio@unibas.ch (S.P.); luigi.terracciano@unibas.ch (L.T.); 3 Visceral Surgery and Precision Medicine Research Laboratory, Department of Biomedicine, University of Basel, 4031 Basel, Switzerland; john.gallon@unibas.ch; 4 Proteomics Core Facility, Biozentrum, University of Basel, 4056 Basel, Switzerland; p.jenoe@bluewin.ch

**Keywords:** DHRS7, EGFR, STAT3, prostate cancer, survival, phosphorylation, androgen, metabolism

## Abstract

**Simple Summary:**

Prostate cancer is one of the most common malignancies in men. Current therapies are initially effective but resistance often develops, leading to tumor recurrence and death. Further research on new players, mechanisms involved in prostate cancer, and therapy resistance is needed. We studied the role of DHRS7, a potential tumor suppressor with currently unknown physiological function, in prostate cancer cells using proteome and gene expression analyses. Despite the fact that DHRS7 can inactivate 5α-dihydrotestosterone, its effect on prostate cancer cells seems to be unrelated to androgen metabolism. When comparing three widely studied prostate cancer cell lines, we observed a negative correlation between DHRS7 and EGFR expression. *DHRS7* knockdown enhanced EGFR expression, while knockdown of *EGFR* tended to increase DHRS7 expression. Importantly, DHRS7 expression negatively correlates with EGFR expression and positively with survival rates in prostate cancer patients. This study suggests a tumor-suppressor role for DHRS7 by modulating EGFR expression in prostate cancer.

**Abstract:**

Prostate cancer (PCa), one of the most common malignancies in men, typically responds to initial treatment, but resistance to therapy often leads to metastases and death. The dehydrogenase/reductase 7 (DHRS7, SDR34C1) is an “orphan” enzyme without known physiological function. DHRS7 was previously found to be decreased in higher-stage PCa, and siRNA-mediated knockdown increased the aggressiveness of LNCaP cells. To further explore the role of DHRS7 in PCa, we analyzed the proteome of LNCaP cells following *DHRS7* knockdown to assess potentially altered pathways. Although DHRS7 is able to inactivate 5α-dihydrotestosterone, *DHRS7* knockdown did not affect androgen receptor (AR) target gene expression, and its effect on PCa cells seems to be androgen-independent. Importantly, proteome analyses revealed increased expression of epidermal growth factor receptor (EGFR), which was confirmed by RT-qPCR and Western blotting. Comparison of AR-positive LNCaP with AR-negative PC-3 and DU145 PCa cell lines revealed a negative correlation between DHRS7 and EGFR expression. Conversely, *EGFR* knockdown enhanced DHRS7 expression in these cells. Importantly, analysis of patient samples revealed a negative correlation between DHRS7 and EGFR expression, both at the mRNA and protein levels, and DHRS7 expression correlated positively with patient survival rates. These results suggest a protective role for DHRS7 in PCa.

## 1. Introduction

Prostate cancer (PCa) is the most common cancer and the third leading cause of cancer-related deaths in men in Europe [1]. PCa treatment needs to take into account several patient- and disease-related factors. When the tumor is still confined to the prostate, prostatectomy and radiotherapy are frequently undertaken in a curative attempt. However, when it is no longer confined to the primary site, these treatments are often not successful, with nearly half of the patients showing a rise in the PCa biomarker, prostate-specific antigen (PSA), thus requiring further therapy [2].

Because PCa generally depends on androgens and androgen receptor (AR)-mediated signaling, initial interventions include hormonal therapy using antiandrogenic drugs and chemical castration to disrupt androgen-mediated signaling [2,3]. Androgen deprivation therapy initially leads to clinical improvement in hormone-sensitive PCa; however, it eventually fails in most patients. This can be explained by several mechanisms, including alternative routes of androgen production from adrenal precursors, diminished androgen inactivation, enhanced AR sensitivity, and AR-independent signaling pathways [3]. Once hormonal therapy is no longer effective, the cancer is commonly referred to as “castration-resistant PCa (CRPC)”, which is currently considered non-curable, and available treatment options, e.g., chemotherapy, are limited [2]. Thus, research is needed to identify new players and uncover mechanisms and pathways involved in PCa progression that potentially offer new therapeutic options.

The initial androgen-dependence of PCa raised considerable attention to enzymes involved in androgen metabolism. The intratumoral formation of potent androgens may render advanced forms of PCa independent of the circulating levels of testosterone and 5α-dihydrotestosterone (5α-DHT). Several members of the short-chain dehydrogenase/reductase (SDR) superfamily were shown to locally activate androgens, including hydroxysteroid dehydrogenase (HSD) 17B3 (SDR12C2) converting androstenedione to testosterone [4,5], and the 3α-HSDs converting 3α-androstanediol (3α-Adiol) to 5α-DHT, i.e., HSD17B6 (SDR9C6), retinol dehydrogenase (RDH) 5 (SDR9C5), RDH16 (SDR9C8), and DHRS9 (SDR9C4) [6,7,8]. Additionally, HSD3B1 (SDR11E1), required for the production of intratumoral androgens from adrenal precursors, has been associated with CRPC [9].

Another SDR enzyme, dehydrogenase/reductase 7 (DHRS7, SDR34C1), is present in the prostate and metabolizes androgens although its physiological function remains unknown [10,11,12,13]. In vitro experiments revealed a preference for cofactor NADPH over NADH to catalyze the reduction of a variety of substrates when used at high concentrations, including xenobiotics, all-*trans*-retinal and steroid hormones, such as cortisone and androstenedione [11,14]. Later, experiments with HEK-293 cells overexpressing DHRS7 showed conversion of cortisone to 20β-dehydrocortisone and of 5α-DHT to 3α-Adiol [15]. Furthermore, AR-dependent luciferase activity was decreased upon coexpression with DHRS7 although only at 5α-DHT concentrations of 1 nM and higher. Later, this was supported by in silico screening, based on a structural homology model of DHRS7, and in vitro testing [16]. Together, these results suggested that DHRS7 may act as a tumor suppressor in early-stage PCa by lowering intratumoral 5α-DHT concentrations. A tumor-suppressor role of DHRS7 was further supported by observations that DHRS7 expression is abolished in metastatic PCa compared to primary PCa, and its expression declines with PCa progression as measured by the Gleason pattern [12,17]. The latter study also showed that siRNA-mediated *DHRS7* knockdown in PCa cells leads to increased proliferation and migration and decreased adhesion. Nevertheless, DHRS7 was unable to prevent AR transactivation at subnanomolar concentrations, reflecting physiological levels, and *DHRS7* knockdown increased cell migration and decreased the adhesion of AR-negative PC-3 cells, indicating the existence of the androgen-independent functions of DHRS7.

This study aimed to further explore the role of DHRS7 in PCa by investigating whether the effects of *DHRS7* knockdown on gene expression in LNCaP cells are androgen-dependent. For this purpose, the expressions of three AR-target genes were measured after *DHRS7* knockdown and incubation with 5α-DHT in LNCaP cells. Second, a proteomics experiment in LNCaP cells after *DHRS7* knockdown was performed to identify genes and/or pathways affected by DHRS7. KEGG pathway annotation of the proteome data yielded 48 proteins annotated with PCa, including the transmembrane tyrosine kinase, epidermal growth factor receptor (EGFR). This receptor is known to play a key role in various cancers, including non-small-cell lung, breast, and colorectal cancer, which led to the approval of both small-molecule tyrosine kinase inhibitors and antibodies targeting EGFR [18]. Furthermore, EGFR is considered to be a master regulator of many signaling pathways, modulating many biological processes that are important for cell growth and survival [19]. EGFR activation affects the expression and activity of various proteins, including signal transducer and activator of transcription (STAT) 3 [19,20,21,22,23], which was also upregulated in the proteome data. For these reasons, EGFR and STAT3 were further studied in LNCaP, PC-3, and DU145 cells. In addition, we tested a possible correlation between DHRS7 and EGFR expression in samples from PCa patients by analyzing *The Cancer Genome Atlas (TCGA)* database and by employing PCa tissue microarrays (TMA). Finally, we also analyzed whether the high- and low-expression status of DHRS7 and EGFR were associated with patient survival rates.

## 2. Materials and Methods

### 2.1. Chemicals and Reagents

LNCaP (Cat# CRL-1740, RRID: CVCL_1379), PC-3 (Cat# CRL-1435, RRID: CVCL_0035), and DU145 (Cat# HTB-81, RRID: CVCL_0105) cells were purchased from American Type Culture Collection (Manassas, VA, USA). EGF (E9644), KAPA SYBR FAST qPCR Master Mix (KK4618), RPMI-1640 (R8758), cOmplete Mini (11 836 153 001), cOmplete (04 693 116 001) Protease Inhibitor Cocktails, Minimum Essential Medium (51416C), mouse polyclonal anti-DHRS7 antibody for TMA (Cat# HPA031121, Lot# R30582, RRID: AB_10600803), and 5α-DHT (10300-1G-F) were obtained from Sigma-Aldrich (St. Louis, MO, USA). siRNAs were purchased from Horizon Discovery (Cambridge, UK; Table S1), and oligonucleotide primers for RT-qPCR were purchased from Microsynth (Balgach, Switzerland; see Table S2). RapidOut DNA Removal Kit (K2981), Pierce bicinchoninic acid assay (23225), the EASY-nLC 1200 system, the Orbitrap Fusion Lumos instrument, and Proteome Discoverer 2.2 software were purchased from Thermo Fisher Scientific (Waltham, MA, USA). Lipofectamine RNAiMAX (13778-150) was bought from Thermo Fisher Scientific; fetal bovine serum (FBS; S1810) was purchased from Biowest (Nuaillé, France); and QIAcube-equipment, RNeasy Mini Kit (74106), and the Rotor-Gene Q were purchased from Qiagen (Venlo, The Netherlands). A NanoDrop One^C^ was purchased from Witec AG (Sursee, Switzerland); a protein assay kit for Bradford Assay was purchased from Bio-Rad Laboratories (Hercules, CA, USA); a PVDF membrane (IPVH00010) and an Immobilon Western horseradish peroxidase (HRP) substrate kit (WBKLS0500) were purchased from Merck KGaA (Darmstadt, Germany); and Ham’s F-12K Medium (21127022) and Opti-MEM (51985-026) were purchased from Gibco (Life Technologies, Carlsbad, CA, USA). The Fusion FX instrument was purchased from Vilber (Collégien, France), and Lysyl endopeptidase (LysC) was purchased from Wako Chemicals (Neuss, Germany). Rabbit monoclonal anti-EGFR antibody (Cat# 790-4347, Clone 5B7, RRID: AB_2617183), rabbit anti-AR antibody (Cat#760-4605, clone SP107, RRID: not available) for TMA, and a Ventana BenchMark immunostainer were purchased from Ventana (Roche, Basel, Switzerland). A BOND-III immunohistochemistry staining system and BOND Polymer Refine Detection kit (Cat# DS9800) were bought from Leica Biosystems (Wetzlar, Germany); the Takara PrimeScript RT reagent kit (RR037A) was purchased from Takara Bio, Inc. (Kusatsu, Japan); and a GraphPad Prism was purchased from GraphPad Software (RRID: SCR_002798, San Diego, CA, USA). Sep-Pak C18 cartridges, packed with 50 mg Sorbent (WAT054955), were obtained from Waters (Milford, MA, USA). A Vydac 218TPN C18 column (250 × 1 mm, 300 Å, 5 µm particle size) and ReproSil-Pur 120 C18-AQ reverse phase material (2.4 µm particle size) were purchased from Dr. Maisch GmbH (Ammerbuch-Entringen, Germany), and PD-10 desalting columns were purchased from GE Healthcare (Chicago, IL, USA). All other chemicals and reagents were obtained from either Sigma-Aldrich or AppliChem GmbH (Darmstadt, Germany). Antibodies used for Western blotting are listed in Table S3.

### 2.2. Cell Culture

LNCaP cells were cultured in RPMI-1640, PC-3 in Ham’s F-12K, and DU145 in Minimum Essential Medium. All cell culture media were supplemented with 10% FBS, 100 U/mL penicillin, 0.1 mg/mL streptomycin, and Minimum Essential Medium with 2 mM L-glutamine and 1 mM sodium pyruvate (final concentrations). All cell lines were cultured in a 5% CO_2_ atmosphere at 37 °C and passaged at roughly 70–80% confluence by rinsing with PBS, followed by trypsinization. For the experiments using 5α-DHT, 44 h after *DHRS7* knockdown, cells were incubated in serum-free medium for 4 h, followed by treatment with 5α-DHT in fresh serum-free medium for 24 h. Treatment with EGF (100 ng/mL for 1 or 5 min) was performed after the incubation of the cells for 4 h in serum-free medium, which was equivalent to 52 h after *DHRS7* knockdown.

### 2.3. Knockdown Using siRNA

Knockdown experiments were performed by the transfection of cells (100,000 cells/mL), with 3.75 µL siRNA and 7.5 µL Lipofectamine RNAiMAX in 200 µL Opti-MEM for 6-well plates, or with 12.5 µL siRNA and 15 µL Lipofectamine RNAiMAX in 2 mL Opti-MEM for 10 cm dishes. The final siRNA concentration was 25 nM. The siRNA sequence-targeting *DHRS7* was selected from three others based on a previous study [12]. For *EGFR* knockdown, siRNA-3 was used. Non-targeting siRNA was used as a control.

### 2.4. Gene Expression Analysis by RT-qPCR

Total RNA was isolated using a QIAcube and an RNeasy Mini Kit (following the protocol for animal tissues and cells). RNA was quantified by a NanoDrop One^C^, and genomic DNA was removed using a RapidOut DNase Removal Kit. RNA (750–1000 ng) was reverse-transcribed to cDNA using a Takara PrimeScript RT reagent kit according to the manufacturer’s instructions. RT-qPCR was performed in a Rotor-Gene Q, using KAPA SYBR FAST qPCR Master Mix with 4 ng of cDNA as a template and 200 nM of oligonucleotide primers. Runs were started at 95 °C for 5 min, followed by 40 cycles (10 s at 95 °C, 15 s at 60 °C, and 20 s at 72 °C), and finalized with a melting ramp (72 °C to 95 °C, with 1° C increments every 5 s). Ct-values were normalized to the housekeeping gene *PPIA* (2^−(Ct gene of interest−Ct *PPIA*)^). In experiments including *EGFR* knockdown, *GAPDH* was used as a housekeeping gene because *PPIA* expression was altered, and this gene did not, therefore, qualify as a control under this condition.

### 2.5. Protein Expression Analysis by Western Blotting

Cells were homogenized in RIPA buffer containing cOmplete Mini protease inhibitor, centrifuged (16,100× *g*, 10 min, 4 °C), and the supernatant was collected. Protein concentration was assessed using the Pierce bicinchoninic acid assay according to the manufacturer’s instructions. Samples were reduced by boiling (95 °C, 5 min) in Laemmli buffer (5 mM Tris-HCl, pH 6.8, 10% glycerol [*v*/*v*], 0.2% sodium dodecyl sulfate [*w*/*v*], 1% bromophenol blue [*w*/*v*], and 5% β-mercaptoethanol [*v*/*v*]) and stored at −20 °C. Proteins (20 µg) were separated by 7.5–12.5% SDS-PAGE and transferred to PVDF membranes. the membranes were blocked in 10% (*w*/*v*) commercially available, defatted milk in TBS-T (1.11 g/L Tween 20, 137 mM NaCl, and 20 mM Trizma Base, pH 7.6) for 1 h at room temperature. Membranes were incubated with primary antibodies overnight at 4 °C and with secondary antibodies for 1 h at room temperature in 5% defatted milk in TBS-T. Antibody-labeled proteins were visualized using an Immobilon Western Chemiluminescence HRP substrate kit in a Fusion FX instrument. Relative protein expression was assessed by densitometry using the Fusion FX-associated EVOLUTION-CAPT software. The antibody dilutions applied in the experiments are listed in Table S3.

### 2.6. Proteome Analyses in LNCaP Cells

LNCaP cells subjected for 24 h, 36 h, or 48 h to *DHRS7* knockdown in 10 cm dishes were washed 3 times with ice-cold PBS and homogenized for 10 min in 1 mL of ice-cold urea lysis buffer (8 M urea, 50 mM Tris-HCl pH 8.0, 75 mM NaCl, and 1 mM PMSF) containing cOmplete protease inhibitors. Cells were then sonicated (3 × 60 s, 4 °C, 20% output, 2 min rest between cycles), centrifuged (12,000× *g*, 10 min, 4 °C), and the supernatant was stored at −80 °C. Protein concentration was measured by Bradford assay. The lysate was reduced (10 mM DTT, 55 °C, 30 min) and alkylated (50 mM iodoacetamide, room temperature, 15 min, in the dark). The reaction was stopped by adding β-mercaptoethanol (0.33% [*v*/*v*]). Lysates were desalted on PD-10 columns, which had been previously equilibrated with 20 mL of 4 M urea, 50 mM Tris-HCl pH 8.0, and 150 mM NaCl, and the proteins were eluted with 4 M urea buffer with 50 mM Tris-HCl pH 8.0 and 75 mM NaCl in 10 fractions of 1 mL each. Protein-containing fractions (as measured by 280 nm absorbance) were pooled, and protein concentration was determined using the Bradford assay. The samples were stored at −20 °C. Samples were digested with endoproteinase LysC (2 × 2 h, 37 °C, enzyme-to-protein ratio 1:100 [*w*/*w*]), and the urea concentration was lowered to 2 M with 50 mM Tris-HCl pH 8.0 and 75 mM NaCl, followed by trypsin digestion. (The first trypsin digestion lasted for 2 h, at 37 °C, with an enzyme-to-protein ratio 1:100 [*w*/*w*], followed by a second trypsin aliquot and overnight incubation at 37 °C.) Trifluoroacetic acid (TFA, 1% [*v*/*v*]) was used to stop the digestion and to lower the pH < 2. Sep-Pak C18 cartridges, packed with 50 mg sorbent, were used to desalt the samples. The cartridges were primed with 250 µL methanol, then 250 µL 80% acetonitrile (ACN)/0.1% TFA and equilibrated (750 µL 0.1% TFA). Samples were applied to the cartridges and washed (1250 µL 0.1% TFA), and the peptides were eluted (350 µL 80% ACN/0.1% TFA). Absorbance at 280 nm was measured, and the peptide concentration was estimated as described [24]. An aliquot containing 150 µg protein per sample was dried in a SpeedVac and stored at −20 °C.

The dried peptides were dissolved in 55 µL solvent A (20 mM ammonium formate, pH 4.5) and injected onto a Vydac 218TPN C18 column (250 × 1 mm, 300 Å, 5 µm particle size) at 30 µL/min, which had been equilibrated with solvent A. Bound peptides were eluted at 30 µL/min with a linear gradient from 2% to 50% solvent B (20 mM ammonium formate, pH 4.5, 80% ACN) for 100 min. At 2-minute intervals, fractions were collected, and every sixth fraction of the peptide-containing part of the chromatogram was pooled to achieve an even distribution of peptides across all fractions. The peptide pools were dried in a SpeedVac and stored at −20 °C for later analysis.

Individual peptide pools were dissolved in 30 µL of 0.1% formic acid in water and separated on a nano HPLC column (75 µm × 30 cm, packed in-house with ReproSil C18-AQ reverse-phase material, 2.4 µm particle size). All pools were injected 3 times as technical replicates (2 µL injection volume). The mobile phase was solvent A (0.1% formic acid in water) and B (80% ACN/0.1% formic acid). An EASY-nLC 1200 system, set to 0.25 µL/min, provided a linear gradient of solvent B: 0% for 5 min, from 0% to 35% over the course of 155 min, to 100% over the course of 5 min, followed by 15 min at 100%. The column was connected to an Orbitrap Fusion Lumos instrument. The eluting peptides were ionized at 1.9 kV, and the instrument was operated in data-dependent mode with a cycle time of 3 s. Precursor scans in the Orbitrap were conducted from 400–1600 *m*/*z* at a resolution of 120,000, with a maximum injection time of 100 ms. The data-dependent MS2 scans were performed using the linear ion trap of the instrument and set to allow for the maximal number of MS2 scans to be collected, with an injection time of 35 ms. The monoisotopic precursor selection (MIPS) mode was set to “Peptide”, the intensity threshold to 5000, and the dynamic exclusion duration to 60 s. Only peptides with a charge state of 2–7 were included in the analysis.

Data obtained from the LC–MS/MS were searched against the SwissProt database using the Mascot and Sequest HT search engines of Proteome Discoverer 2.2. Precursor and fragment mass tolerance were set to 10 ppm and 0.6 Da, respectively. The peptide modifications methionine-oxidation and N-terminal acetylation were set to “dynamic”, and cysteine-carbamidomethylation was set to “static”. The confidence of the peptide matches was set to a 1% false-discovery rate. Relative protein quantification was performed using a Minora Feature Detector. The detected proteins were filtered according to the following criteria: master proteins and master protein candidates; high protein-false-discovery-rate confidence by either Mascot or Sequest HT; identification with at least 3 different peptides, whereby at least 1 peptide was uniquely assigned to the given protein; and proteins detected in all technical triplicates. The proteomics data sets of each time point were median-normalized using an additional LNCaP sample that was not subjected to any treatment or reverse transfection (seeded on a 10 cm dish and lysed after 24 h). Perseus software, version 1.6.14.0, was used to perform KEGG pathway annotation and heat-map generation (Z-score normalization was followed by hierarchical clustering with standard settings) [25,26].

### 2.7. TCGA Data Analysis

Expression data for 551 TCGA-PRAD samples were downloaded on 3 March 2020 using the TCGAbiolinks R package in the form of FPKM-UQ [27]. AR signaling scores were calculated, as previously reported, using the log2 (FPKM) of 30 genes that define the AR signaling pathway [28].

### 2.8. Tissue Microarray and Immunohistochemistry

A total of 124 primary, unselected prostate carcinomas treated at the University Hospital Basel between the years of 1986 and 2015 were included in this study. Two TMAs of these 124 tumors were constructed as previously described [29]. Each punch was derived from the center of the tumor in an area with no necrosis so that each TMA spot consisted of more than 50% tumor cells. For 40 cases, adjacent non-malignant tissue was selected from the same donor block. Data were collected retrospectively in a non-stratified and non-matched manner, including patient age, tumor diameter, tumor stage, Gleason grade, lymphovascular invasion, and clinical outcome. The clinical outcome measure of interest was overall survival time.

Immunohistochemical staining with anti-EGFR and anti-AR antibodies was performed on 4 µm-thick sections using a Benchmark immunostainer according to the manufacturer’s instructions. For immunohistochemical staining with anti-DHRS7 antibody (dilution 1:600), antigen retrieval was performed with citrate buffer (pH 6.0) on a Leica Bond III IHC staining system using the BOND Polymer Refine Detection kit according to the manufacturer’s instructions, which provides rabbit anti-mouse IgG and anti-rabbit poly-HRP-IgG as the secondary antibody and the DAB chromogen. Images were acquired using an Olympus BX46 microscope. Protein expression in tissues was evaluated and scored by a board-certified pathologist (C.E.), who was unaware of the clinical data. The expression was evaluated based on cytoplasmic and/or membrane staining for EGFR and DHRS7 and nuclear staining for AR. Each tumor was scored semi-quantitatively for each marker by multiplying the proportion (%) of positive cells with the staining intensity score (0, none; 1, weak; 2, moderate; 3, strong).

### 2.9. Statistical Analysis

To assess the correlation of gene expression in the *TCGA* data, Spearman’s correlation was calculated using all of the samples or with the samples divided according to the indicated groups.

For the TMA, all analyses were performed in R (Version 3.6.3) [30]. The expression scores were visualized in the ggplot, box plot, and ggscatter functions of the ggpubr package [31]. Statistical comparisons were performed using a *t*-test or Wilcoxon’s test where appropriate. Correlations were performed using the Spearman’s correlation coefficient.

Patient survival analysis was performed using the survminer package (version 0.4.8) [32]. Patients were grouped into high and low subgroups for DHRS7 and EGFR expression for overall survival (OS) and progression-free survival (PFS) using optimal cut-off values as calculated by the “surv-cutpoint” function of the survminer package, based on the maximally selected rank statistics algorithm. Survival times were evaluated using Kaplan–Meier survival curves, and differences were analyzed using a log-rank test. In the overall survival analysis, the distribution of patient age was compared between high- and low-expression groups of DHRS7 and EGFR using a two-tailed *t*-test.

For all other experiments, GraphPad Prism software (version 5.04) was used for statistical evaluation by one- or two-way ANOVA, with a Bonferroni multiple comparison post-test or two-tailed *t*-test, as indicated. A *p*-value of <0.05 was considered as indicating a statistically significant difference.

## 3. Results

### 3.1. Knockdown of DHRS7 Does Not Alter AR-Regulated Gene Expression

**Figure 1:**
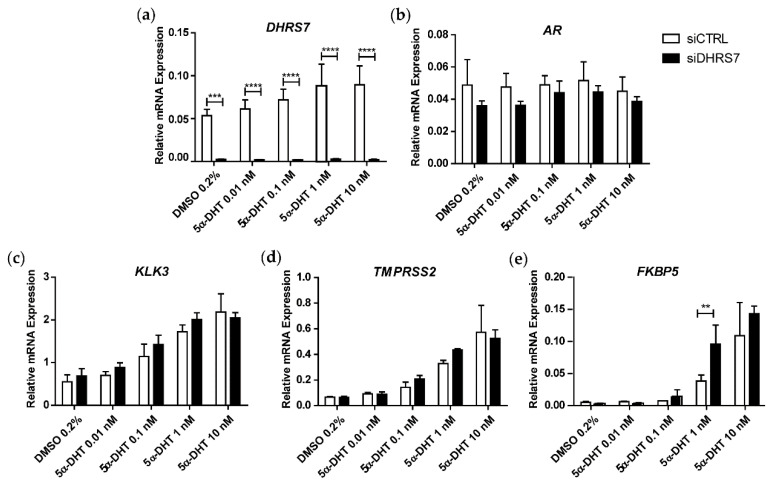
Effect of *DHRS7* knockdown on mRNA expression of *AR* and AR target genes. Comparison of mRNA expression relative to *PPIA* after treatment of LNCaP cells with siRNA against *DHRS7* (siDHRS7) or non-targeting siRNA control (siCTRL) for 48 h and incubation with different concentrations of 5α-DHT for another 24 h: (**a**) *DHRS7*; (**b**) *AR*; (**c**) *KLK3*; (**d**) *TMPRSS2*; and (**e**) *FKBP5*. Data represent mean ± SD of 3 independent experiments (n = 3). Statistical significance was calculated using two-way ANOVA with a Bonferroni multiple comparison post-test. ** *p* < 0.01; *** *p* < 0.001; **** *p* < 0.0001.

Based on the previous observation that DHRS7 is capable of metabolizing the potent AR agonist 5α-DHT to the inactive 3α-Adiol [15], we first tested AR-dependence and investigated whether 5α-DHT treatment following knockdown of *DHRS7* in AR-positive, androgen-dependent LNCaP cells would increase the expression of the AR target genes *KLK3*, *TMPRSS2,* and *FKBP5* [33]. For this purpose, *DHRS7* was knocked-down using siRNA for 48 h before incubation for another 24 h in the presence of 5α-DHT at the concentration indicated. As expected, *DHRS7* mRNA expression was abolished. However, although a concentration-dependent increase of the analyzed AR-regulated genes could be observed, there were no substantial differences between the control and *DHRS7* knockdown groups, except for *FKBP5* at 1 nM of 5α-DHT. Additionally, *AR* mRNA expression was unchanged or even tended to be decreased (Figure 1).

### 3.2. Proteomics Analyses Indicated That DHRS7 Knockdown Did Not Affect AR and AR Target Protein Expression but Did Increase EGFR and STAT3 Expression in LNCaP Cells

Because 5α-DHT treatment after *DHRS7* knockdown unexpectedly did not result in the upregulation of AR target genes, we adopted a proteomics approach to search for other altered pathways that could explain the more aggressive phenotype of LNCaP cells observed after *DHRS7* knockdown [12]. To assess protein changes over time, the proteome was analyzed 24 h, 36 h, and 48 h after *DHRS7* knockdown (Figure S1 and Files S1–S3).

We first wanted to corroborate the evidence from the mRNA expression measurements that AR target genes are not affected by *DHRS7* knockdown by inspecting the AR and its targets KLK3, TMPRSS2, and FKBP5. As shown in Figure S2a, DHRS7 expression gradually decreased with time following the knockdown, whereas the expression of AR and FKBP5 changed only marginally. KLK3 and TMPRSS2 expression seemed to increase with elapsing time, irrespective of *DHRS7* knockdown. Upon knockdown, however, KLK3 and TMPRSS2 expression tended to be lower. Because the proteome analysis was performed only once at three different time points as a screening tool to generate hypotheses, we validated the obtained results on the mRNA and protein level using RT-qPCR and Western blotting. Whereas *DHRS7* knockdown was evident on the mRNA and protein level, no changes were observed for AR, KLK3, TMPRSS2, or FKBP5 (Figure S2b–d).

**Figure 2:**
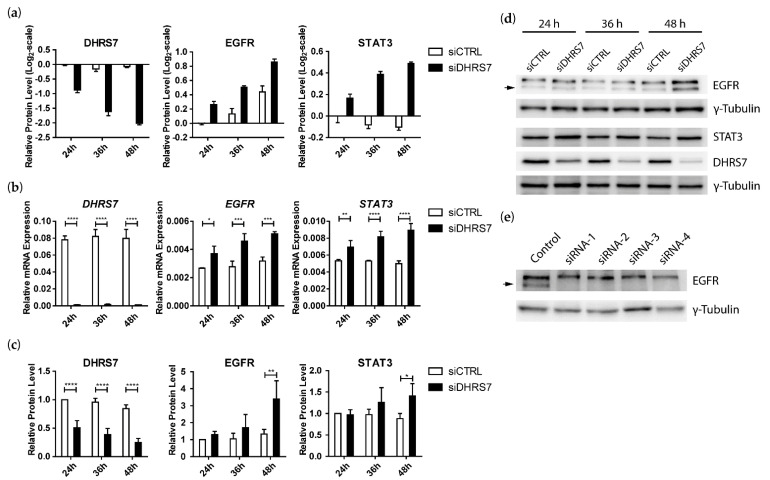
Effect of *DHRS7* knockdown on EGFR and STAT3 expression in LNCaP cells. Knockdown of *DHRS7* in LNCaP cells was performed for 24 h, 36 h, and 48 h. (**a**) Relative protein expression from the proteomics data sets. Control at 24 h was set to zero, and all other data sets were compared to the control at 24 h. (**b**) mRNA expression levels relative to *PPIA*. (**c**) Densitometry analysis of protein-expression levels normalized to control at 24 h and γ-Tubulin. (**d**) Representative Western blot (1 of 3) with γ-Tubulin as a loading control. (**e**) Knockdown of *EGFR* using 4 different siRNAs (n = 1). Data represent the mean ± SD of 1 experiment for the proteomics data (n = 1, 3 technical replicates, panel (**a**)) and mean ± SD of 3 independent experiments for mRNA and densitometry (n = 3, panel (**b**,**c**)). siCTRL = non-targeting siRNA control; siDHRS7 = siRNA against *DHRS7*. For mRNA and densitometry results, statistical significance was calculated using two-way ANOVA with a Bonferroni multiple comparison post-test. * *p* < 0.05; ** *p* < 0.01; *** *p* < 0.001; **** *p* < 0.0001.

Next, because we were interested in pathway alterations related to PCa, we performed KEGG pathway annotation and searched for proteins annotated with “prostate cancer”. Of the 6675 proteins that were detected at all time points, 48 proteins were annotated with the term “prostate cancer” (Figure S3 and File S4). Most of these proteins showed rather minor changes (log_2_-fold change < 0.5) following *DHRS7* knockdown, yet several proteins of known pathways seemed to be altered. Of the PI3K/AKT pathway, the PI3K catalytic subunit PIK3CB and the PI3K regulatory subunit PIK3R2 were elevated, but not AKT1 or AKT2. Among the ERK/MAPK pathway, MAP2K2 and MAPK1 were found to be lower after *DHRS7* knockdown. Additionally, the cyclin-dependent kinase CDK2 and the cyclin-dependent kinase inhibitor CDKN1A were both downregulated 36 h and 48 h after *DHRS7* knockdown. Also, the protein levels of the transcriptional modulator CREB3L4, β-Catenin (CTNNB1), the transcriptional corepressor RB1, and the heat-shock protein HSP90AA1 were decreased following *DHRS7* knockdown, while the transcription factor NKX3-1 and the NFκB-pathway member NFKB1 were found to be upregulated. Interestingly, an upregulation of the EGFR was clearly evident at all 3 time points analyzed (Figure 2a and Figure S3).

In the present study, we decided to further analyze the effect of *DHRS7* knockdown on EGFR because of its importance in cell signaling and cancer treatment [18,19,23]. Analysis of our proteomics data revealed a time-dependent increase of STAT3, one of the downstream targets of the EGFR [20,21,22], after *DHRS7* knockdown (Figure 2a). RT-qPCR and Western blotting of samples of LNCaP cells subjected to *DHRS7* knockdown confirmed the time-dependent increase of EGFR and STAT3 expression seen in the proteomics data, which was statistically significant on the mRNA level at all time points and on the protein level after 48 h (Figure 2b–d). Because Western blotting showed 2 bands for EGFR, *EGFR* knockdown was performed for 48 h using four different siRNAs, revealing that only the lower band corresponds to EGFR (Figure 2e, indicated by an arrow).

### 3.3. Inverse Correlation of DHRS7 and EGFR Expression in LNCaP, PC-3, and DU145 Cells

**Figure 3:**
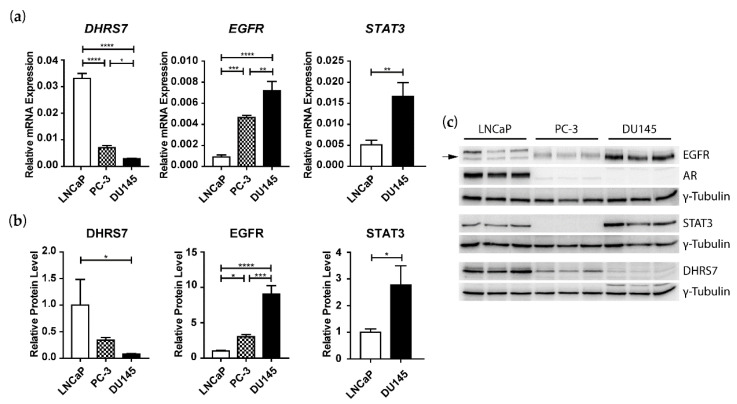
Comparison of DHRS7, EGFR, STAT3 and, AR expression in LNCaP, PC-3, and DU145 cells. (**a**) mRNA expression levels relative to *PPIA*. (**b**) Densitometry analysis of protein-expression levels normalized to LNCaP cells and γ-Tubulin. (**c**) Western blot of 3 individual samples per cell line, with γ-Tubulin as a loading control. Data represent the mean ± SD of 3 independent samples (n = 3). Statistical significance was calculated using one-way ANOVA with a Bonferroni multiple comparison post-test for DHRS7 and EGFR and a two-tailed *t*-test for STAT3. * *p* < 0.05; ** *p* < 0.01; *** *p* < 0.001; **** *p* < 0.0001.

Next, we tested whether *DHRS7* knockdown leads to increased EGFR expression in other PCa cell lines. A comparison of DHRS7 expression in LNCaP, PC-3, and DU145 cells revealed that DHRS7 shows the highest expression in LNCaP and at levels of approximately 80% and 90% lower in PC-3 and DU145, respectively (Figure 3). An inverse expression pattern was observed for EGFR, with the highest levels in DU145 and at levels about 60% and 90% lower in PC-3 and LNCaP, respectively. STAT3, which could not be detected in PC-3 cells, showed levels that were about 3-fold higher in DU145 compared to LNCaP cells, both at the mRNA and protein levels (Figure 3). The AR could only be detected in LNCaP cells, both at the mRNA and protein level.

**Figure 4:**
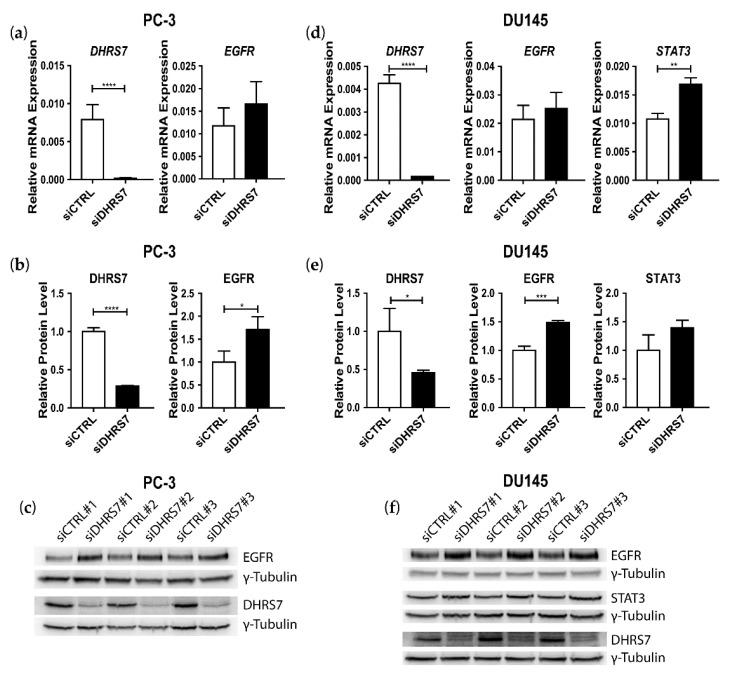
Effect of *DHRS7* knockdown on the expression of DHRS7, EGFR, and STAT3 in PC-3 and DU145 cells. (**a**,**d**) mRNA expression levels relative to *PPIA* in PC-3 and DU145 cells, respectively. (**b**,**e**) Densitometry analysis of protein-expression levels normalized to control and γ-Tubulin in PC-3 and DU145 cells, respectively. Data represent the mean ± SD of at least 3 independent experiments (n = 3–5). (**c**,**f**) Western blot from PC-3 and DU145 cells, respectively, with γ-Tubulin as a loading control. siCTRL = non-targeting siRNA control; siDHRS7 = siRNA against *DHRS7*. Statistical significance was calculated using a two-tailed *t*-test. * *p* < 0.05; ** *p* < 0.01; *** *p* < 0.001; **** *p* < 0.0001.

Next, we performed *DHRS7* knockdown in PC-3 and DU145 cells. DHRS7 mRNA and protein expression were efficiently diminished (Figure 4). In both PC-3 and DU145 cells, *DHRS7* knockdown led to a moderate trend increase in *EGFR* mRNA but a significant increase in EGFR protein expression. STAT3 showed elevated mRNA expression but only a trend increase at the protein level in DU145 cells.

### 3.4. Knockdown of EGFR Leads to Increased Expression of DHRS7

**Figure 5:**
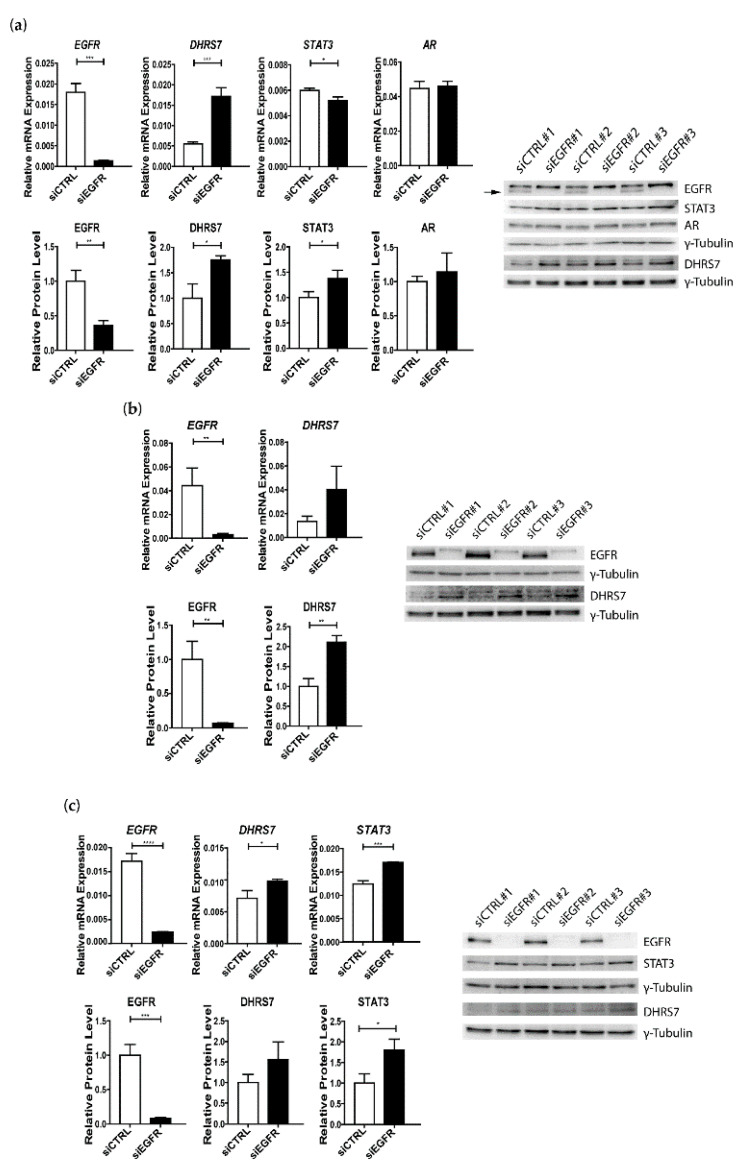
Effect of *EGFR* knockdown on the expression of DHRS7, STAT3, and AR in PCa cells. (**a**) LNCaP, (**b**) PC-3, and (**c**) DU145 cells. mRNA expression levels are relative to the *GAPDH* control. In the densitometry analysis, the protein-expression levels were normalized to the control and γ-Tubulin of the corresponding Western blots of 3 individual samples per cell line, with γ-Tubulin as a loading control. Data represent the mean ± SD of 3 independent samples (n = 3). siCTRL = non-targeting siRNA control; siEGFR = siRNA against *EGFR*. Statistical significance was calculated using a two-tailed *t*-test. * *p* < 0.05; ** *p* < 0.01; *** *p* < 0.001; **** *p* < 0.0001.

Because of the apparent inverse correlation between DHRS7 and EGFR expression in PCa cells and the increased EGFR expression after *DHRS7* knockdown, we wondered whether *EGFR* knockdown would impact DHRS7 expression. For this purpose, siRNA-3, which appeared to be most efficient in the experiment described above (Figure 2), was used for *EGFR* knockdown. Indeed, *EGFR* knockdown led to 3-fold and 1.7-fold higher DHRS7 expressions at the mRNA and protein levels, respectively, in LNCaP cells. STAT3 showed a moderate but significant downregulation at the mRNA but a significant upregulation at the protein level in knockdown compared to control cells (Figure 5a). AR expression remained unchanged. In PC-3 cells, *EGFR* knockdown led to an almost 2-fold trend increase in DHRS7 mRNA and a significant 2-fold increase in its protein level (Figure 5b). *EGFR* knockdown in DU145 cells resulted in a low, but significant, increase in DHRS7 mRNA expression and a weak trend increase in protein expression. STAT3 was significantly increased in DU145 cells, both at the mRNA and protein levels (Figure 5c).

### 3.5. Increased EGFR Expression after DHRS7 Knockdown Is Accompanied by Increased Phosphorylation after EGF Treatment

Based on the observed increase in EGFR expression following *DHRS7* knockdown in all three cell lines, we tested whether treatment of the cells with EGF leads to enhanced EGFR phosphorylation in DHRS7 depleted cells. For that purpose, cells were subjected to *DHRS7* knockdown for 48 h prior to incubation in a serum-free medium for 4 h and treatment with 100 ng/mL EGF for 1 min and 5 min, followed by Western blotting. Besides EGFR, we also analyzed STAT3 because of its elevation after *DHRS7* knockdown, as well as AKT and ERK1/2 phosphorylation in all 3 cell lines.

**Figure 6:**
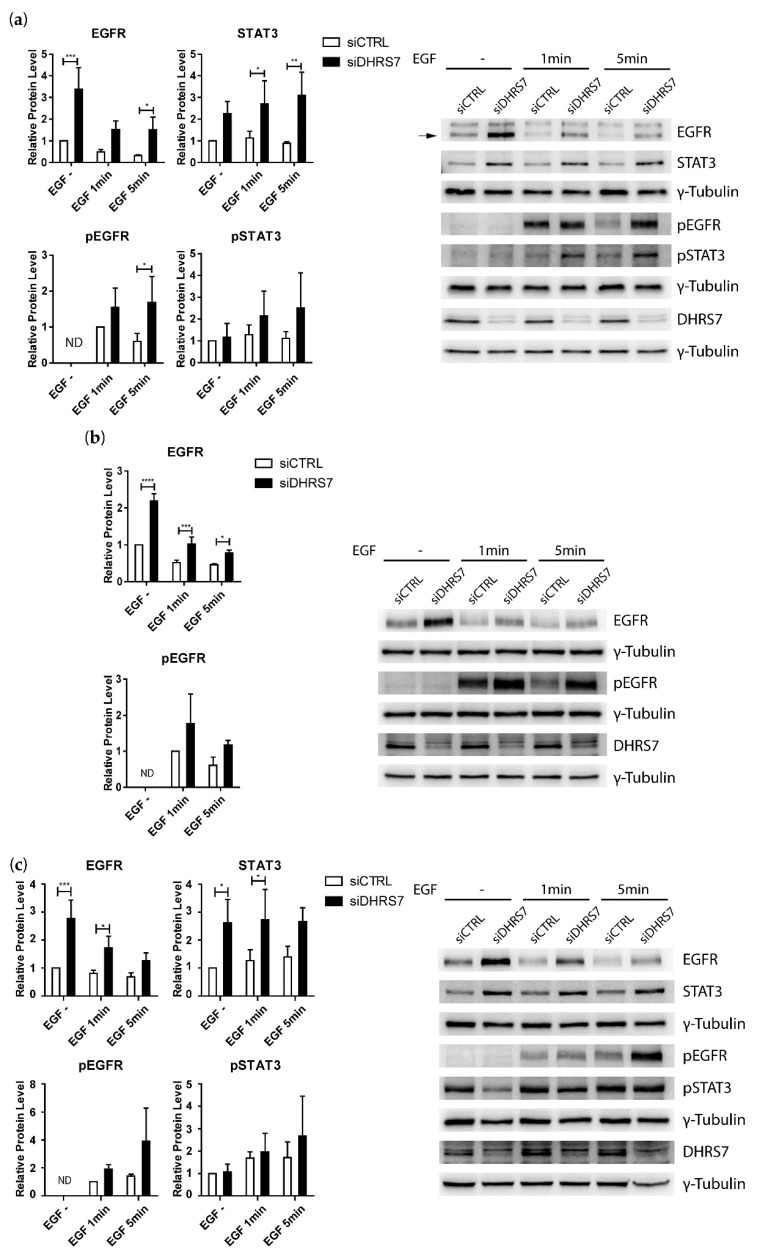
Effect of *DHRS7* knockdown on EGFR and STAT3 and their phosphorylation in PCa cells. (**a**) LNCaP, (**b**) PC-3, and (**c**) DU145 cells treated with EGF. Densitometry analysis of protein expression and phosphorylation levels were relative to control without treatment, or after 1 min of treatment where appropriate, and normalized to γ-Tubulin. Representative Western blots (one of three) are shown for each cell line, with γ-Tubulin as a loading control. Data represent the mean ± SD of 3 independent experiments (n = 3). siCTRL = non-targeting siRNA control; siDHRS7 = siRNA against *DHRS7*. Statistical significance was calculated using two-way ANOVA with a Bonferroni multiple comparison post-test. * *p* < 0.05; ** *p* < 0.01; *** *p* < 0.001; **** *p* < 0.0001.

Compared to the control, *DHRS7* knockdown induced EGFR expression in all 3 cell lines, consistent with the experiments described above, but the treatment with EGF for 1 min and 5 min resulted in reduced EGFR expression (Figure 6a–c), which might indicate decreased protein stability upon the ligand-dependent activation and internalization of the receptor. Importantly, EGFR phosphorylation was elevated after knockdown as compared to control cells. This trend was evident in all cell lines after 1 min and 5 min of EGF treatment, reaching statistical significance in LNCaP cells after 5 min of treatment. The expression of STAT3 in LNCaP and DU145 cells was increased after *DHRS7* knockdown, as compared to control cells, again consistent with the experiments described above. EGF treatment led to a trend increase in STAT3 phosphorylation in LNCaP cells and a weak trend increase in DU145 after 5 min of treatment (Figure 6a,c).

There was no difference in ERK expression between knockdown and control cells in any of the 3 cell lines investigated (Figure S4). A change in ERK phosphorylation after EGF treatment was observed in PC-3 cells only, showing a significantly lower phosphorylation level after *DHRS7* knockdown. No difference between knockdown and control cells could be observed for total AKT expression. Phosphorylation at serine 473 (S473) was significantly higher in LNCaP cells after 5 min of EGF treatment, but no difference was observed for threonine 308 (T308) phosphorylation. While EGF treatment induced phosphorylation of AKT at both residues irrespective of *DHRS7* knockdown in PC-3 cells, a trend increase in S473 phosphorylation and a significantly higher level of T308 phosphorylation could be detected after 1 min and 5 min of EGF treatment in DU145 cells when comparing knockdown and control cells.

A comparison of ERK and AKT expression in LNCaP, PC-3, and DU145 cells showed no significant difference for ERK protein expression among the 3 cell lines, whereas AKT expression tended to be higher in PC-3 and was higher in DU145 compared to LNCaP cells (Figure S5).

### 3.6. Analysis of the TCGA Database Revealed an Inverse Correlation between DHRS7 and EGFR Expression

**Figure 7:**
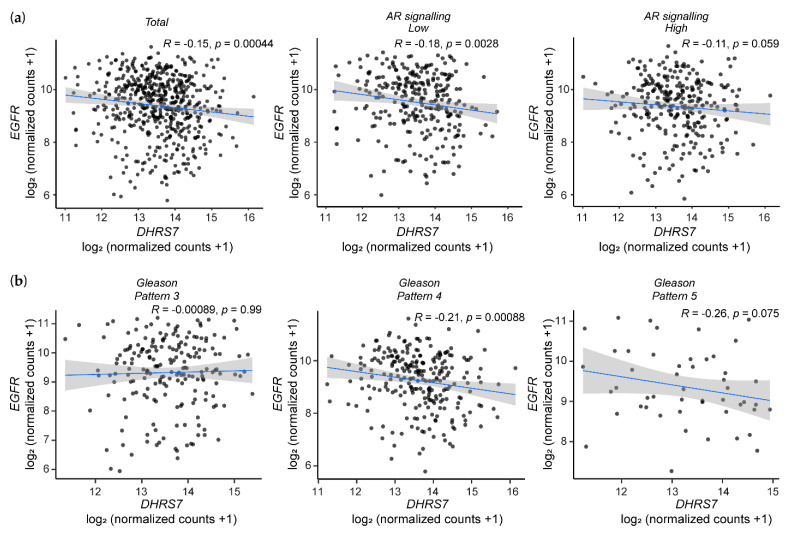
Results of *TCGA* data analysis. Correlation of *DHRS7* and *EGFR* mRNA expression in PCa samples of (**a**) the total dataset (n = 551) and according to AR signaling (median separated, n = 275 each) of the *TCGA* database analysis; and (**b**) according to the Gleason pattern (n = 197, 250, and 49, respectively).

A previous study showed a negative correlation between DHRS7 expression and Gleason pattern in samples from PCa patients [12], and EGFR expression has repeatedly been reported to be increased in higher stages of PCa [34]. Based on our in vitro findings, we hypothesized an inverse correlation between DHRS7 and EGFR expression in PCa. Analysis of mRNA expression using the *TCGA* database revealed a moderate but significant inverse correlation between *DHRS7* and *EGFR* expression (Figure 7a). Taking into account that PCa ultimately becomes AR-independent, and that EGFR expression increases with the progression of PCa [3,34], we stratified the *TCGA* data according to AR signaling into a high and a low group by median. A slightly increased inverse correlation was obtained for the data with lower AR signaling and a less-pronounced inverse correlation for the data set of high AR signaling. Furthermore, we investigated whether the inverse correlation of *DHRS7* and *EGFR* expression is more pronounced with PCa progression. Stratification of the *TCGA* data according to the Gleason pattern showed no correlation between *DHRS7* and *EGFR* expression for patients with the lowest Gleason pattern (Gleason pattern 3) (Figure 7b); however, patients with a Gleason pattern of 4 showed a weak, yet statistically significant, inverse correlation, and this was even slightly more pronounced for patients with Gleason pattern 5 although, due to fewer data, statistical significance was not reached. The mRNA expression of *DHRS7* and *STAT3* did not correlate but *EGFR* and *STAT3* mRNA expression showed a strong correlation (Figure S6).

### 3.7. Correlation of Lower DHRS7 and Higher EGFR Expression with Gleason Score and Reduced Survival Probability

**Figure 8:**
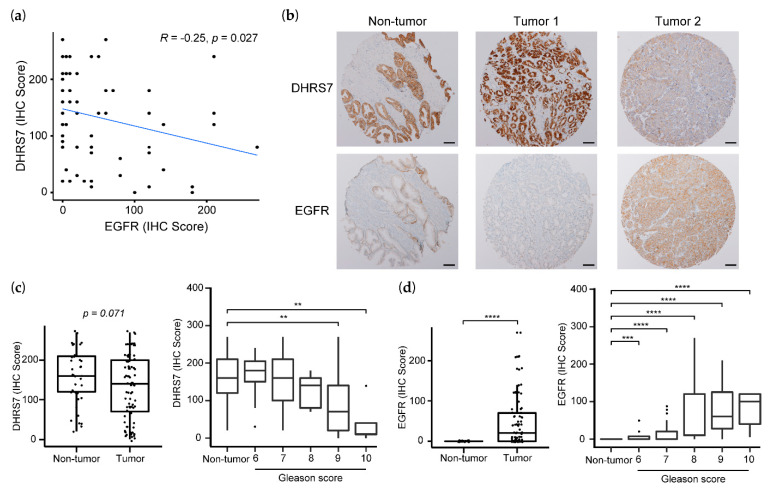
Correlation of DHRS7 and EGFR expression in PCa based on the analyses of TMA. (**a**) Correlation of DHRS7 and EGFR protein expression in PCa samples of the TMA analysis. (**b**) Representative staining results of the TMA in non-tumor and tumor samples (scale bar 100 µm). Expression of DHRS7 (**c**) and EGFR (**d**) in non-tumor and tumor samples, total and grouped by Gleason score. The correlation was assessed using Spearman’s correlation. Statistical significance was calculated by a two-tailed *t*-test for comparing total numbers of non-tumor and tumor samples and by a two-sided Wilcoxon’s test for associations with Gleason score. ** *p* < 0.01; *** *p* < 0.001; **** *p* < 0.0001.

To assess whether the correlation at the mRNA level can also be seen at the protein level, we stained TMA slides for DHRS7, EGFR, and AR and observed an inverse correlation between DHRS7 and EGFR expression (Figure 8a). A stratification based on AR expression (high or low expression groups, either by median or mean) did not improve the correlation. Representative tumor slides are shown in Figure 8b. Overall, DHRS7 expression was higher in non-tumor samples compared to tumor samples (Figure 8c) and, accordingly, EGFR expression was higher in tumor samples compared to non-tumor prostate samples (Figure 8d). Stratification by Gleason score revealed a gradual decline of DHRS7 expression, whereby the difference between non-tumor samples and tumor samples with Gleason scores of 9 or 10 was statistically significant. In contrast, EGFR expression showed a gradual increase with Gleason score, and the expression in non-tumor samples as compared to tumor samples of any Gleason score was statistically significantly different (Figure 8d). This is consistent with the more-pronounced inverse correlation of *DHRS7* and *EGFR* expression within the *TCGA* data when stratified according to Gleason pattern.

**Figure 9:**
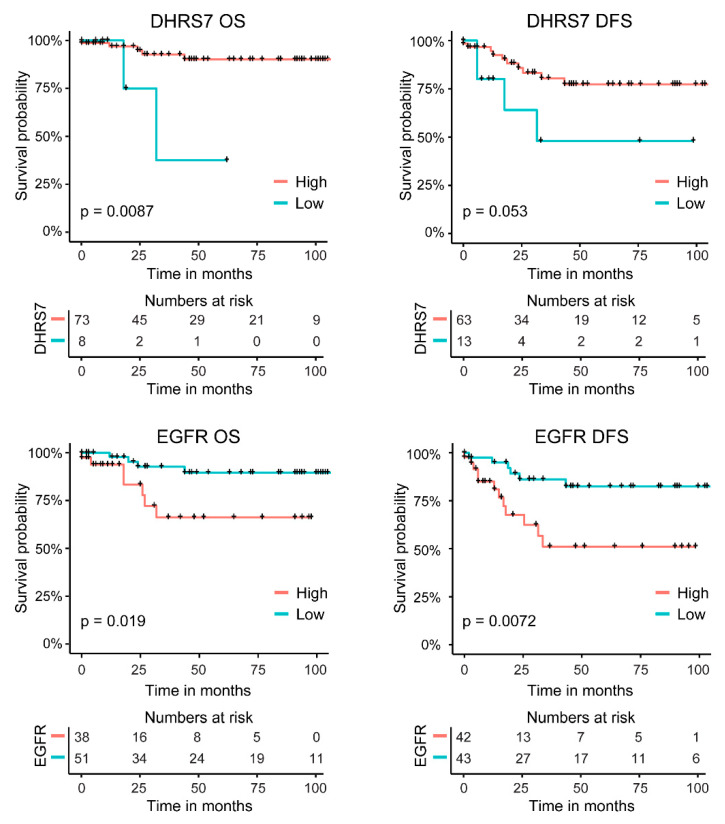
Correlation of DHRS7 and EGFR protein expression with survival probability. Kaplan–Meier curves showing the overall and disease-free survival probabilities of PCa patients with respect to DHRS7 and EGFR expression as derived by TMA staining. OS = overall survival; DFS = disease-free survival. Statistical significance was calculated using a log-rank test.

In addition, we analyzed the overall and disease-free survival rates of the patients in relation to the expression of DHRS7 and EGFR. High expression of DHRS7 was associated with higher overall survival probability (*p*-value < 0.01) and showed a trend of being associated with disease-free survival probability (*p*-value = 0.053); in contrast, high expression of EGFR was associated with worse survival probabilities; both overall (*p*-value < 0.05) and disease-free survival (*p*-value < 0.01) (Figure 9). Because the Gleason score is an indicator of PCa survival [35], we assessed whether the distribution of the various Gleason scores is similar in the high and low expression groups of DHRS7 and EGFR. All patients in the low DHRS7 expression groups had a Gleason score of either 9 or 10, which was different from the more even distribution of patients in the high expression group across the Gleason scores of 6–10. The majority of patients in the low EGFR expression group, which had a higher survival probability, presented a Gleason score of 6 or 7, whereas the majority of the patients in the high expression group showed a Gleason score of 9 (Table S4). In addition, we compared the patients’ ages between the low and high expression groups of DHRS7 and EGFR. Patients with a low expression of DHRS7 were statistically significantly older than patients with a high expression of DHRS7 (Figure S7), as opposed to EGFR. Patients with a low EGFR expression were significantly younger than those with a high EGFR expression.

## 4. Discussion

The previously reported association of decreased DHRS7 expression with higher Gleason-grade PCa emphasized the need to further investigate the role of this enzyme in PCa and to uncover its substrates [12]. Experiments using cell-based assays and purified enzymes then suggested that DHRS7 is a multifunctional enzyme and may accept various substrates, including xenobiotics, retinoids, and steroid hormones, such as 5α-DHT [11,14,15]. The latter is of particular interest since other SDR members were reported as playing a role in intracrinology by regulating intratissue and intratumoral androgen concentrations [4,6,7,36]. The finding that DHRS7 is able to inactivate 5α-DHT, the most potent AR ligand in men [3,7], to 3α-Adiol led to the hypothesis that DHRS7 acts as a tumor-suppressor in PCa by lowering intratumoral 5α-DHT levels [15].

Therefore, the initial aim of this study was to further evaluate the presumed role of DHRS7 in limiting AR activation by metabolizing 5α-DHT in a suitable, AR-dependent PCa cell model. The results indicate an androgen-independent effect of DHRS7 in PCa. Although treatment with 5α-DHT increased the expression of AR target genes in LNCaP cells in a concentration-dependent manner, *DHRS7* knockdown did not affect the expression of AR-regulated genes. This may be explained by an insufficient affinity of DHRS7 for 5α-DHT, thus leading to the inefficient protection of the AR. In line with this, cell-based experiments indicated that DHRS7 could only partially suppress AR transactivation at supraphysiological concentrations of 1 nM and higher [15]. The only statistically significant difference seen was an increase of *FKBP5* after *DHRS7* knockdown when treated with 1 nM of 5α-DHT. FKBP5 functions as a co-chaperone in protein-folding through its peptidyl-prolyl *cis-trans* isomerase activity and has been linked to stress response [37]. FKBP5 expression is not exclusively regulated by the AR, but also by progesterone and glucocorticoid receptors. The latter might be involved in the expression changes of FKBP5, as DHRS7 was shown to be able to convert glucocorticoids to their 20β-hydroxylated metabolites [15].

Based on the lack of differential expression of AR regulated genes, we then performed a proteomics experiment to identify potentially altered pathways that could help explain the previously reported, increased aggressive behavior of PCa cells following *DHRS7* knockdown [12]. The proteome analyses did not detect any differences in the expression of AR and proteins that are typically increased by higher AR activity (*e.g.,* KLK3, TMPRSS2, and FKBP5), thus supporting the evidence that the effects observed after *DHRS7* knockdown in LNCaP cells are androgen-independent. Instead, KEGG pathway annotation and filtering for proteins associated with “prostate cancer” revealed several proteins, like EGFR, which may help to link DHRS7 to PCa. Follow-up experiments confirmed an increased expression of EGFR after *DHRS7* knockdown in AR-positive LNCaP cells, but also in AR-negative PC-3 and DU145 cells. This indicates that, even though DHRS7 is capable of metabolizing 5α-DHT to the weak androgen 3α-Adiol [15], this function does not seem to be involved in the more rapid proliferation of LNCaP cells after *DHRS7* knockdown [12]. Thus, we hypothesize that the observed effects on EGFR and PCa cell properties upon *DHRS7* downregulation are either due to the lack of the formation of a yet-unknown tumor suppressor or to the abolished inactivation of a promoter although ligand-independent effects mediated through protein–protein interactions cannot currently be excluded. Interestingly, a siRNA-mediated knockdown of *EGFR* led to an analogous increase of DHRS7 expression, suggesting the feedback regulation of EGFR on DHRS7 expression.

The results of the present study revealed a negative correlation between the expression of DHRS7 and the transmembrane tyrosine kinase EGFR, a receptor involved in various cancers, and which has increased expression in PCa disease progression [18,19,38,39]. This negative correlation was also evident in tumor samples of PCa patients, both at the mRNA and protein levels (Figure 7 and Figure 8). Interestingly, there is evidence for an inverse correlation between AR and EGFR expression in PCa disease progression [40], and a shift from AR towards EGFR expression could contribute to the resistance against AR targeting therapy in advanced PCa. Functional interaction between DHRS7 and EGFR is further evidenced by the fact that knockdown of *DHRS7* in PCa cells enhanced the EGF-mediated EGFR phosphorylation. The loss of DHRS7 parallels a loss of prostate epithelial structure and may be related to the epithelial-to-mesenchymal transition. During this transition, dependence on AR is often lost, and enhanced EGFR-mediated signaling may promote tumor progression. The role of DHRS7 in this process needs further investigation.

Moreover, our results showed that patient survival is increased for patients with higher DHRS7 expression levels (Figure 9). However, the number of patients with low DHRS7 expression was limited within our 2 cohorts; nevertheless, they all showed Gleason scores of 9 or 10 (Table S4), emphasizing that DHRS7 is lost in severe PCa tumors. The distribution of patients among the various Gleason scores was uneven when comparing high- and low-expression groups, for both DHRS7 and EGFR. Moreover, patients with lower DHRS7 expression, indicating lower survival probability, were older than patients with high expression. This was the opposite for EGFR expression, showing that patients with low EGFR expression were younger than patients with high expression. Due to the unequal distribution of patients with respect to Gleason score and age, the survival data must be interpreted with caution. Furthermore, as the number of patients with low DHRS7 expression was very low, additional studies with larger datasets will need to be performed to test whether DHRS7 expression might help to discriminate between Gleason score- and age-matched patients with different prognoses.

Antibodies against EGFR and small-molecule kinase inhibitors are both used to treat various cancers, including lung and colorectal cancer [18]. The EGFR could be an attractive target in advanced PCa. Phase II clinical trials investigated the use of the EGFR kinase inhibitors gefitinib, lapatinib, and erlotinib in PCa, obtaining a moderate response, but a number of patients showed stable disease [41,42,43,44]. Unfortunately, EGFR expression was not always assessed in these studies, and successful response to these therapies likely requires stratification of patients. A major pathway through which EGFR is mediating its effects includes PI3K/AKT [45]. The primary negative regulator of PI3K is PTEN, a gene that is frequently altered or completely lost in PCa [46,47]. In colorectal cancer, the response of EGFR to treatment with cetuximab is dependent on PTEN status, and in PC-3 cells, restoration of PTEN expression improved response to cetuximab-induced apoptosis and the inhibition of cell proliferation [48,49]. Thus, besides EGFR, patients should be stratified for their PTEN status to achieve a satisfactory response. In this regard, a small trial using EGFR-targeting cetuximab in combination with docetaxel indicated a better response, depending on EGFR and PTEN expression [50].

In cell-based experiments, we assessed the effect of *DHRS7* knockdown on the 3 key downstream mediators of EGFR, i.e., STAT3, AKT, and ERK, and we observed differences among the cell lines used in this study. In LNCaP cells, the signal from EGFR seems to be propagated via STAT3, as indicated by increased expression and a trend toward increased STAT3 phosphorylation, likely as a result of the increased expression. STAT3 is considered to be oncogenic in the prostate [51]. For example, a higher presence of phosphorylated STAT3 was detected in samples from PCa patients, and *STAT3* knockdown led to increased apoptosis in DU145 cells [52,53]. In contrast to LNCaP, STAT3 could not be detected in PC-3 cells, and AKT phosphorylation was not affected after *DHRS7* knockdown, but, surprisingly, ERK phosphorylation was abolished. Whether this is a result of increased negative feedback requires further investigations [54]. Although DU145 cells showed increased STAT3 expression and a weak trend of increased phosphorylation after *DHRS7* knockdown, it appears that, in these cells, the PI3K/AKT pathway is the preferred signaling cascade, as indicated by the induced phosphorylation of both residues S473 and T308 on AKT. PTEN is absent in both LNCaP and PC-3 cells, whereas it is functionally expressed in DU145 cells [54,55,56]. Furthermore, lower basal AKT phosphorylation was reported in DU145 compared with LNCaP and PC-3 cells [55,57], a trend that is largely consistent with our findings. Whether *DHRS7* knockdown mediated these effects via EGFR, or whether STAT3, AKT, and ERK might be affected by other pathways influenced by DHRS7, requires further research focusing on these signaling pathways by using appropriate inhibitors to distinguish between EGFR-dependent and -independent effects following *DHRS7* knockdown.

The inverse correlation between DHRS7 and EGFR expression and the increase in EGFR expression and phosphorylation following *DHRS7* knockdown could be of therapeutic relevance as the loss of DHRS7 may alter the sensitivity of the cancer cells towards antibodies and inhibitors of EGFR. Interestingly, EGF treatment led to reduced total EGFR expression in both knockdown and control cells, most likely due to receptor internalization followed by degradation upon ligand stimulation [58].

Interestingly, retrospective cohort studies found that men exposed to the mineralocorticoid receptor (MR)-antagonist spironolactone had a lower PCa risk [59,60], suggesting the tumor-promoting role of the MR. This receptor is stimulated by the steroids aldosterone and cortisol [61]. The stimulation of ectopically expressed MR by aldosterone in Chinese hamster ovary cells led to an induction of EGFR expression, which could be inhibited by spironolactone [62]. Similarly, HEK cells transfected with MR showed the increased activity of an ectopically expressed EGFR promoter construct when treated with aldosterone [63]. Furthermore, stimulation by EGF leads to the activation of the downstream mediator ERK [62]. Adrenalectomized rats under aldosterone treatment showed enhanced EGFR expression in kidney cortex homogenate as compared to animals without aldosterone treatment [62]. A previous in vitro study showed the potential of DHRS7 to catalyze the 20-oxoreduction of corticosteroids [15], thereby leading to inactive or less-active metabolites. Whether expression of EGFR via MR stimulation is of relevance in disease, and whether DHRS7 is able to prevent MR-mediated EGFR expression under physiological circumstances, remain to be addressed.

The ligand-binding pocket of DHRS7 is highly lipophilic. This can be inferred from the finding that DHRS7 accepts steroids and all-*trans* retinal as substrates [11,14]. However, substrates other than the already-identified ones are likely to be involved in the observed effects in PCa cells. An interesting group of potential lipophilic substrates represents the prostaglandins. An increase in cell proliferation, adhesion, and migration was observed in PGE_2_-treated PC-3 cells [64]. Furthermore, PGE_2_ treatment of normal gastric epithelial and colon cancer cells led to an activation of EGFR and ERK [65]. Another group of substrates of potential interest includes arachidonic-acid-derived metabolites. Interactome profiling of the cannabinoid receptor 2 (CB2) expressed in HEK-293 identified DHRS7 as an interactor that was detected only in CB2-expressing cells [66]. Ligands of CB2 are lipophilic compounds, e.g., 2-arachidonoyl glycerol [67], and increased DHRS7 expression in CB2-expressing cells might indicate a role in the synthesis or metabolism of lipid derivatives related to CB2 signaling. The use of lipidomics, with a focus on certain subgroups of compounds (e.g., retinoids, fatty acids, or prostaglandins) could be a valuable strategy for the discovery of the physiological substrates of DHRS7.

## 5. Conclusions

The results of the present study emphasize that, despite the ability of DHRS7 to metabolize 5α-DHT, the effects of this enzyme in PCa cells are androgen-independent. Using proteomics, we identified a time-dependent increase in EGFR expression in LNCaP cells following the siRNA-mediated *DHRS7* knockdown. This observation was confirmed using RT-qPCR and Western blotting in LNCaP cells and the two AR-negative, PCa-derived cell lines, PC-3 and DU145. The three PCa cell lines to which it was applied showed an inverse correlation between DHRS7 and EGFR expression, which was further supported by an increase in DHRS7 expression after *EGFR* knockdown. Knockdown of *DHRS7,* followed by treatment with EGF, led to increased receptor phosphorylation. Importantly, we found an inverse correlation, both on the mRNA and the protein level, for the expression of DHRS7 and EGFR in samples from PCa patients. DHRS7 expression negatively correlated with Gleason pattern and positively with the overall and disease-free survival rates of PCa patients, and EGFR showed the opposite behavior. This study clearly points to the role of DHRS7 as a tumor-suppressor in PCa and should stimulate further work to more profoundly understand this fundamental link and elucidate the tumor-suppressor role of DHRS7 in PCa. More research is also needed to elucidate the exact molecular mechanism underlying the link between DHRS7 and EGFR, to assess whether the inverse correlation of DHRS7 and EGFR expression can be replicated in metastatic PCa samples, to test whether DHRS7 expression might help to discriminate Gleason score- and age-matched patients with different prognosis, and to identify the physiological DHRS7 substrate(s).

## Data Availability

All relevant data are included within the manuscript and in its supporting information files. All related study data will be provided after publication, according to the related data-management plan, as open access at https://zenodo.org/. The mass spectrometry proteomics data have been deposited with the ProteomeXchange Consortium via the PRIDE partner repository (https://www.ebi.ac.uk/pride/) with the dataset identifier PXD031229 [68].

## Supplementary Materials

The following are available online at https://www.mdpi.com/article/10.3390/cancers14133074/s1: Figure S1: Heat maps of the proteomics data after knockdown of *DHRS7* in LNCaP cells. Figure S2: Expression of AR and its target genes *KLK3*, *TMPRSS2,* and *FKBP5* in LNCaP cells after *DHRS7* knockdown. Figure S3: Proteins with the KEGG annotation “prostate cancer”. Figure S4: Effect of DHRS7 knockdown on ERK and AKT and their phosphorylation in LNCaP, PC-3, and DU145 cells treated with EGF. Figure S5: Expression of ERK and AKT in LNCaP, PC-3, and DU145 cells. Figure S6: Correlation of the *TCGA* data of *STAT3* with *DHRS7* and *EGFR*. Figure S7: Age of patients within the low- and high-expression groups of DHRS7 and EGFR of the overall survival analysis. Table S1: siRNA sequences used for knockdown experiments. Table S2: Primers used for RT-qPCR. Table S3: Antibodies used for Western blotting. Table S4: Distribution of the Gleason scores in percentages among the low- and high-expression groups of DHRS7 and EGFR. File S1: Proteomics data after 24 h. File S2: Proteomics data after 36 h. File S3: Proteomics data after 48 h. File S4: Annotation of proteomics data. File S5: Western blots.
