# Supplementary material for: The Potential Tumor-Suppressor DHRS7 Inversely Correlates with EGFR Expression in Prostate Cancer Cells and Tumor Samples
Source: Cancers (Basel). 2022 Jun 23;14(13):3074. doi: 10.3390/cancers14133074 (PMC9264982; doi:10.3390/cancers14133074)
Supplement: Supplementary file 1 [file cancers-14-03074-s001.zip › cancers-1775926_R1-supplementary_final.pdf]

The Potential Tumor Suppressor DHRS7 Inversely Correlates with EGFR Expression in Prostate Cancer Cells and Tumor Samples

Simon Stücheli [1], Selene Araya [1], Caner Ercan [2,3], Seraina O. Moser [1], John Gallon [3], Paul Jenö [4], Salvatore Piscuoglio [2,3], Luigi Terracciano [2], and Alex Odermatt [1,*]

Table S1. siRNA sequences used for knockdown experiments

Target Gene	Sequence (5′ – 3′)	Cat#
DHRS7	GAA AGA AGU UUG GAU CUC A	D-009573-02
EGFR siRNA-1	CCG CAA AUU CCG AGA CGA A	D-003114-32
EGFR siRNA-2	CAA AGU GUG UAA CGG AAU A	D-003114-33
EGFR siRNA-3	GUA ACA AGC UCA CGC AGU U	D-003114-34
EGFR siRNA-4	GAG GAA AUA UGU ACU ACG A	D-003114-35
Non-targeting control	UGG UUU ACA UGU UUU CUG A	D-001810-03-05

Table S2. Primers used for RT-qPCR

Target Gene	Forward (5′ – 3′)	Reverse (5′ – 3′)
AR	GAC ATG CGT TTG GAG ACT GC	TTC CCT TCA GCG GCT CTT TT
DHRS7	GAG TTT GGT AGA ATC GAC ATT GTG	GAA AGA GGT ACA GAT ATG ATA CCC
EGFR	GGC AGG AGT CAT GGG AGA A	GCG ATG GAC GGG ATC TTA G
FKBP5	GAA TAC ACC AAA GCT GT	CTC TTC CTT GGC ATC CT
KLK3	AGG CCT TCC CTG TAC AC	GTC TTG GCC TGG TCA TTT CC
PPIA	CAT CTG CAC TGC CAA GAC TGA	TGC AAT CCA GCT AGG CAT G
STAT3	CAG CAG CTT GAC ACA CGG TA	AAA CAC CAA AGT GGC ATG TGA
TMPRSS2	CTG CCA AGG TGC TTC TC	TTA GCC GTC TGC CCT C
GAPDH	CAG CCT CAA GAT CAT CAG CA	TTC TAG ACG GCA GGT CAG GT

Table S3. Antibodies used for Western blotting

Target Protein	Cat#	Manufacturer	Clonality	RRID	Host species	Dilutions: Primary Antibody	Dilutions: Secondary Antibody
AKT	9272, Lot# 15	Cell Signaling Technology (Danvers, MA, USA)	Polyclonal	AB_329827	Rabbit	1:2000	1:5000
Phospho-AKT (S473)	4060, Lot# 19	Cell Signaling Technology	Monoclonal (D9E)	AB_2315049	Rabbit	1:2000	1:5000
Phospho-AKT (T308)	13038, Lot# 7	Cell Signaling Technology	Monoclonal (D25E6)	AB_2629447	Rabbit	1:2000	1:5000
AR	sc-7305, Lot# H3116	Santa Cruz Technology (Dallas, TX, USA)	Monoclonal (441)	AB_626671	Mouse	1:2000	1:5000
DHRS7	SAB1400567, Lot# J5091	Sigma-Aldrich	Polyclonal	AB_1847656	Mouse	1:2000	1:5000
EGFR	sc-373746, Lot# C2919	Santa Cruz Technology	Monoclonal (A-10)	AB_10920395	Mouse	1:1000	1:5000
Phospho-EGFR	sc-81488, Lot# C2019	Santa Cruz Technology	Monoclonal (15A2)	AB_1125777	Mouse	1:1000	1:5000
ERK	4695, Lot# 28	Cell Signaling Technology	Monoclonal (137F5)	AB_390779	Rabbit	1:2000	1:5000
Phospho-ERK	4370S, Lot# 28	Cell Signaling Technology	Monoclonal (D13.14.4E)	AB_2315112	Rabbit	1:1000	1:5000
FKBP5	sc-271547, Lot# C1918	Santa Cruz Technology	Monoclonal (D-4)	AB_10649040	Mouse	1:2000	1:2000
KLK3	sc-65602. Lot# A0913	Santa Cruz Technology	Monoclonal (SPM352)	AB_2134512	Mouse	1:75	1:5000
STAT3	sc-8019, Lot# A1719	Santa Cruz Technology	Monoclonal (F-2)	AB_628293	Mouse	1:2000	1:5000
Phospho-STAT3	sc-8059, Lot# L1619	Santa Cruz Technology	Monoclonal (B-7)	AB_628292	Mouse	1:2000	1:5000
TMPRSS2	sc-515727, Lot# H0916	Santa Cruz Technology	Monoclonal (H-4)	AB_2892118	Mouse	1:1000	1:5000
γ-Tubulin	T5192, Lot# 059M4807V	Sigma-Aldrich	Polyclonal	AB_261690	Rabbit	1:2000	1:5000
Mouse IgG	A0168, Lot# 079M4881V	Sigma-Aldrich	Polyclonal	AB_257867	Goat	-	-
Rabbit IgG	7074S, Lot# 22	Cell Signaling Technology	Polyclonal	AB_2099233	Goat	-	-

Table S4. Distribution of the Gleason Scores in percentage, among the low and high expression groups of DHRS7 and EGFR of the overall survival analysis.

Gleason Score	DHRS7 Expression		EGFR Expression	
	Low	High	Low	High
6	-	21 %	27 %	3 %
7	-	40 %	49 %	18 %
8	-	7 %	4 %	8 %
9	62 %	30 %	18 %	61 %
10	38 %	3 %	2 %	11 %

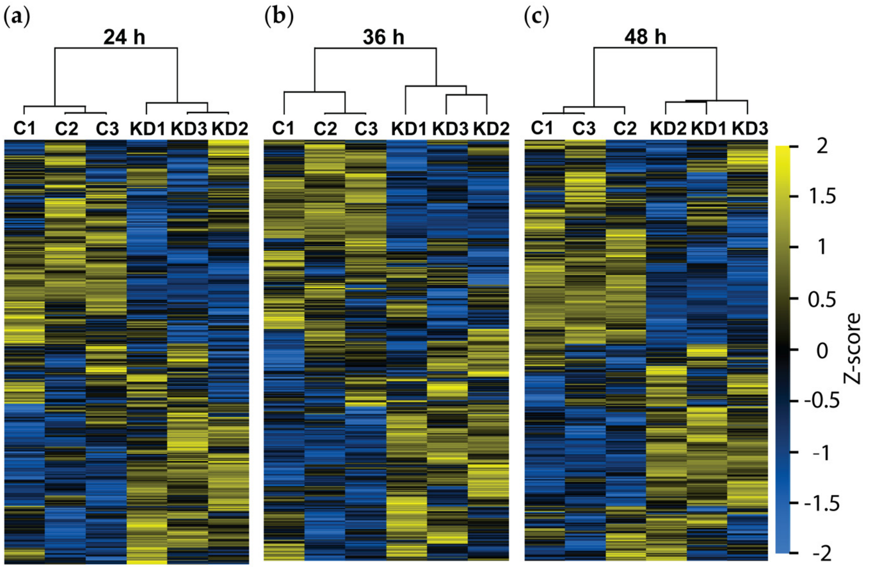

Figure S1. Heat maps of the proteomics data after knockdown of DHRS7 in LNCaP cells. Representation of protein changes following DHRS7 knockdown for (**a**) 24 h; (**b**) 36 h; and (**c**) 48 h. Expression levels were normalized using Z-score followed by hierarchical clustering with the Perseus software. Each time point was measured in three technical replicates. C = non-targeting siRNA control; KD = knockdown of *DHRS7*. Yellow: upregulated proteins; blue: downregulated proteins.

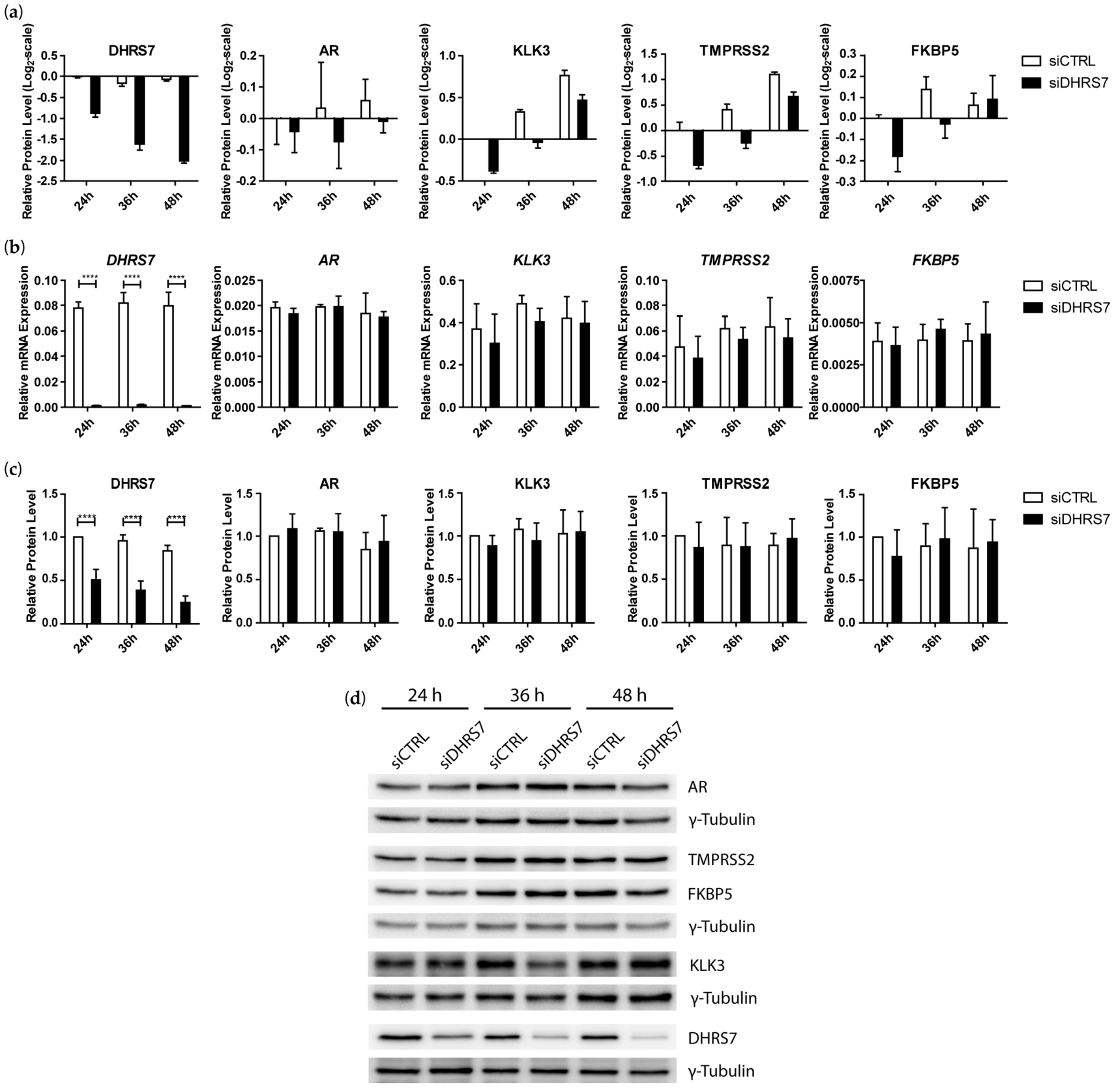

Figure S2. Expression of AR and its target genes *KLK3*, *TMPRSS2* and *FKBP5* in LNCaP cells after *DHRS7* knockdown. *DHRS7* was knocked-down in LNCaP cells for 24 h, 36 h and 48 h. (**a**) Relative protein expression from the proteomics experiments. Control at 24 h was set to zero and all other data sets were compared to the control at 24 h. (**b**) mRNA expression levels relative to that of *PPIA*. (**c**) Densitometry analysis of protein expression levels relative to the control at 24 h and normalized to γ-Tubulin. (**d**) Representative Western Blot (one of three) with γ-Tubulin as a loading control. Data represent mean ± SD of the proteomics data (n = 1, technical triplicates, panel (**a**)) and mean ± SD of three independent experiments for mRNA and densitometry (n = 3, panel (**b**) and (**c**)). siCTRL = non-targeting siRNA control; siDHRS7 = siRNA against *DHRS7*. For mRNA and densitometry results statistical significance was calculated using two-way ANOVA with Bonferroni multiple comparison post-test. **** $p < 0.0001$.

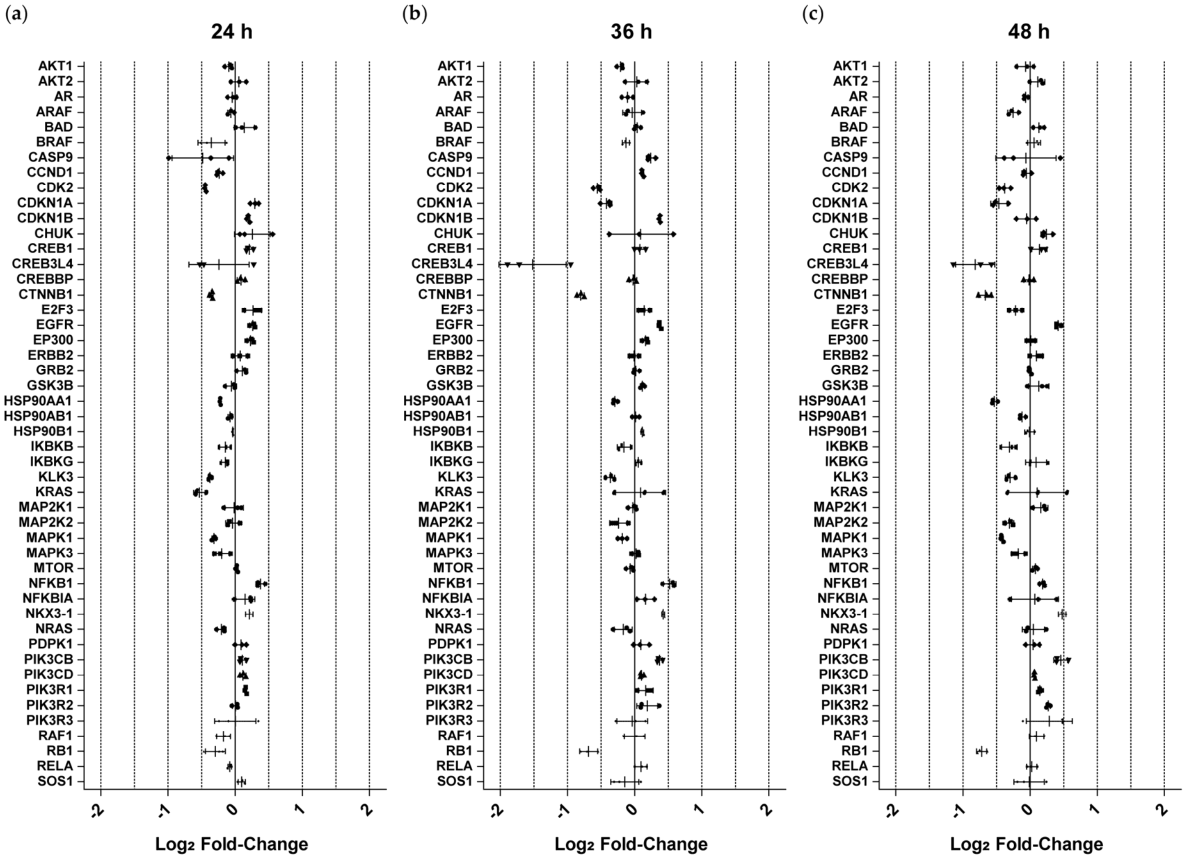

Figure S3. Proteins with the KEGG annotation "Prostate cancer". Knockdown of *DHRS7* in LNCaP cells was performed for (**a**) 24 h; (**b**) 36 h; and (**c**) 48 h. Expression levels are displayed as $\log_2$ fold-change (*DHRS7* knockdown compared to non-targeting siRNA control). Data points indicate technical triplicates with their mean ± SD (n = 1).

(a)

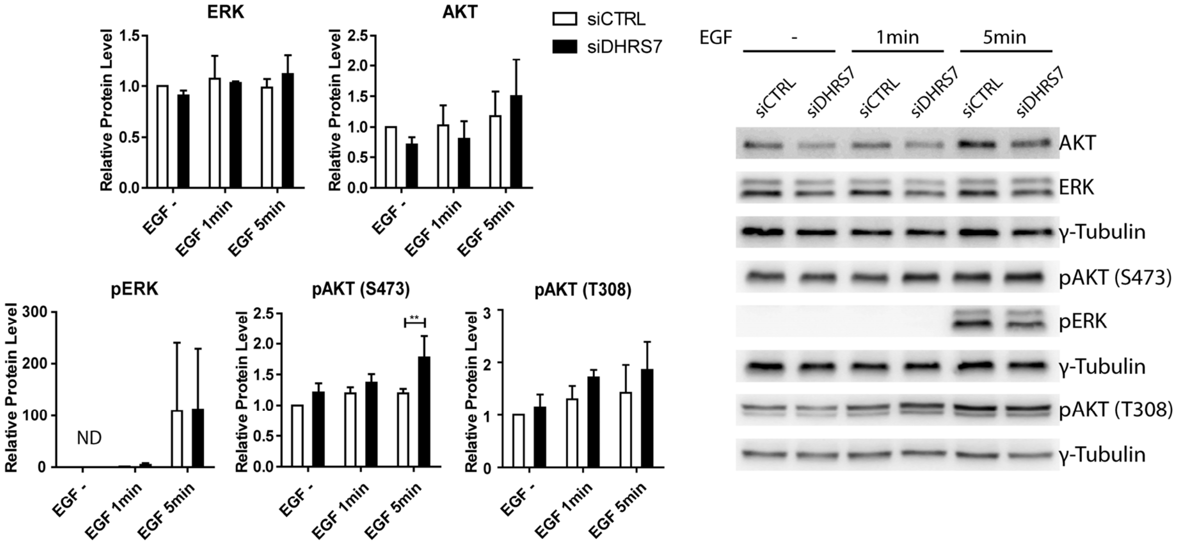
ERK
AKT
pERK
pAKT (S473)
pAKT (T308)
Relative Protein Level
ND
**
EGF -
EGF 1min
EGF 5min
siCTRL
siDHRS7
EGF
-
1min
5min
siCTRL
siDHRS7
AKT
ERK
γ-Tubulin
pAKT (S473)
pERK
pAKT (T308)

(b)

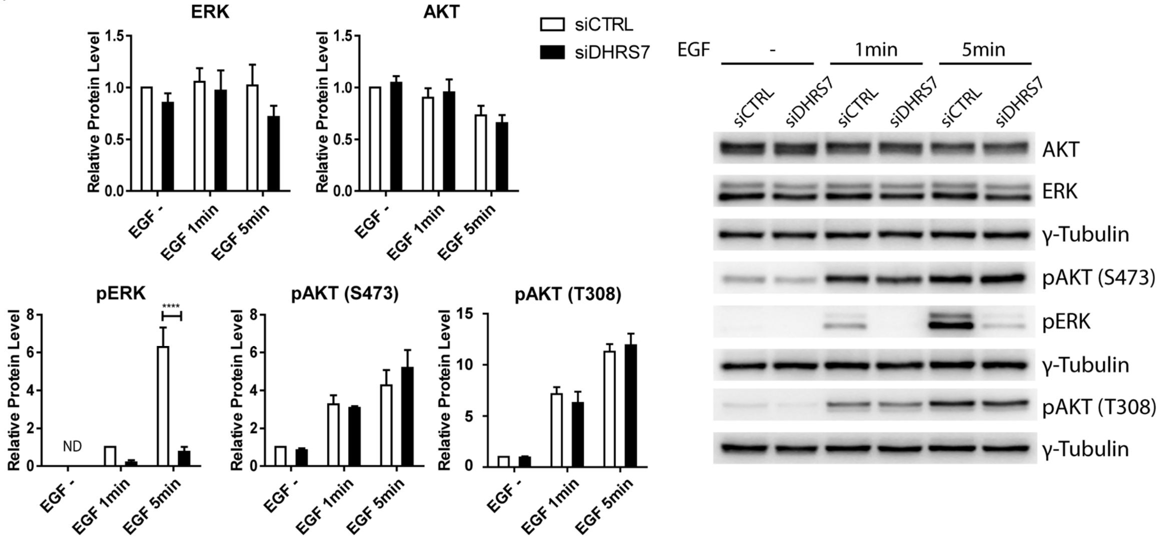
ERK
AKT
pERK
pAKT (S473)
pAKT (T308)
Relative Protein Level
ND
****
EGF -
EGF 1min
EGF 5min
siCTRL
siDHRS7
EGF
-
1min
5min
siCTRL
siDHRS7
AKT
ERK
γ-Tubulin
pAKT (S473)
pERK
pAKT (T308)

(c)

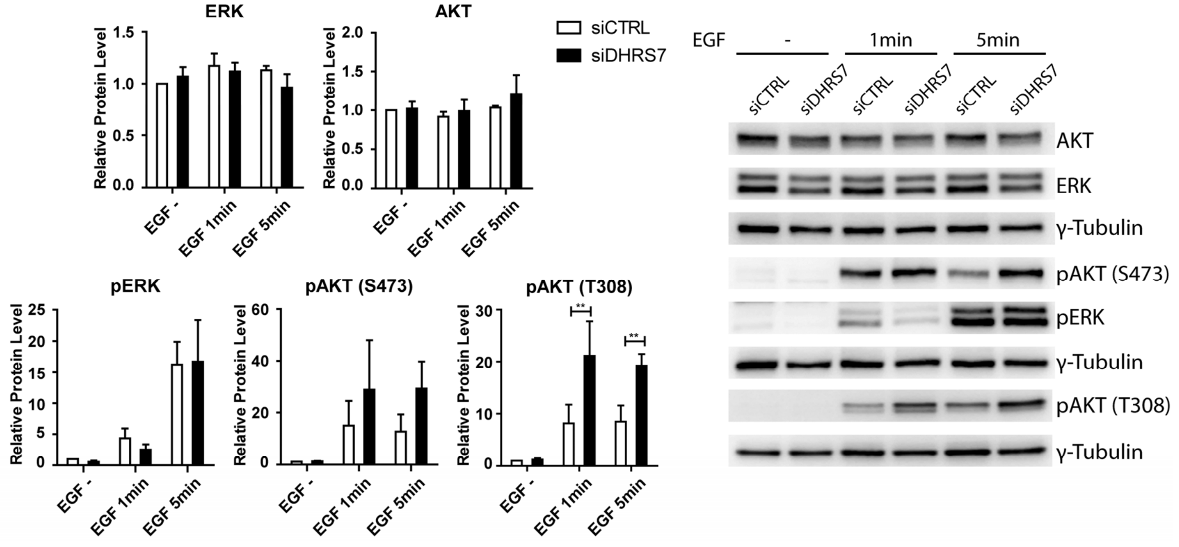
ERK
AKT
pERK
pAKT (S473)
pAKT (T308)
Relative Protein Level
**
EGF -
EGF 1min
EGF 5min
siCTRL
siDHRS7
EGF
-
1min
5min
siCTRL
siDHRS7
AKT
ERK
γ-Tubulin
pAKT (S473)
pERK
pAKT (T308)

Figure S4. Effect of *DHRS7* knockdown on ERK and AKT and their phosphorylation in (**a**) LNCaP, (**b**) PC-3, and (**c**) DU145 cells treated with EGF. Densitometry analysis of protein expression and phosphorylation levels are relative to control without treatment, or after 1 min of treatment where appropriate, and normalized to the γ-Tubulin loading control. Representative Western blots (one of three) are shown for each cell line. Data represent mean ± SD of three independent experiments (n = 3). CTRL = non-targeting siRNA control; KD = siRNA against *DHRS7*. Statistical significance was calculated using two-way ANOVA with Bonferroni multiple comparison post-test. ** $p < 0.01$; **** $p < 0.0001$.

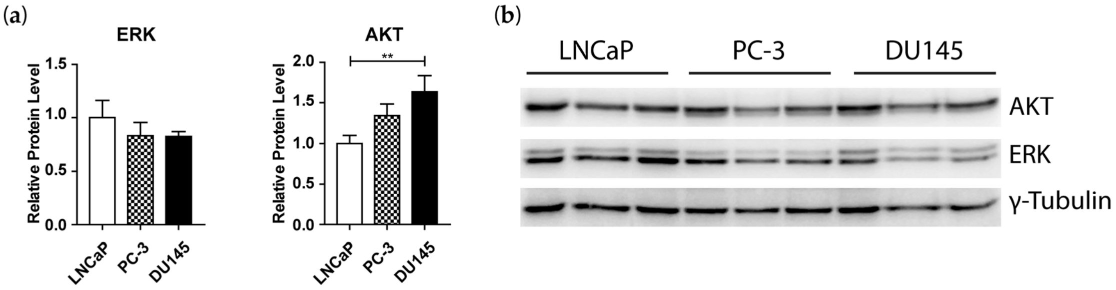

Figure S5. Expression of ERK and AKT in LNCaP, PC-3, and DU145 cells. (**a**) Densitometry analysis of protein expression levels normalized to the levels in LNCaP cells and the γ-Tubulin loading control. (**b**) Western blot of three individual samples per cell line with γ-Tubulin as a loading control. Data represent mean ± SD of three independent samples (n = 3). Statistical significance was calculated using one-way ANOVA with Bonferroni multiple comparison post-test. ** $p < 0.01$

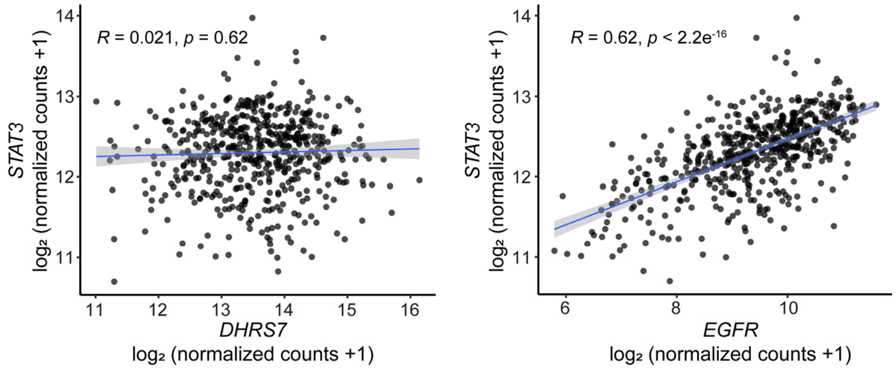

Figure S6. Correlation of the TCGA data of *STAT3* with *DHRS7* and *EGFR*, respectively, in PCa. The correlation was assessed using Spearman's correlation. n = 550 each.

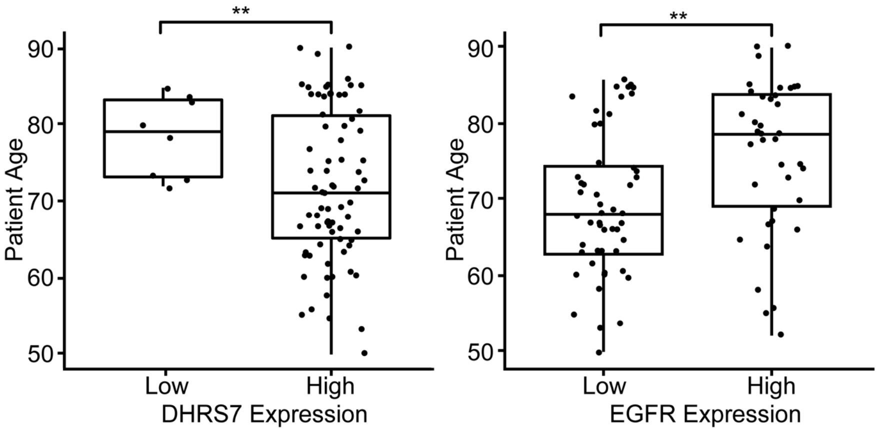

Figure S7. Age of patients within the low and high expression groups of DHRS7 and EGFR of the overall survival analysis. Statistical significance was calculated using a two-tailed t-test. ** $p < 0.01$
