# Supplementary material for: The Potential Tumor-Suppressor DHRS7 Inversely Correlates with EGFR Expression in Prostate Cancer Cells and Tumor Samples
Source: Cancers (Basel). 2022 Jun 23;14(13):3074. doi: 10.3390/cancers14133074 (PMC9264982; doi:10.3390/cancers14133074)

Western blots related to Figure 2

Lanes from left to right: Panel (d): siCTRL 24h, siDHRS7 24h, siCTRL 36h, siDHRS7 36h, siCTRL 48h, siDHRS7 48h.

EGFR and γ-Tubulin #1

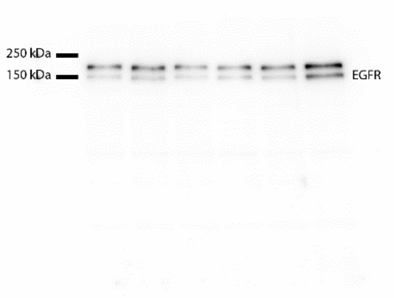

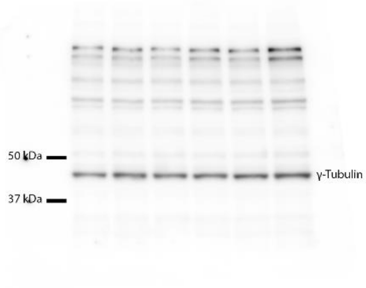

EGFR and γ-Tubulin #2

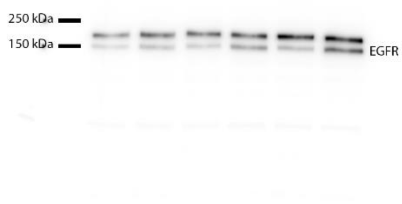

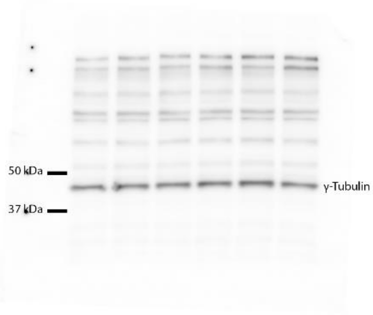

EGFR and γ-Tubulin #3

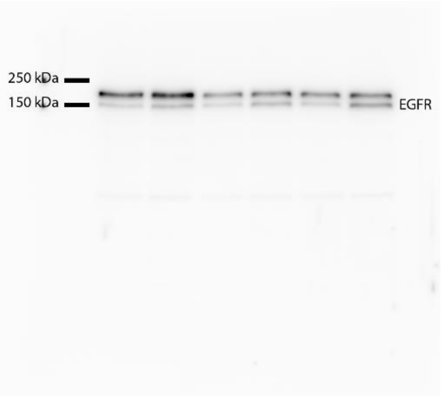

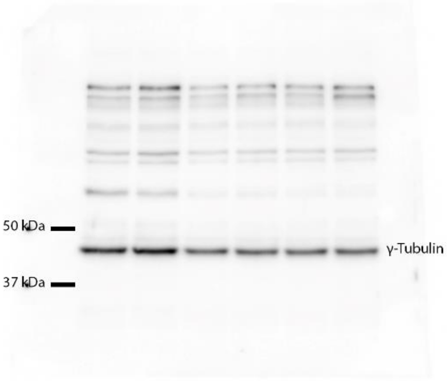

DHRS7, STAT3, γ-Tubulin #1

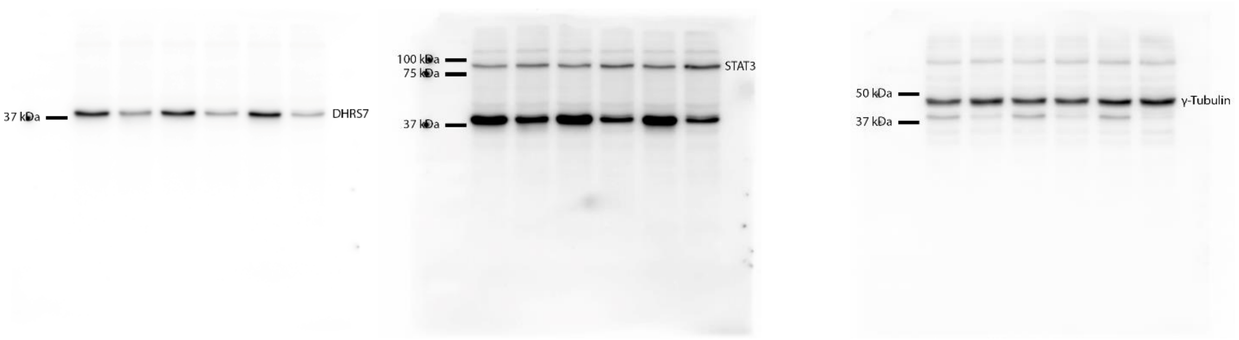

DHRS7. STAT3, γ-Tubulin #2

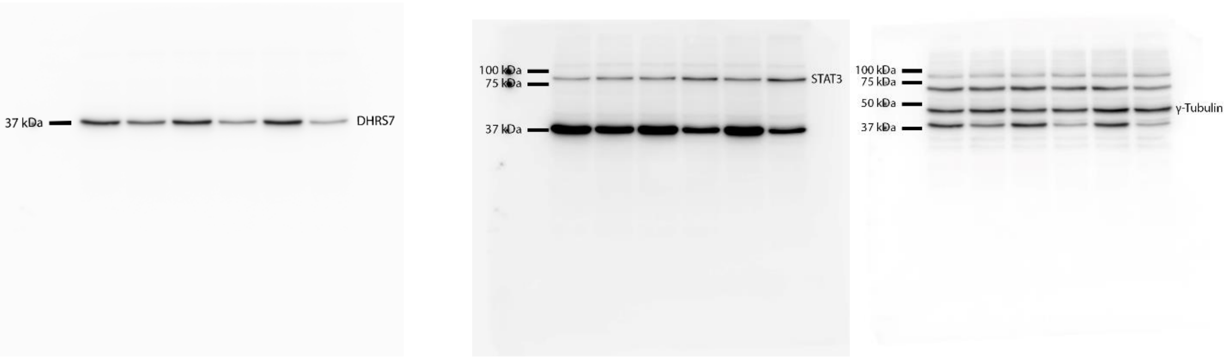

DHRS7, STAT3, γ-Tubulin #3

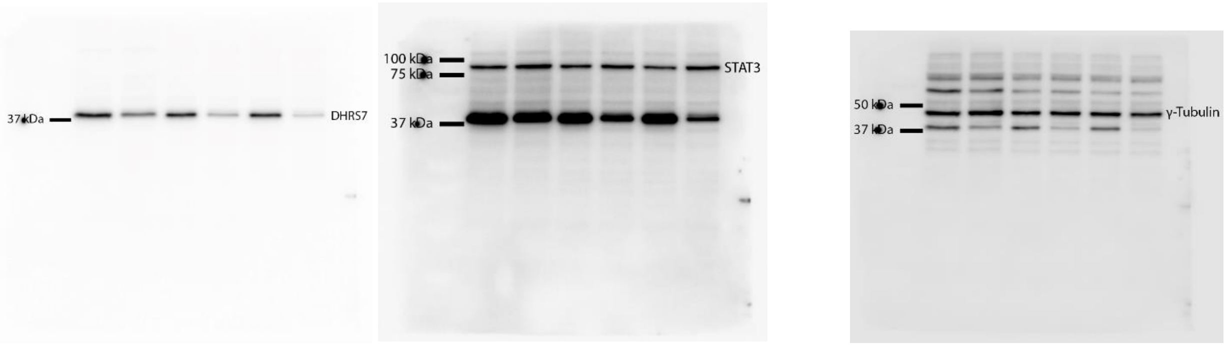

Lanes from left to right: Panel (e): Control, siRNA-1, siRNA-2, siRNA-3, siRNA-4

EGFR and γ-Tubulin

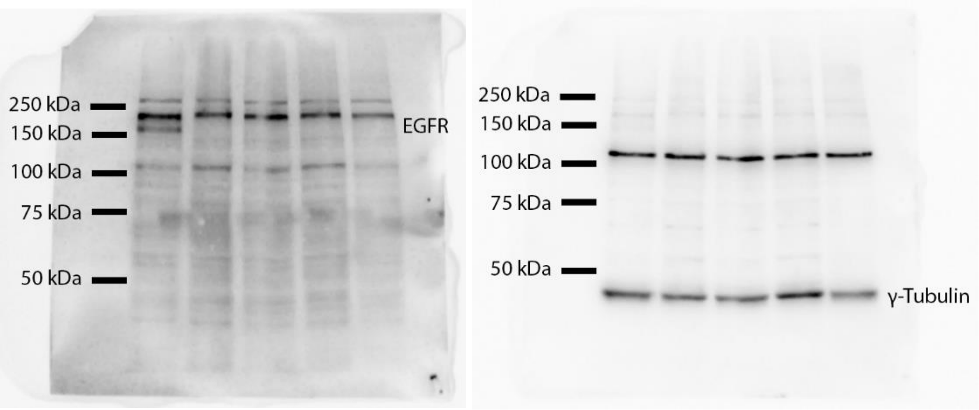

<u>Western blots related to Figure 3 and Supplementary Figure 5</u>

Lanes from left to right: LNCaP samples 1-3, PC-3 samples 1-3, DU145 samples 1-3.

EGFR, AR and γ-Tubulin

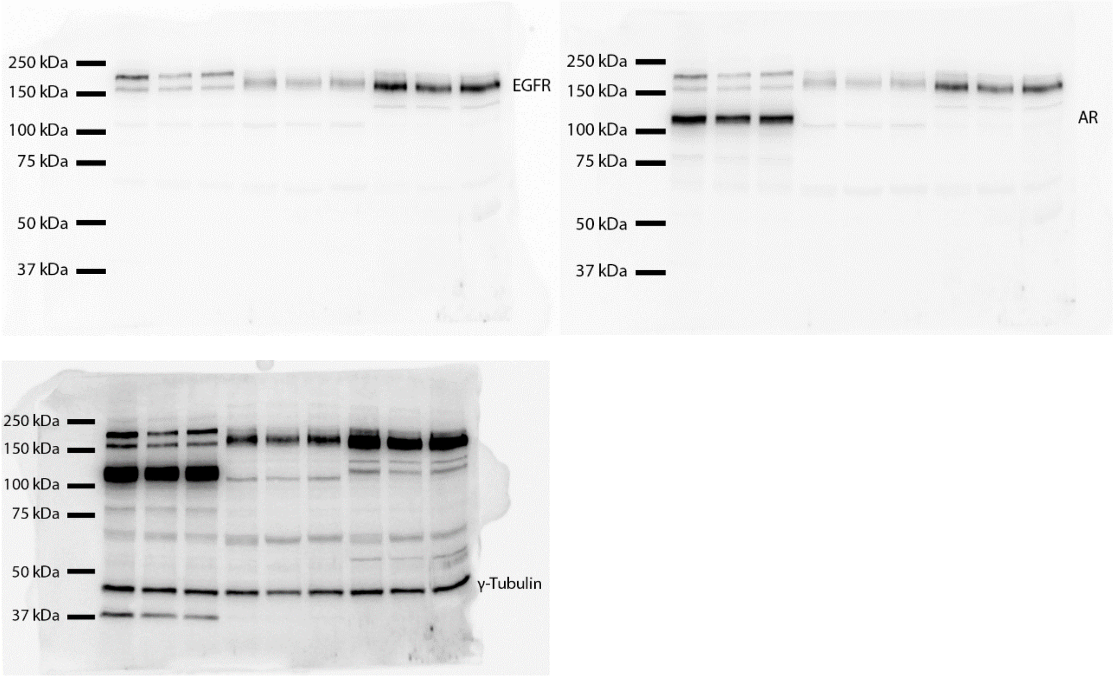

DHRS7 and γ-Tubulin

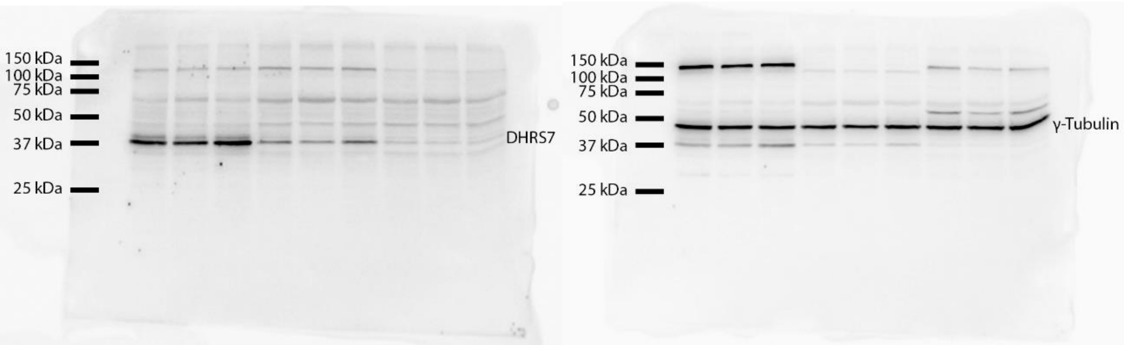

STAT3, AKT, γ-Tubulin and ERK

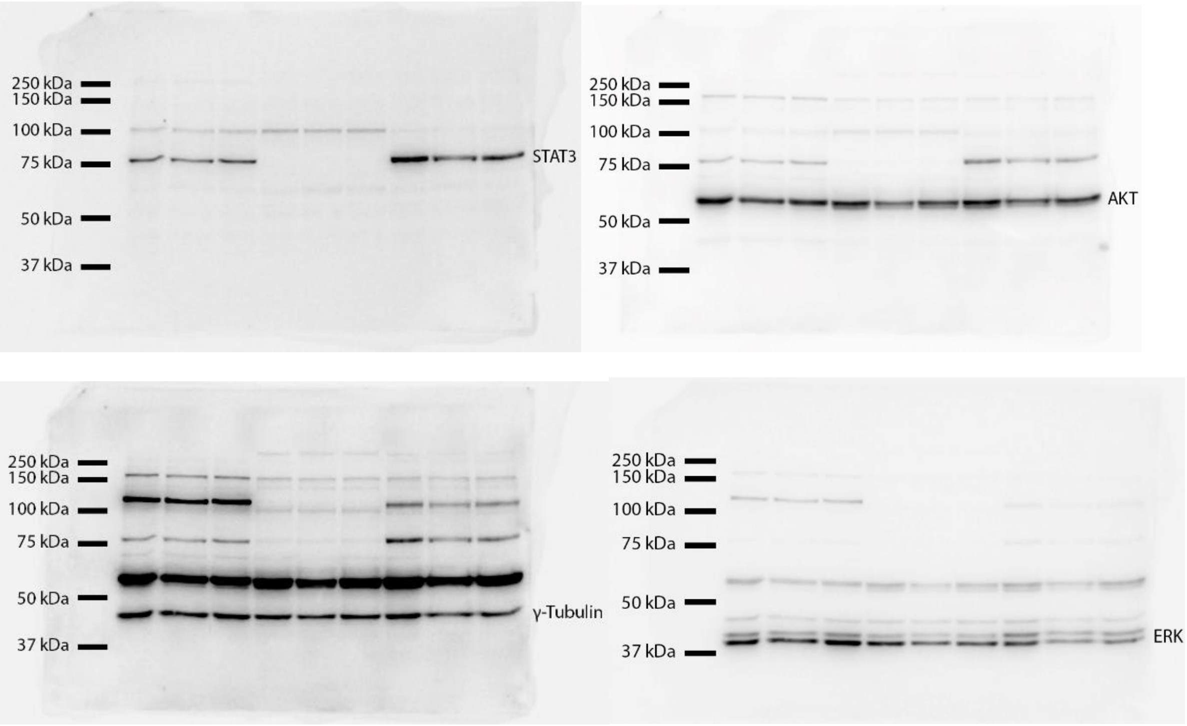

<u>Western blots related to Figure 4</u>

Lanes from left to right: PC-3 siCTRL#1 and siDHRS7#1, PC-3 siCTRL#2 and siDHRS7#2, PC-3 siCTRL#3 and siDHRS7#3

EGFR and γ-Tubulin

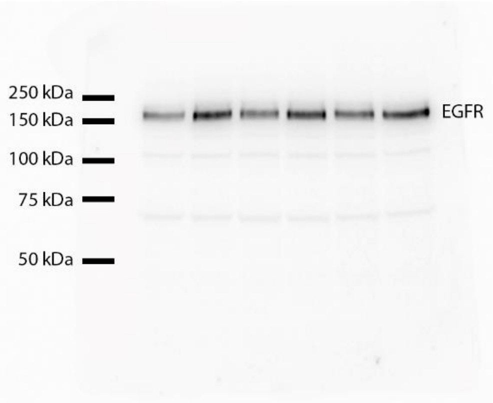

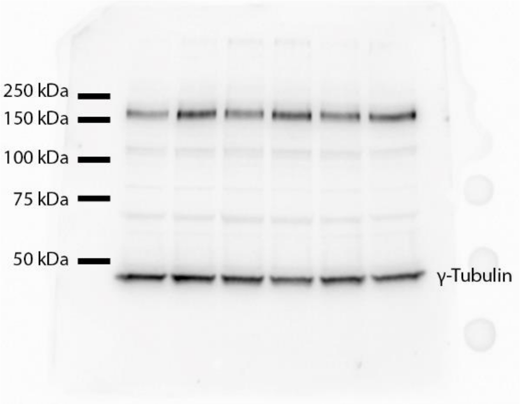

DHRS7 and γ-Tubulin

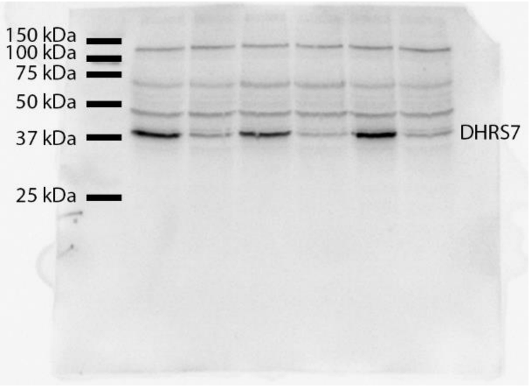

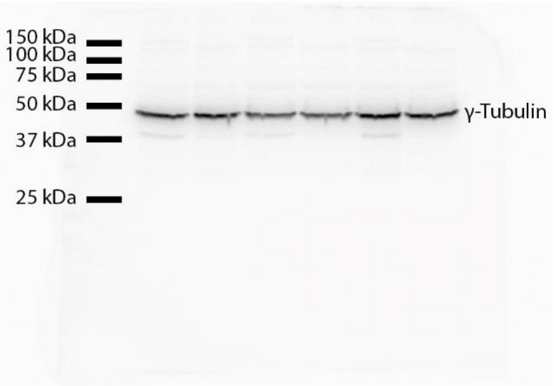

Lanes from left to right: DU145 siCTRL#1 and siDHRS7#1, DU145 siCTRL#2 and siDHRS7#2, DU145 siCTRL#3 and siDHRS7#3

EGFR and γ-Tubulin

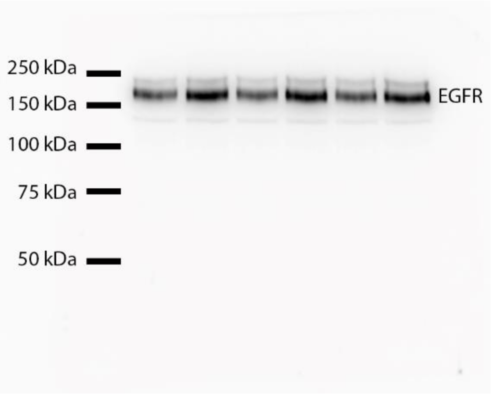

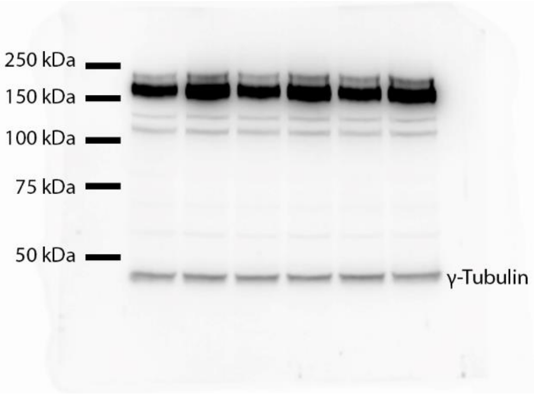

STAT3 and γ-Tubulin

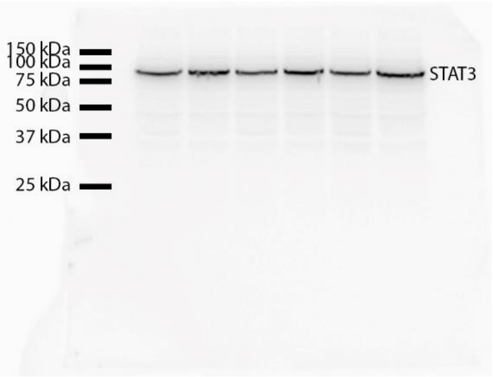

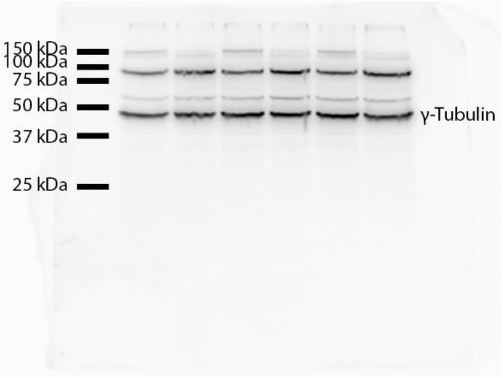

DHRS7 and γ-Tubulin

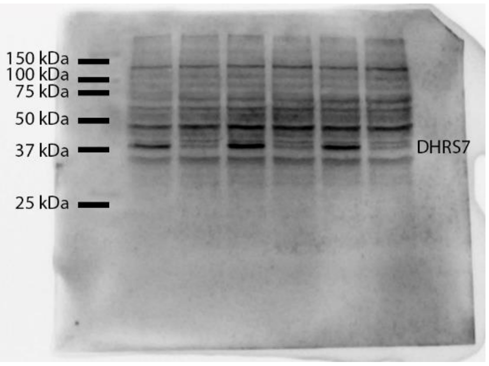

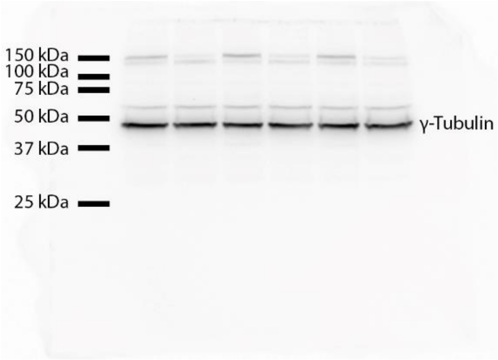

<u>Western blots related to Figure 5</u>

Lanes from left to right: LNCaP siCTRL#1 and siEGFR#1, LNCaP siCTRL#2 and siEGFR#2, LNCaP siCTRL#3 and siEGFR#3

STAT3, EGFR γ-Tubulin and AR

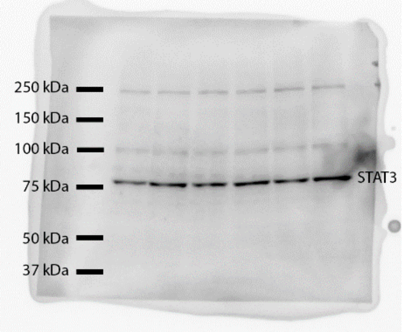

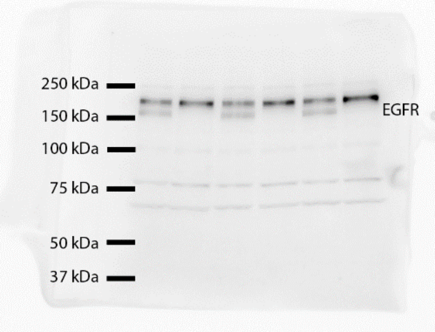

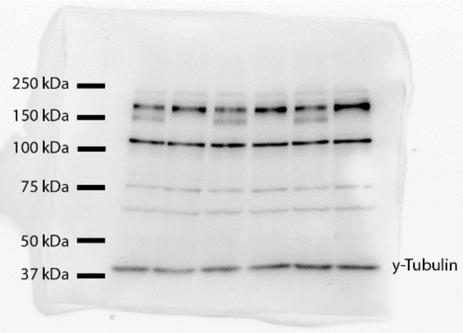

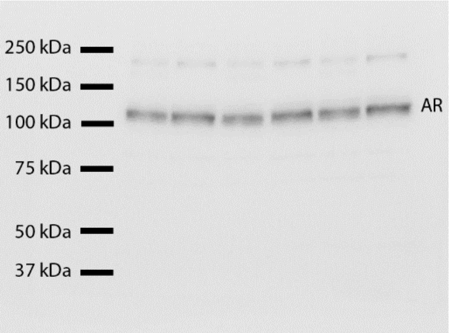

DHRS7 and γ-Tubulin

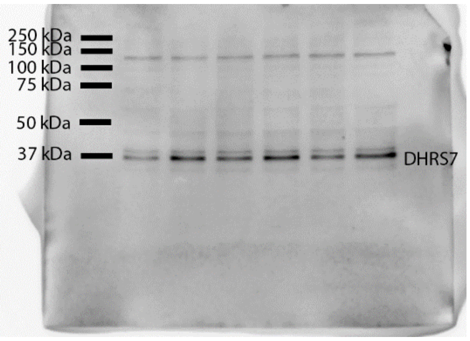

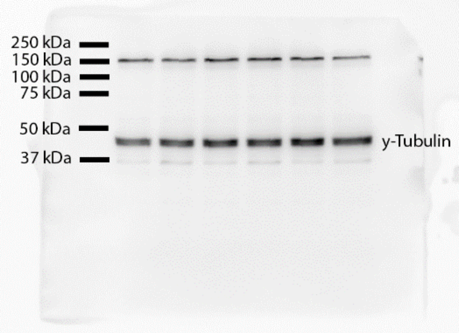

Lanes from left to right: PC-3 siCTRL#1 and siEGFR#1, PC-3 siCTRL#2 and siEGFR#2, PC-3 siCTRL#3 and siEGFR#3

EGFR and γ-Tubulin

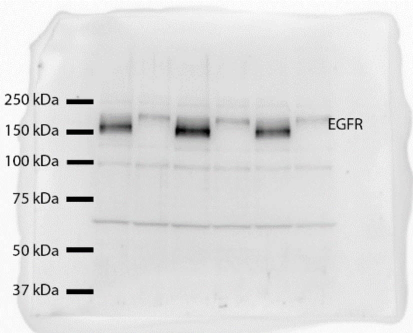

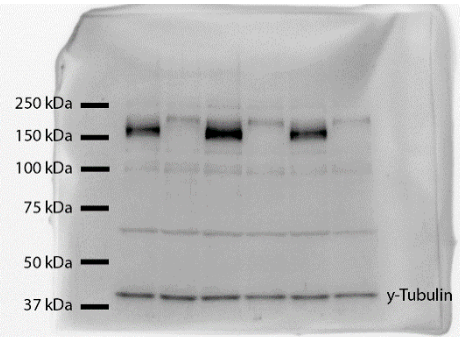

DHRS7 and γ-Tubulin

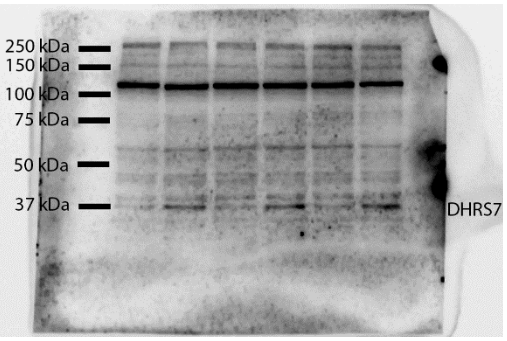

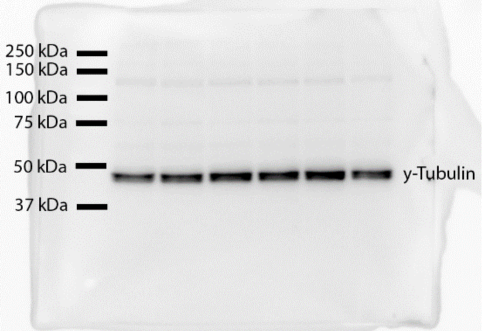

Lanes from left to right: DU145 siCTRL#1 and siEGFR#1, DU145 siCTRL#2 and siEGFR#2, DU145 siCTRL#3 and siEGFR#3

EGFR, STAT3 and γ-Tubulin

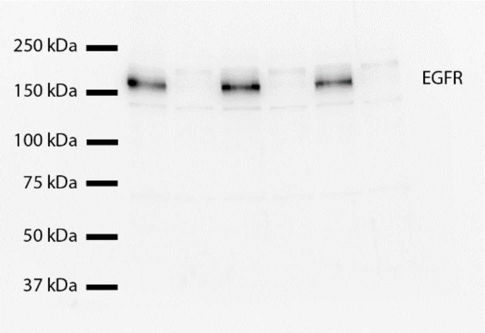

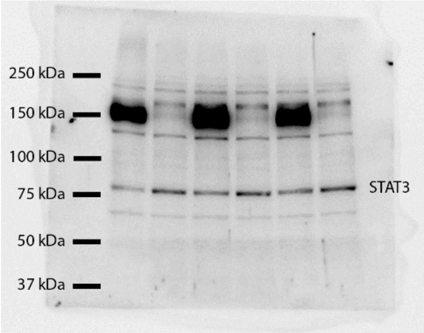

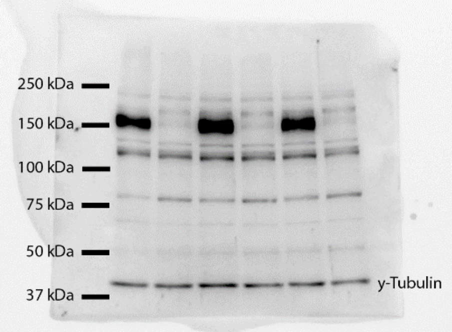

DHRS7 and γ-Tubulin

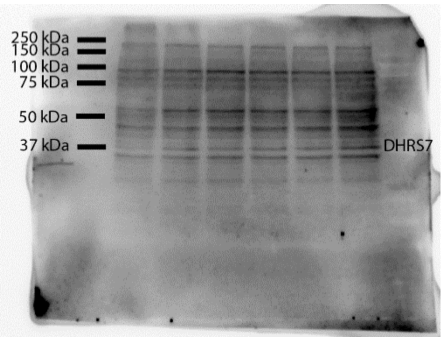

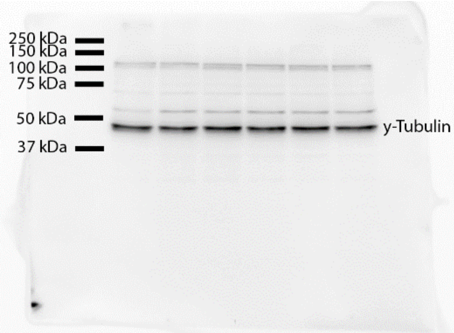

Western blots related to Figure 6 and Supplementary Figure 4: LNCaP samples

Lanes from left to right: siCTRL -, siDHRS7 -, siCTRL EGF 1min, siDHRS7 EGF 1min, siCTRL EGF 5min, siDHRS7 EGF 5min.

DHRS7 and γ-Tubulin #1

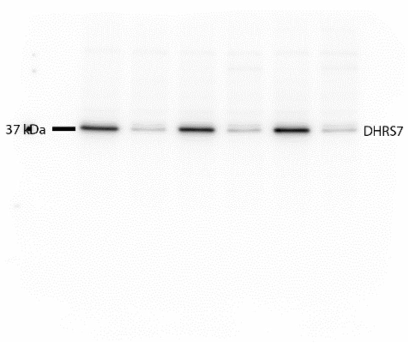

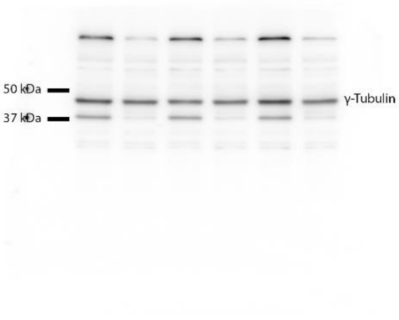

DHRS7 and γ-Tubulin #2

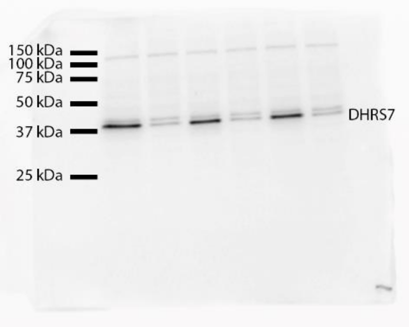

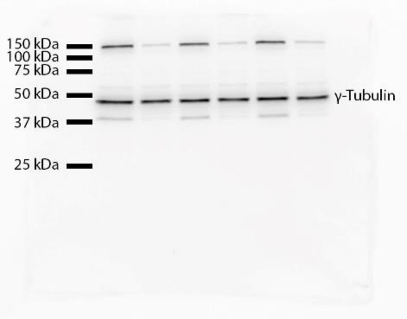

DHRS7 and γ-Tubulin #3

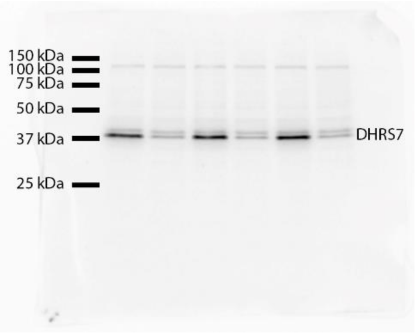

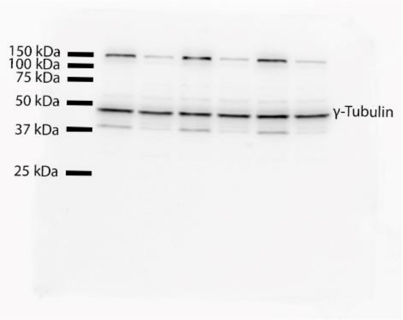

EGFR, STAT3, γ-Tubulin, ERK and AKT #1

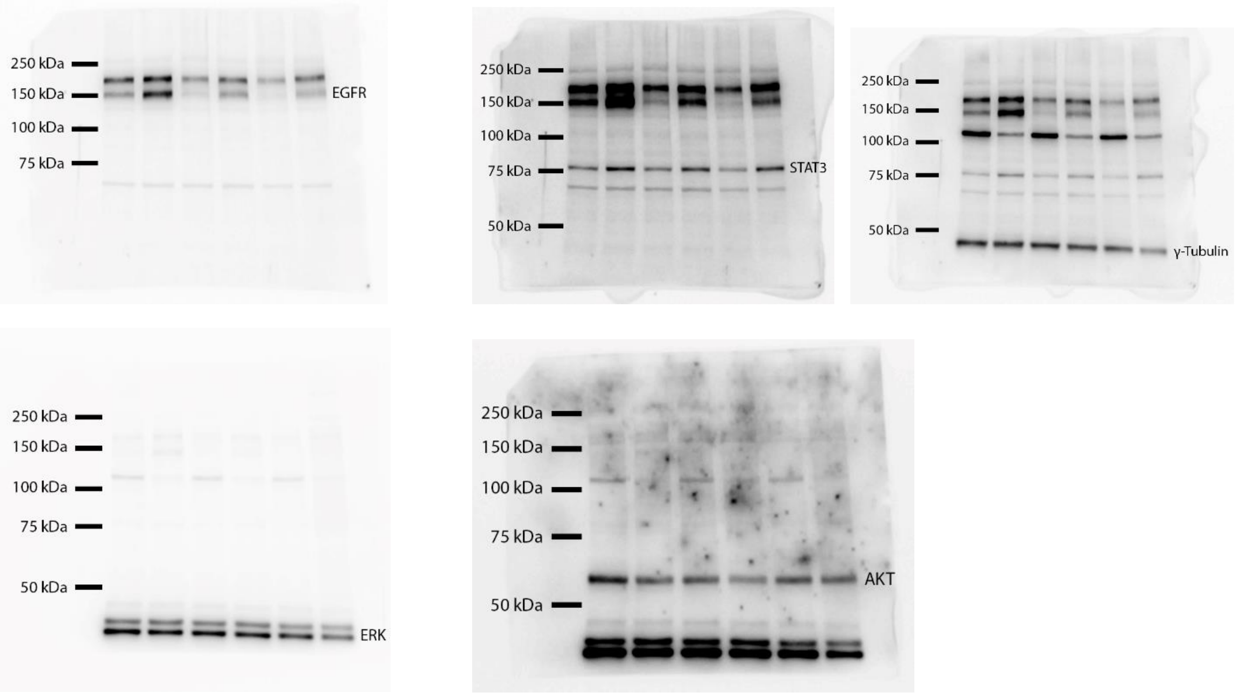

EGFR, STAT3, γ-Tubulin, ERK and AKT #2

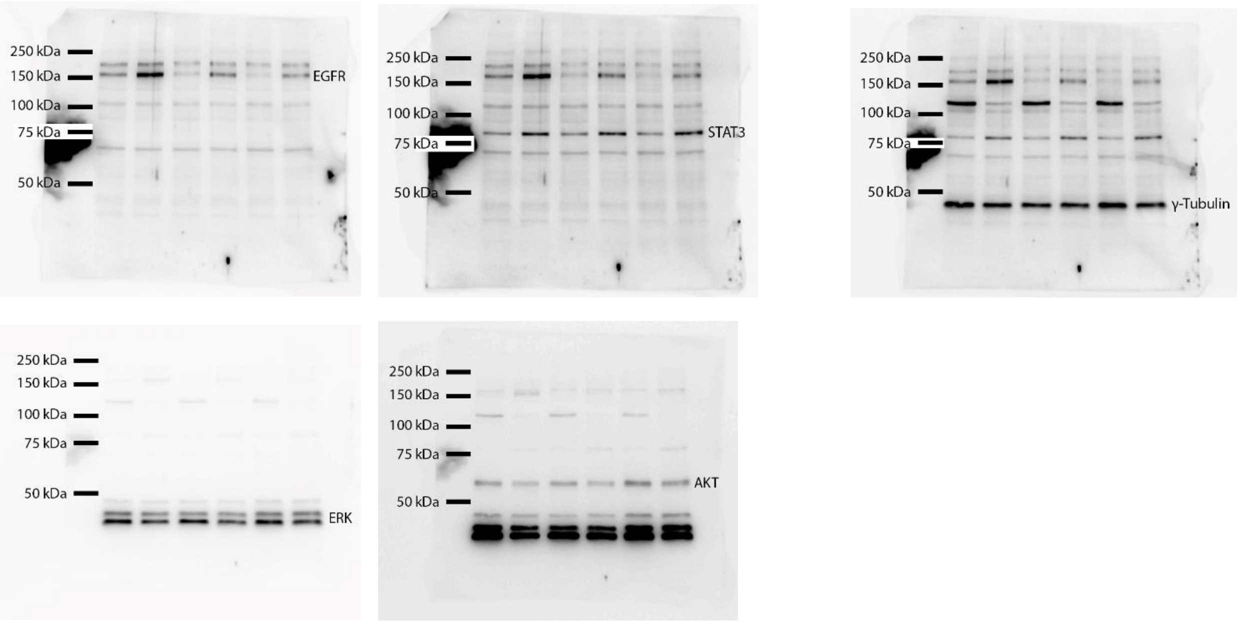

EGFR, STAT3, γ-Tubulin, ERK and AKT #3

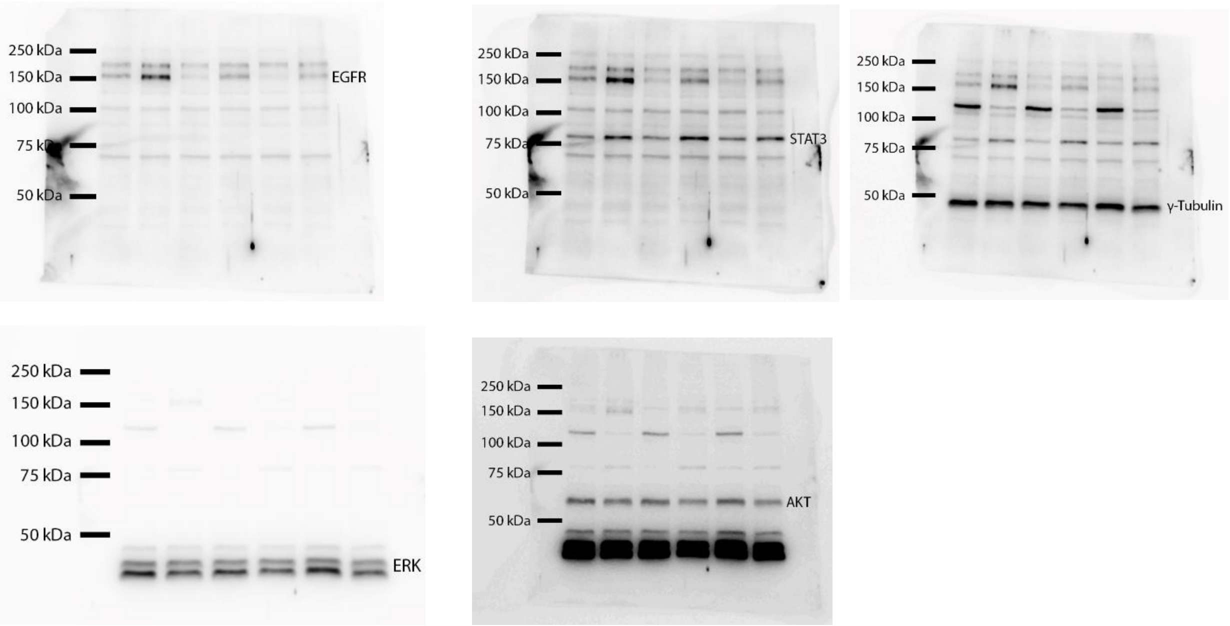

pEGFR, pSTAT3, γ-Tubulin, pERK and pAKT(S473) #1

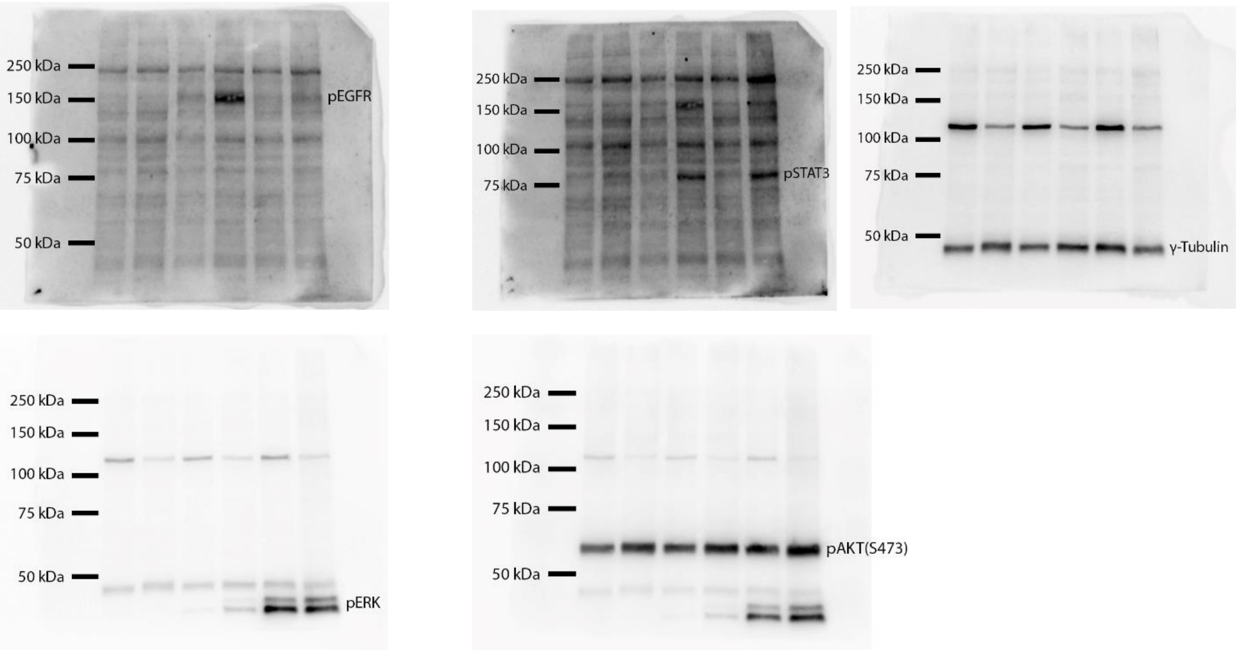

pAKT(T308) and y-Tubulin #1

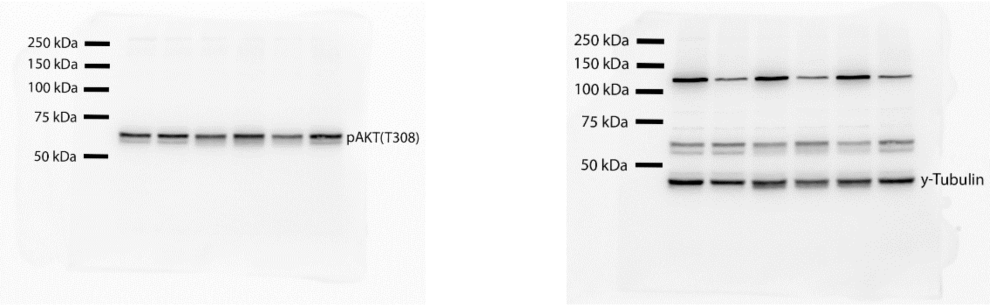

pEGFR, pSTAT3, γ-Tubulin, pERK and pAKT(S473) #2

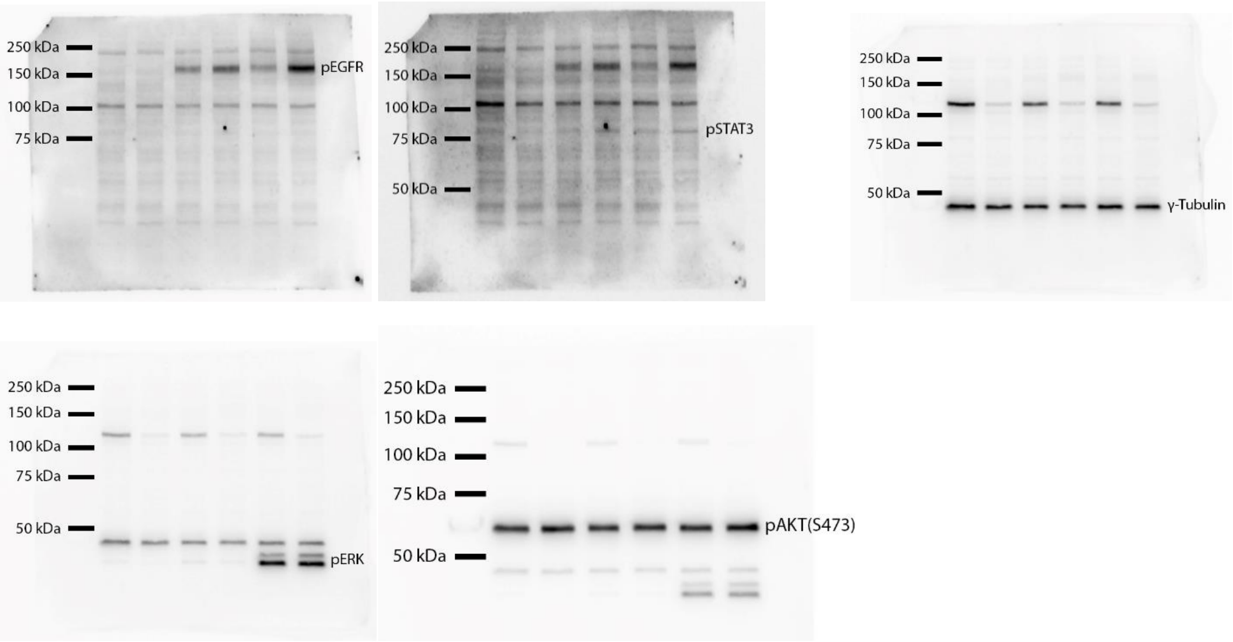

pAKT(T308) and y-Tubulin #2

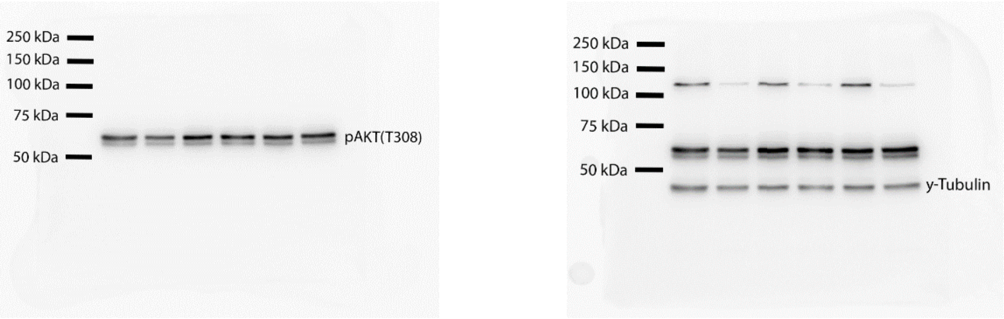

pEGFR, pSTAT3, γ-Tubulin, pERK and pAKT(S473) #3

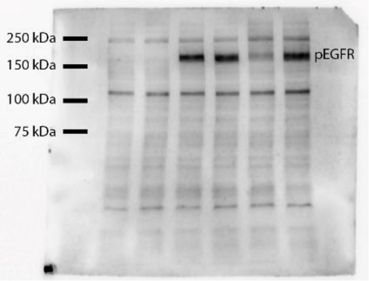

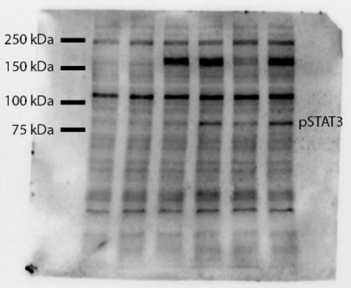

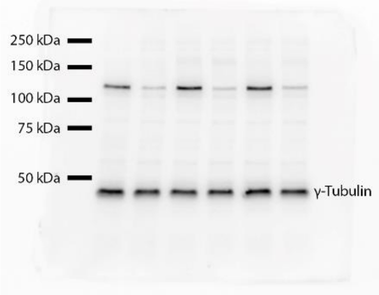

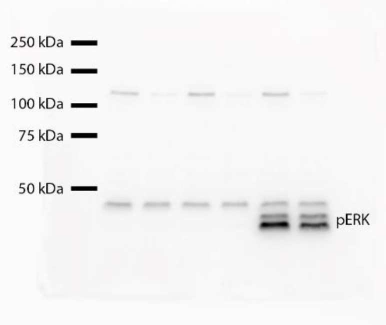

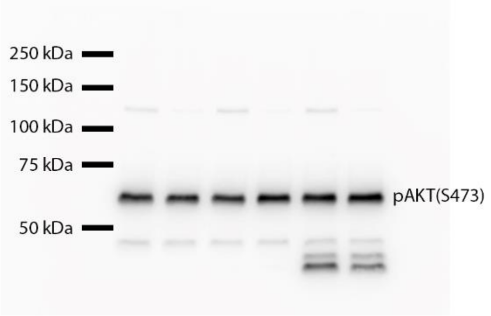

pAKT(T308) and y-Tubulin #3

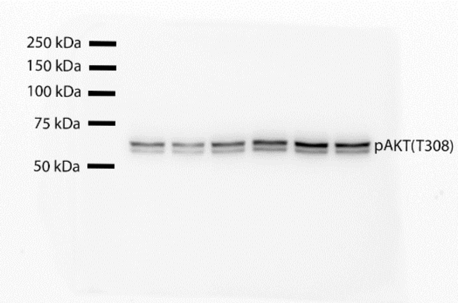

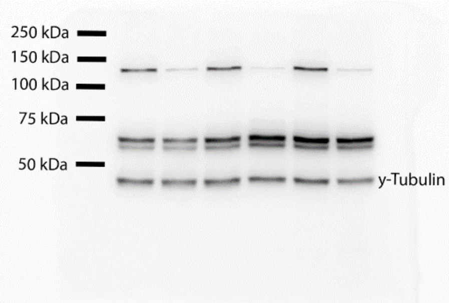

Western blots related to Figure 6 and Supplementary Figure 4: PC-3 samples

Lanes from left to right: siCTRL -, siDHRS7 -, siCTRL EGF 1min, siDHRS7 EGF 1min, siCTRL EGF 5min, siDHRS7 EGF 5min.

DHRS7 and γ-Tubulin #1

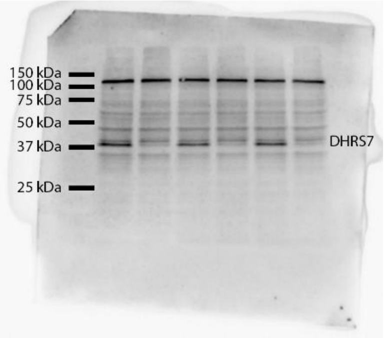

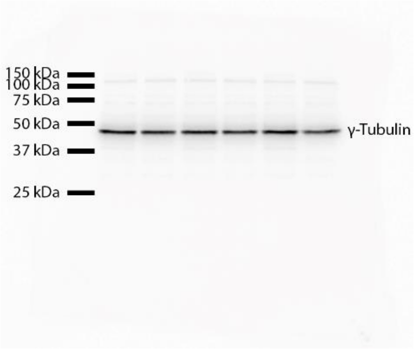

DHRS7 and γ-Tubulin #2

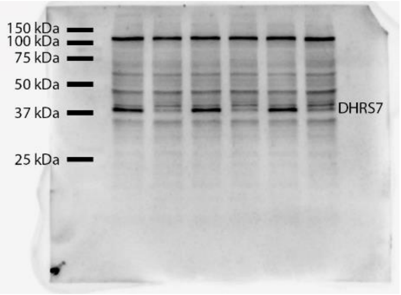

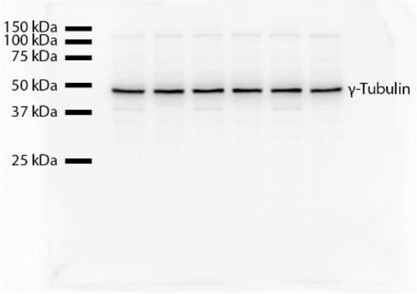

DHRS7 and γ-Tubulin #3

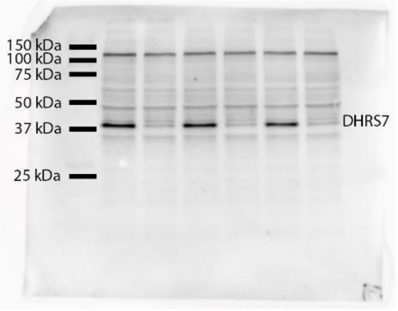

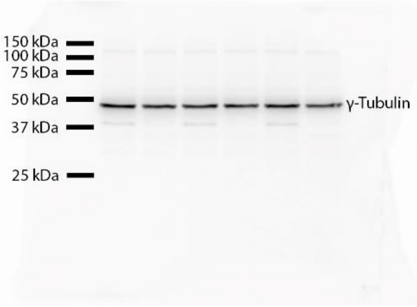

EGFR, γ-Tubulin, ERK and AKT #1

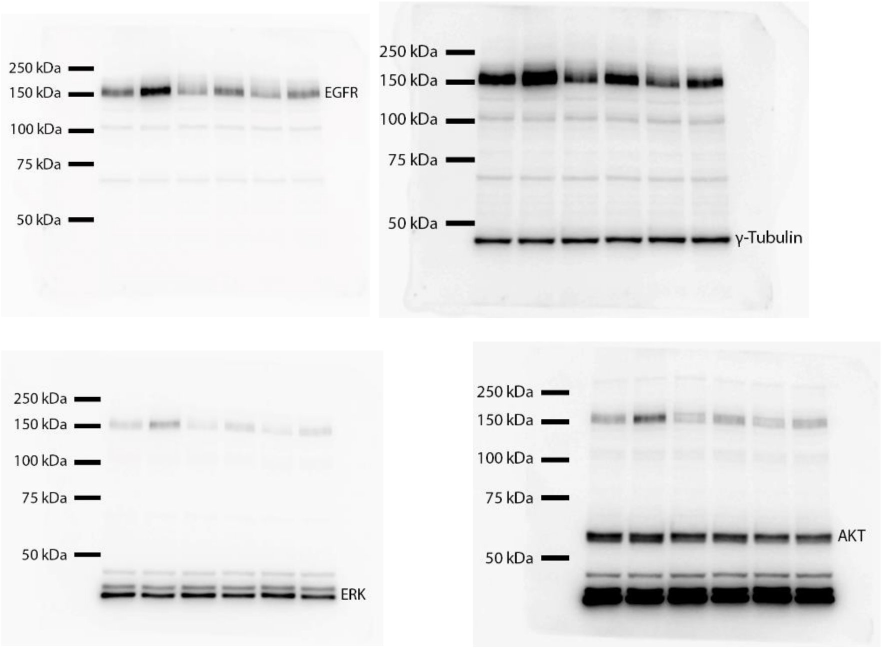

EGFR, γ-Tubulin, ERK and AKT #2

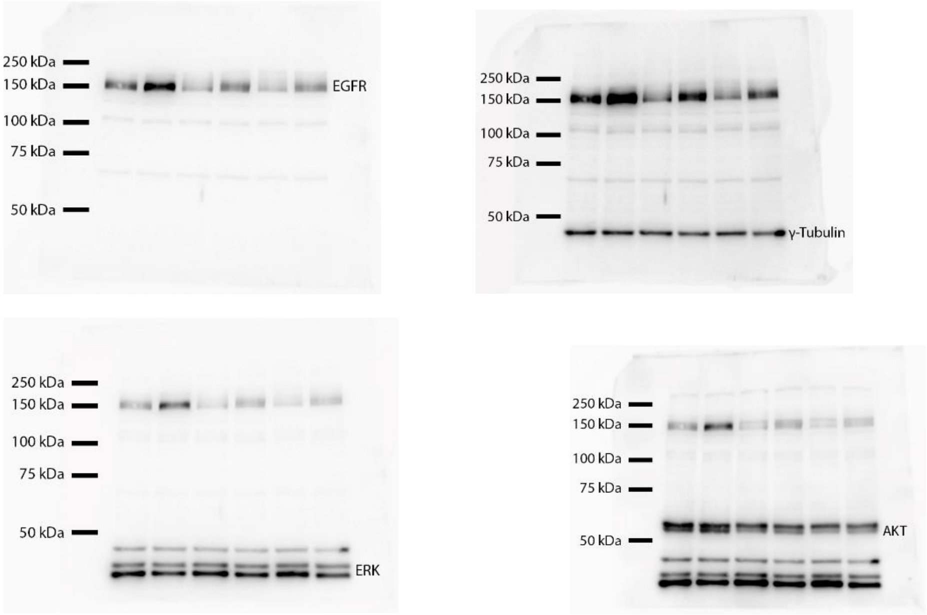

EGFR, γ-Tubulin, ERK and AKT #3

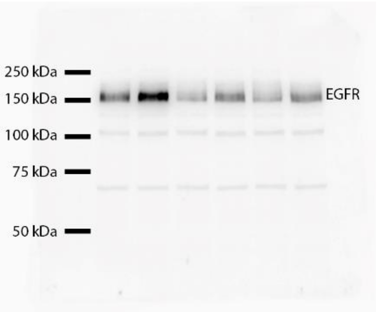

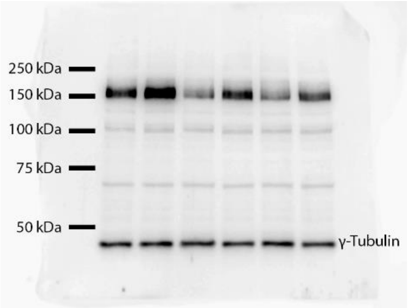

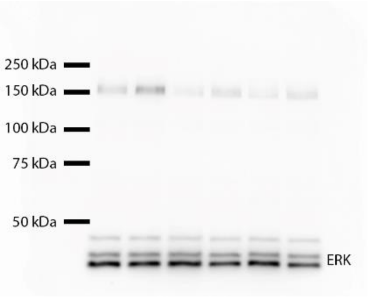

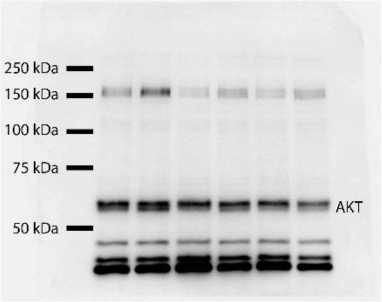

pEGFR, γ-Tubulin, pERK and pAKT(S473) #1

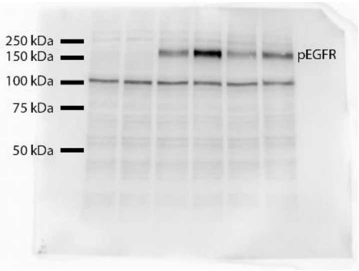

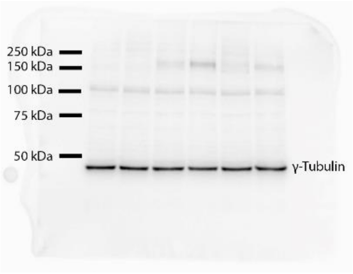

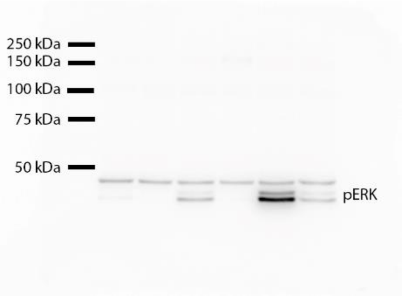

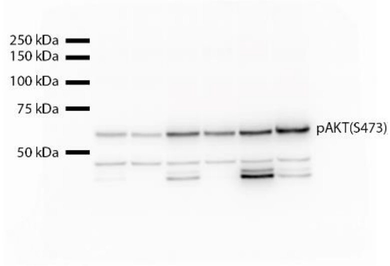

pAKT(T308) and y-Tubulin #1

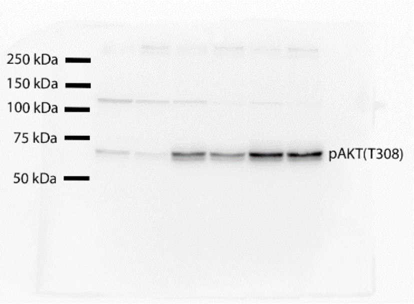

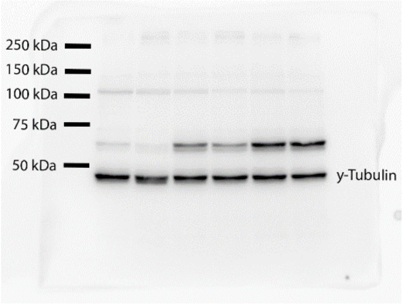

pEGFR, γ-Tubulin, pERK and pAKT(S473) #2

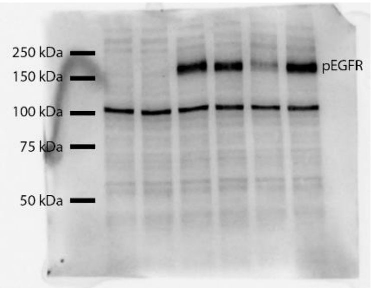

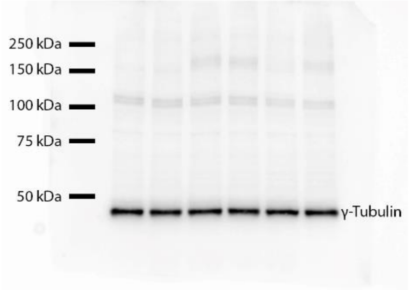

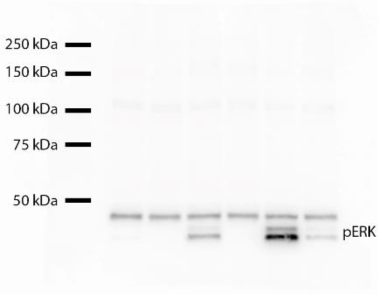

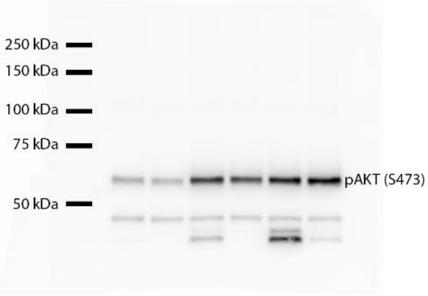

pAKT(T308) and y-Tubulin #2

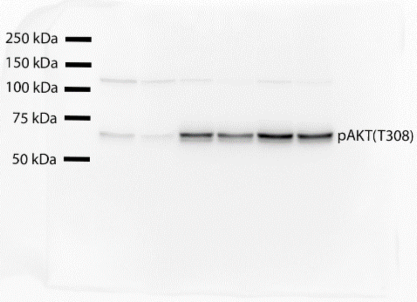

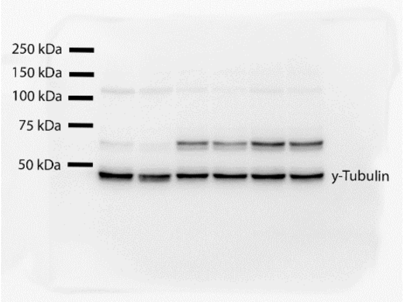

pEGFR, γ-Tubulin, pERK and pAKT(S473) #3

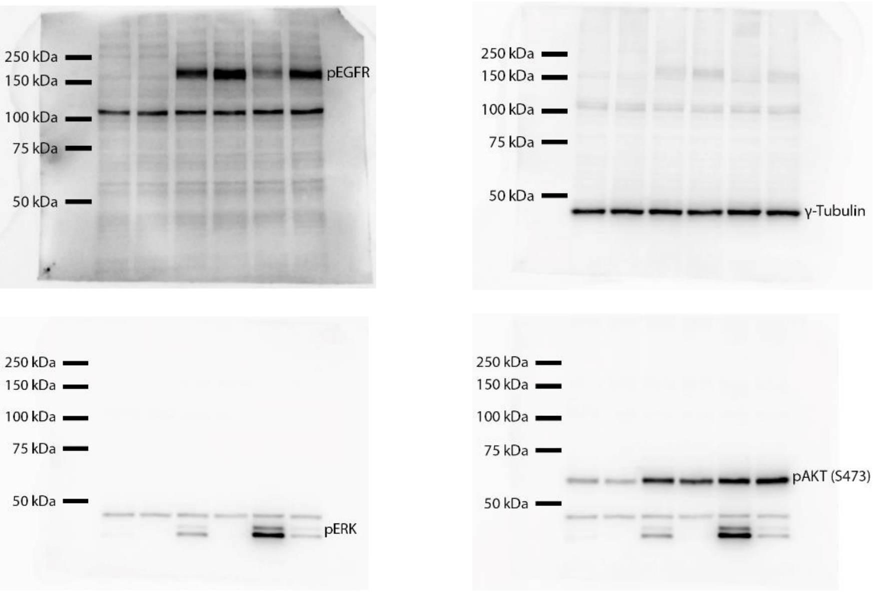

pAKT(T308) and γ-Tubulin #3

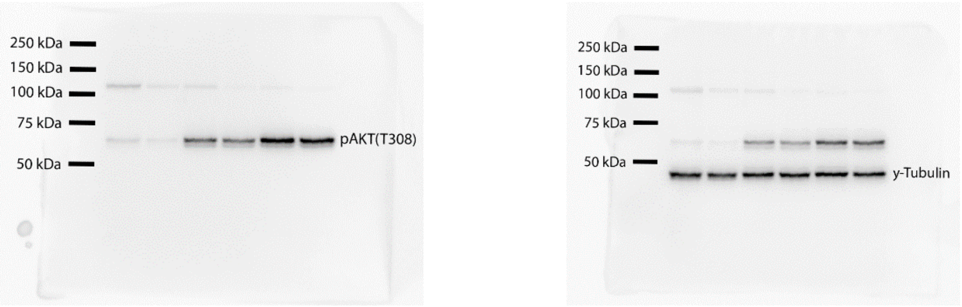

<u>Western blots related to Figure 6 and Supplementary Figure 4: DU145 samples</u>

Lanes from left to right: siCTRL -, siDHRS7 -, siCTRL EGF 1min, siDHRS7 EGF 1min, siCTRL EGF 5min, siDHRS7 EGF 5min.

DHRS7 and γ-Tubulin #1

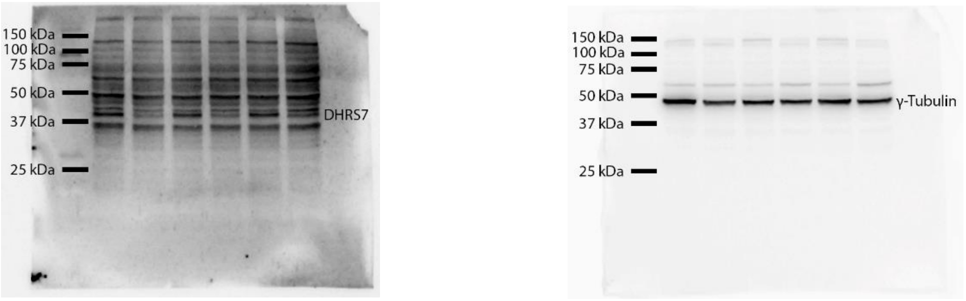

DHRS7 and γ-Tubulin #2

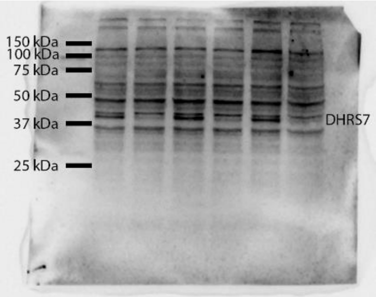

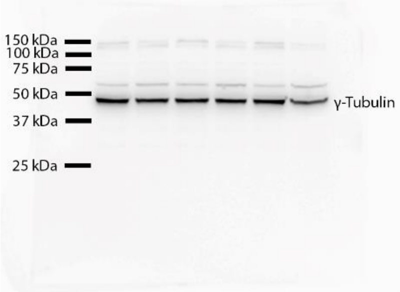

DHRS7 and γ-Tubulin #3

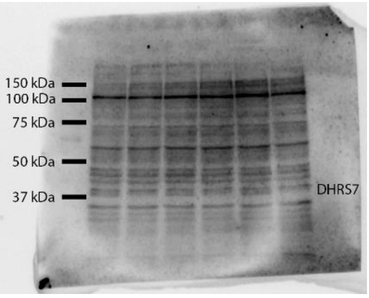

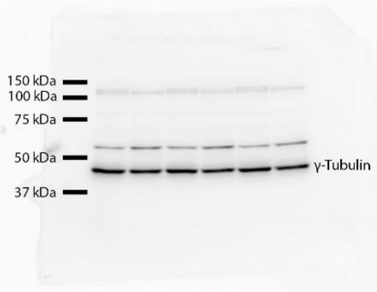

EGFR, STAT3, γ-Tubulin, ERK and AKT #1

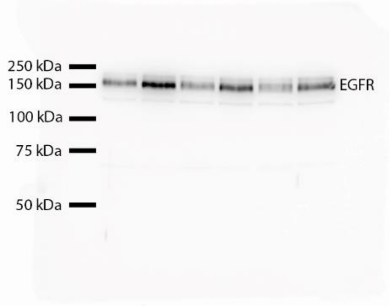

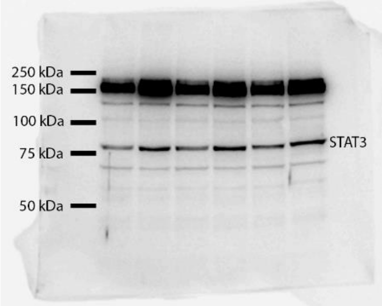

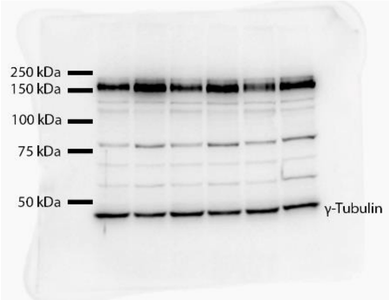

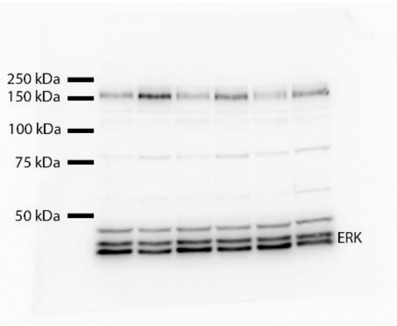

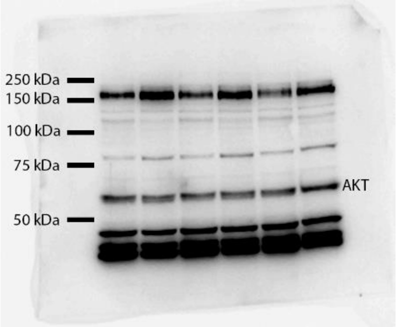

EGFR, STAT3, γ-Tubulin, ERK and AKT #2

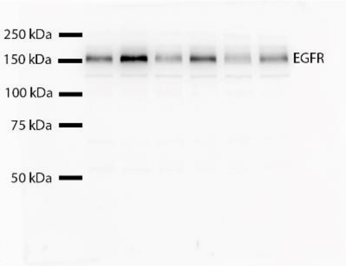

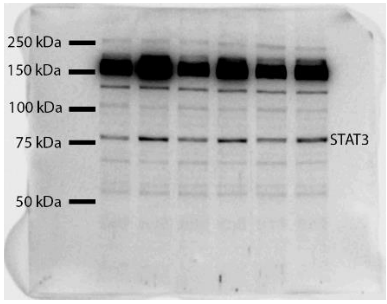

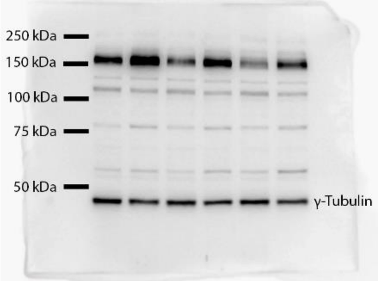

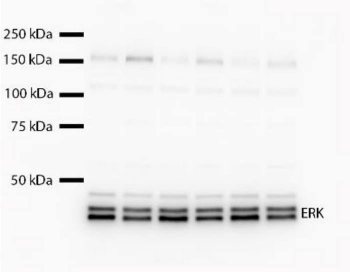

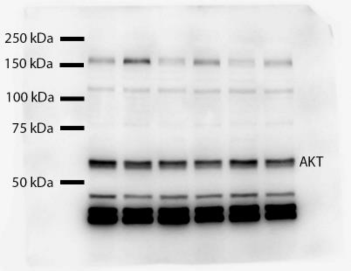

EGFR, STAT3, γ-Tubulin, ERK and AKT #3

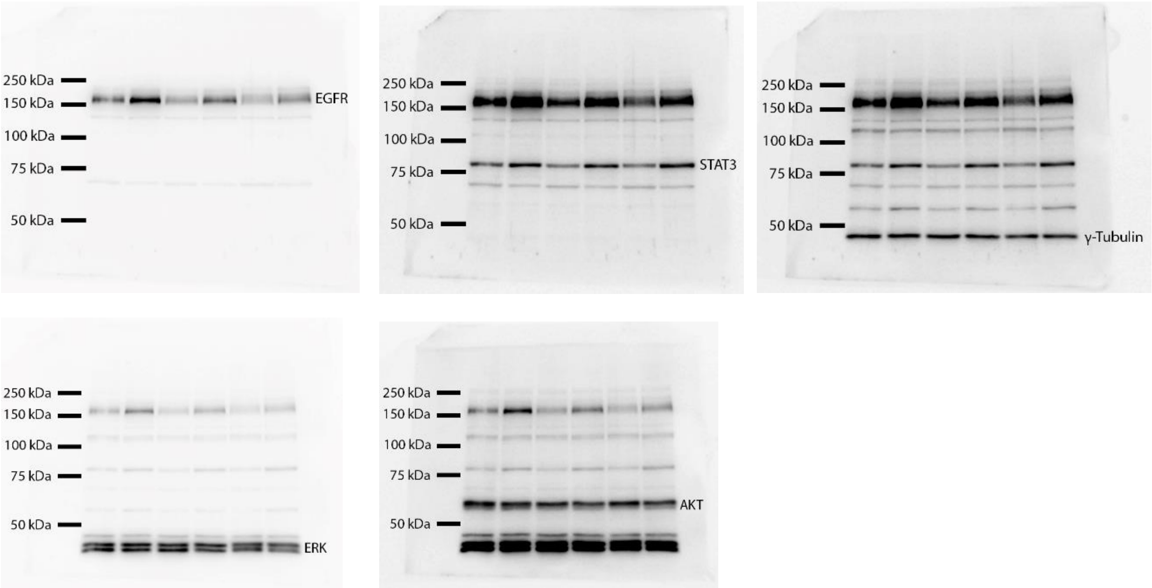

pEGFR, pSTAT3, γ-Tubulin, pERK and pAKT(S473) #1

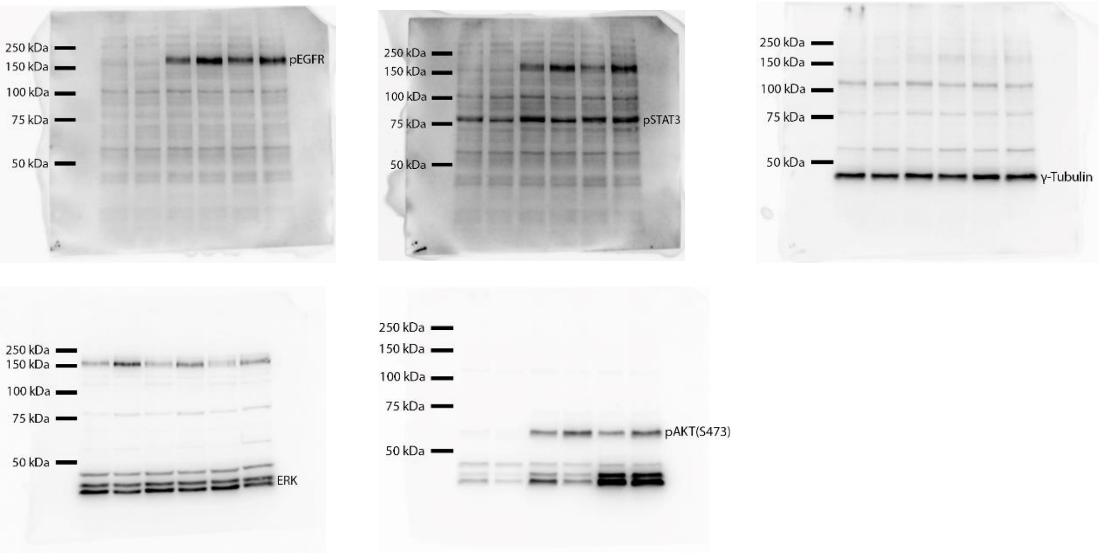

pAKT(T308) and y-Tubulin #1

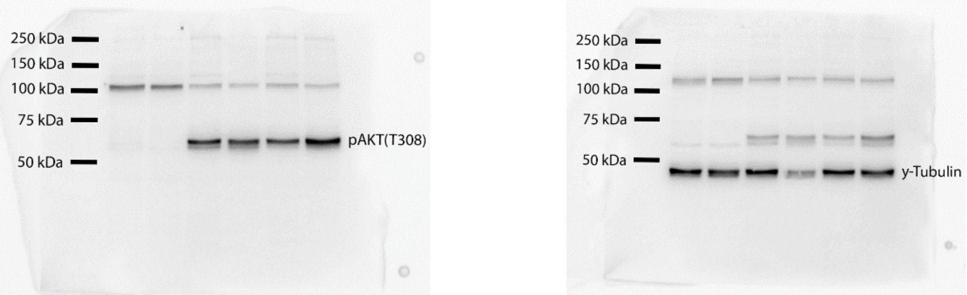

pEGFR, pSTAT3, γ-Tubulin, pERK and pAKT(S473) #2

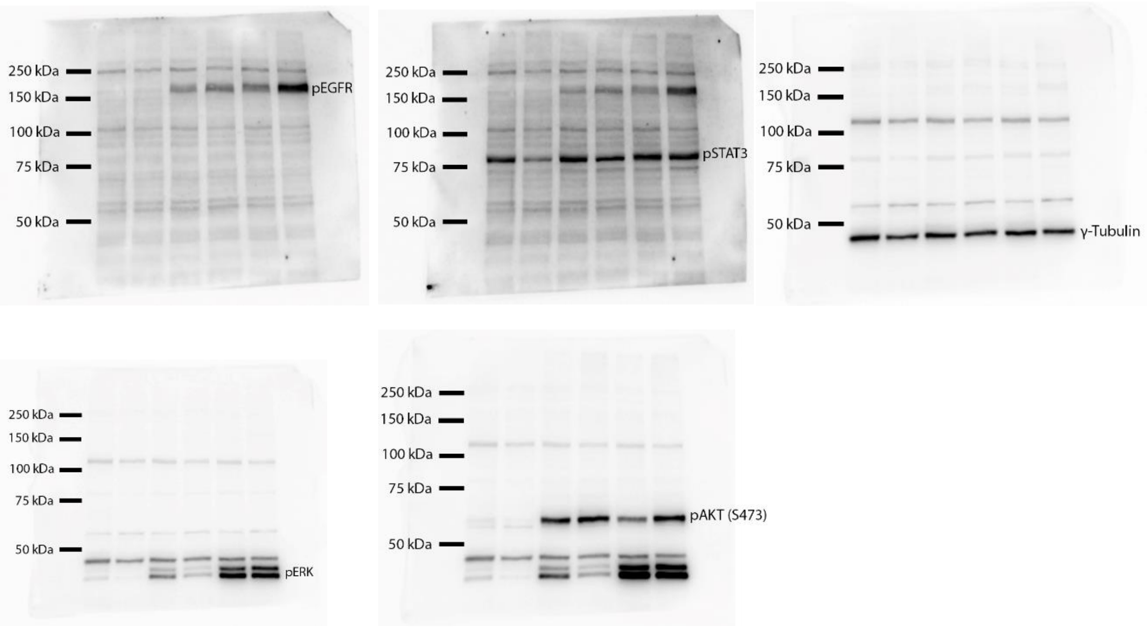

pAKT(T308) and y-Tubulin #2

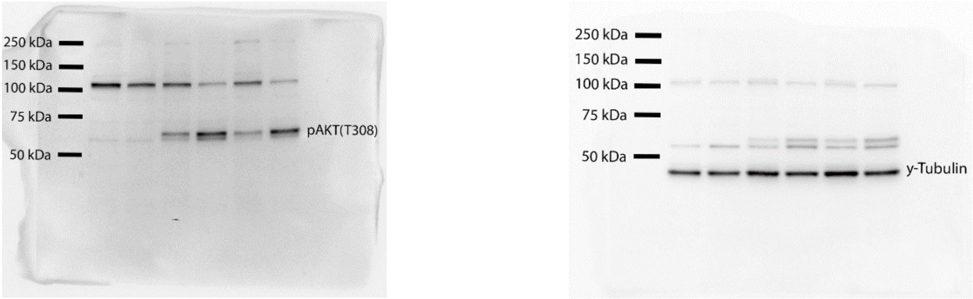

pEGFR, pSTAT3, γ-Tubulin, pERK and pAKT(S473) #3

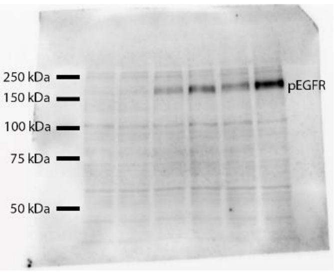

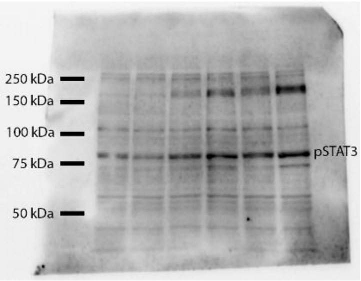

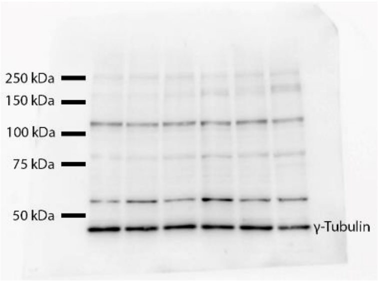

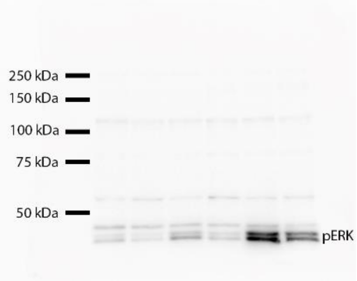

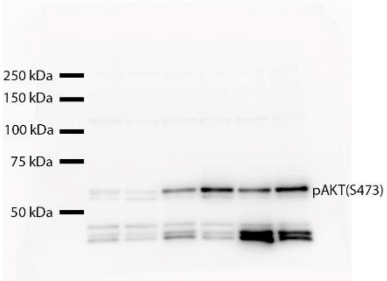

pAKT(T308) and γ-Tubulin #3

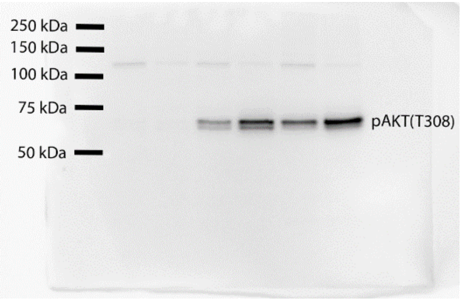

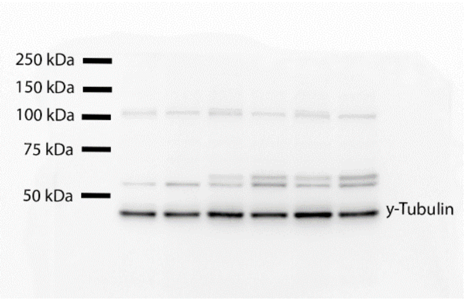

<u>Western blots related to Supplementary Figure 2</u>

Lanes from left to right: siCTRL 24h, siDHRS7 24h, siCTRL 36h, siDHRS7 36h, siCTRL 48h, siDHRS7 48h.

AR and γ-Tubulin #1

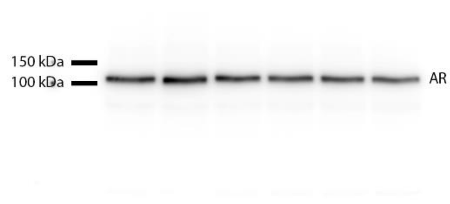

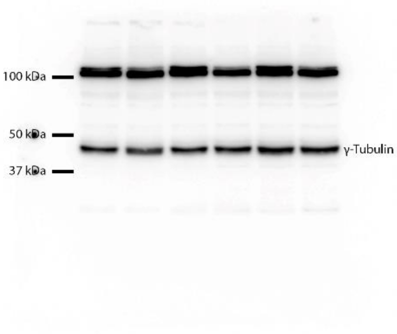

AR and γ-Tubulin #2

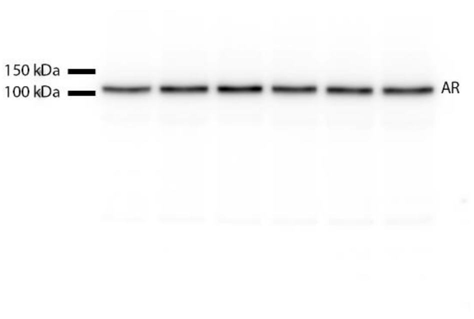

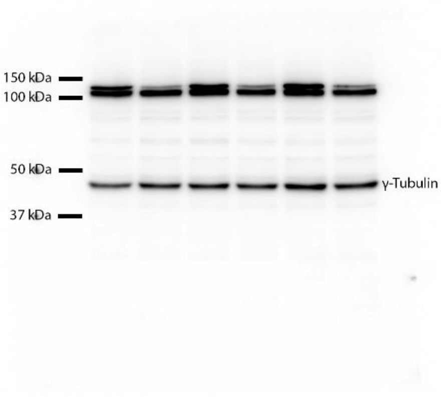

AR and γ-Tubulin #3

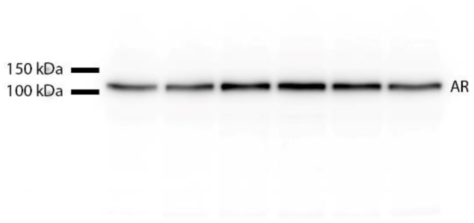

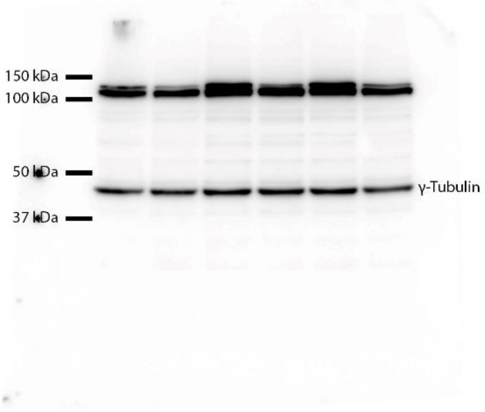

TMPRSS2, FKBP5 and γ-Tubulin #1

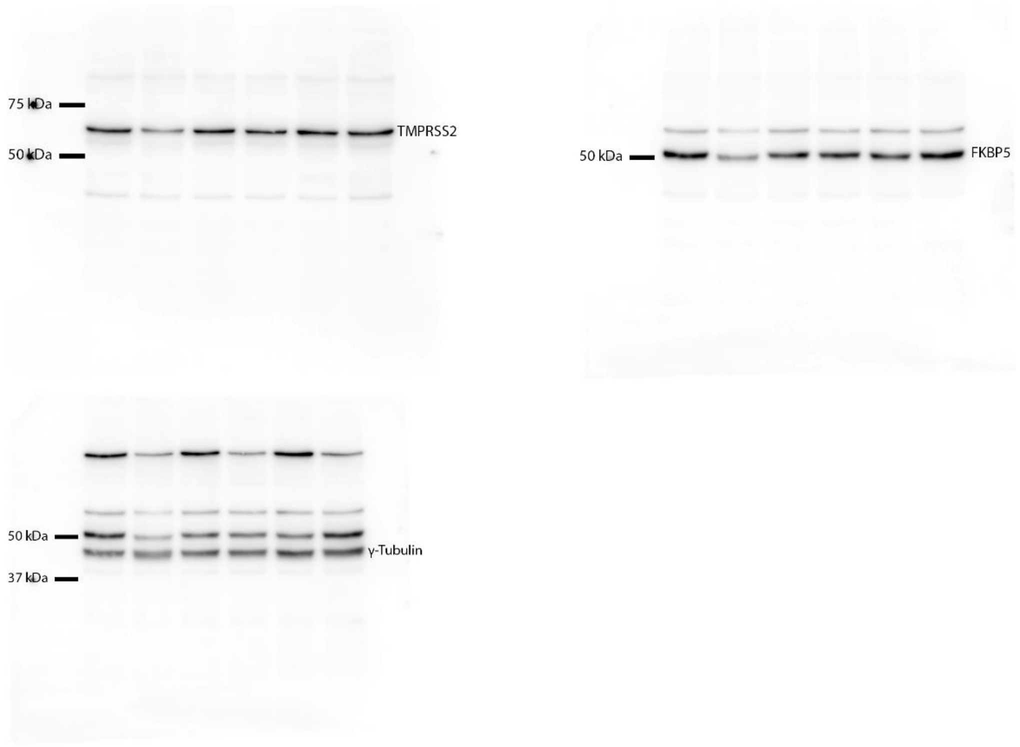

TMPRSS2, FKBP5 and γ-Tubulin #2

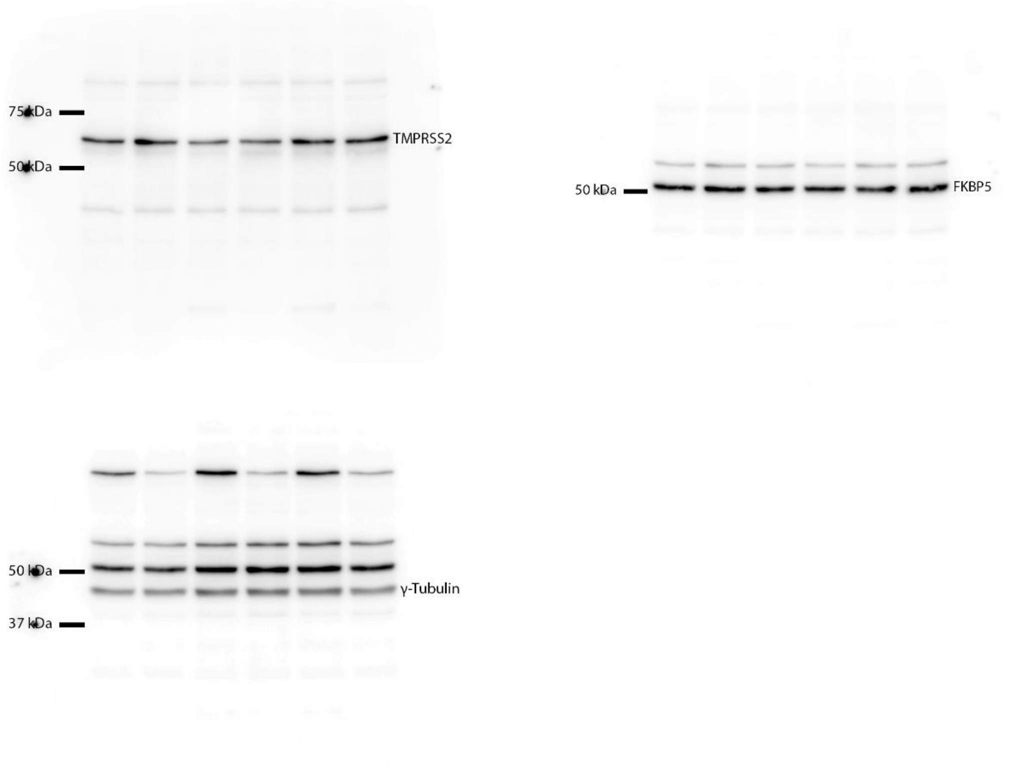

TMPRSS2, FKBP5 and γ-Tubulin #3

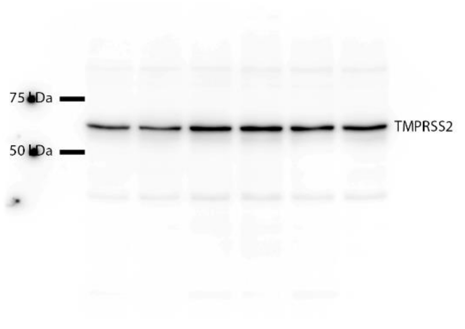

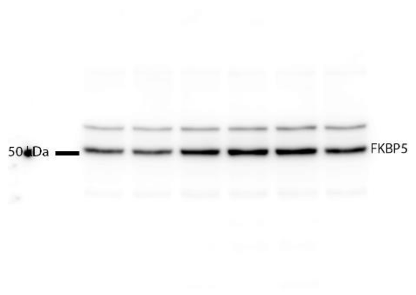

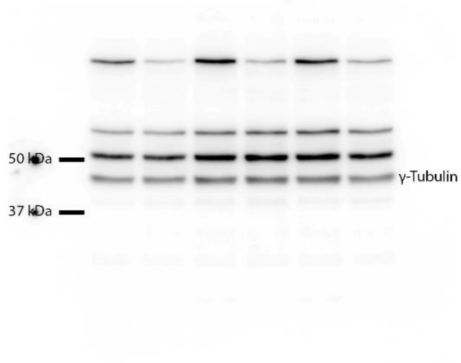

KLK3 and γ-Tubulin #1

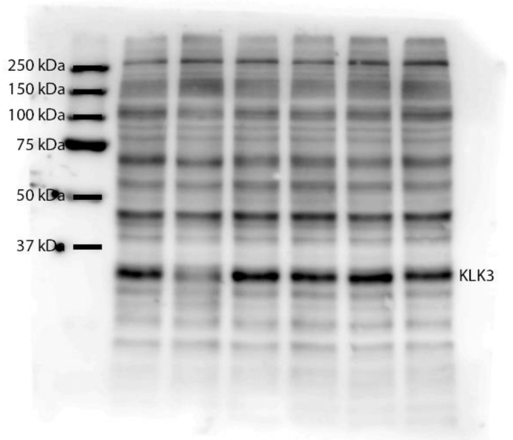

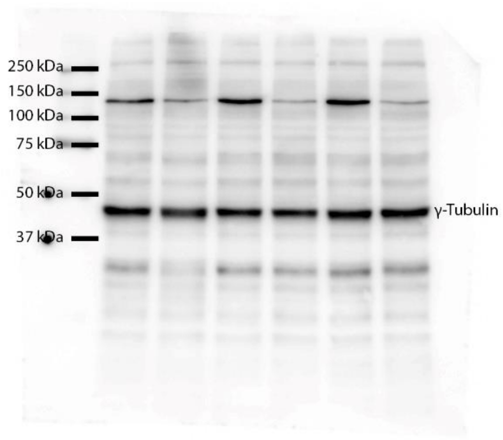

KLK3 and γ-Tubulin #2

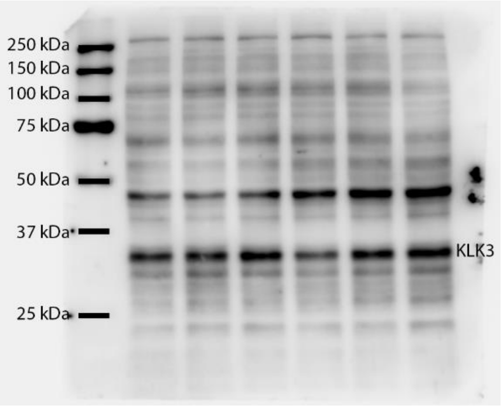

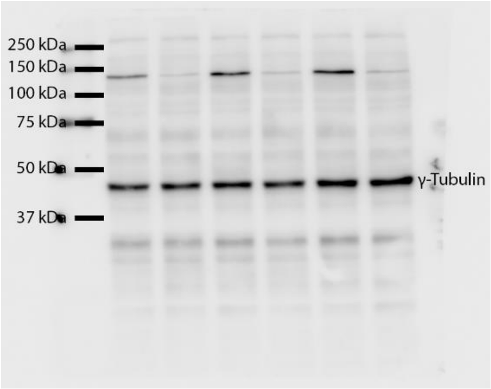

KLK3 and γ-Tubulin #3

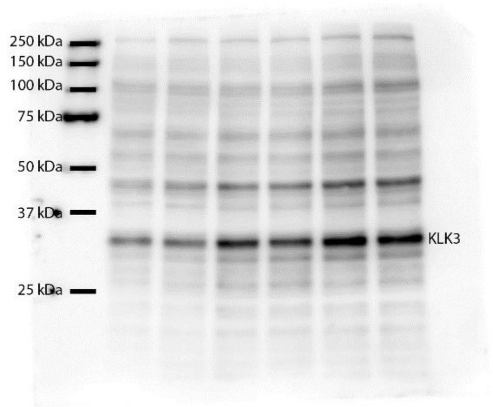

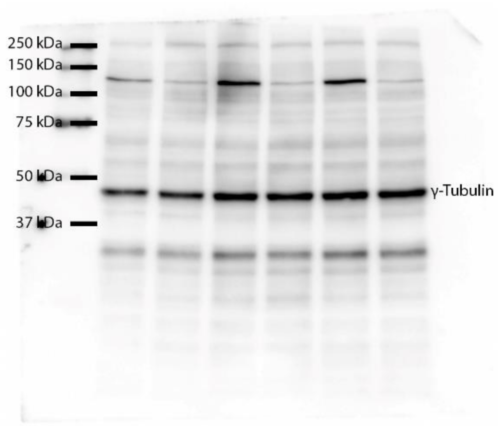

Supplement: Supplementary file 1 [file cancers-14-03074-s001.zip › DHRS7_Supplementary_File5.pdf]
