# Supplementary figures and images for: The Potential Tumor-Suppressor DHRS7 Inversely Correlates with EGFR Expression in Prostate Cancer Cells and Tumor Samples
Source: Cancers (Basel). 2022 Jun 23;14(13):3074. doi: 10.3390/cancers14133074 (PMC9264982; doi:10.3390/cancers14133074)

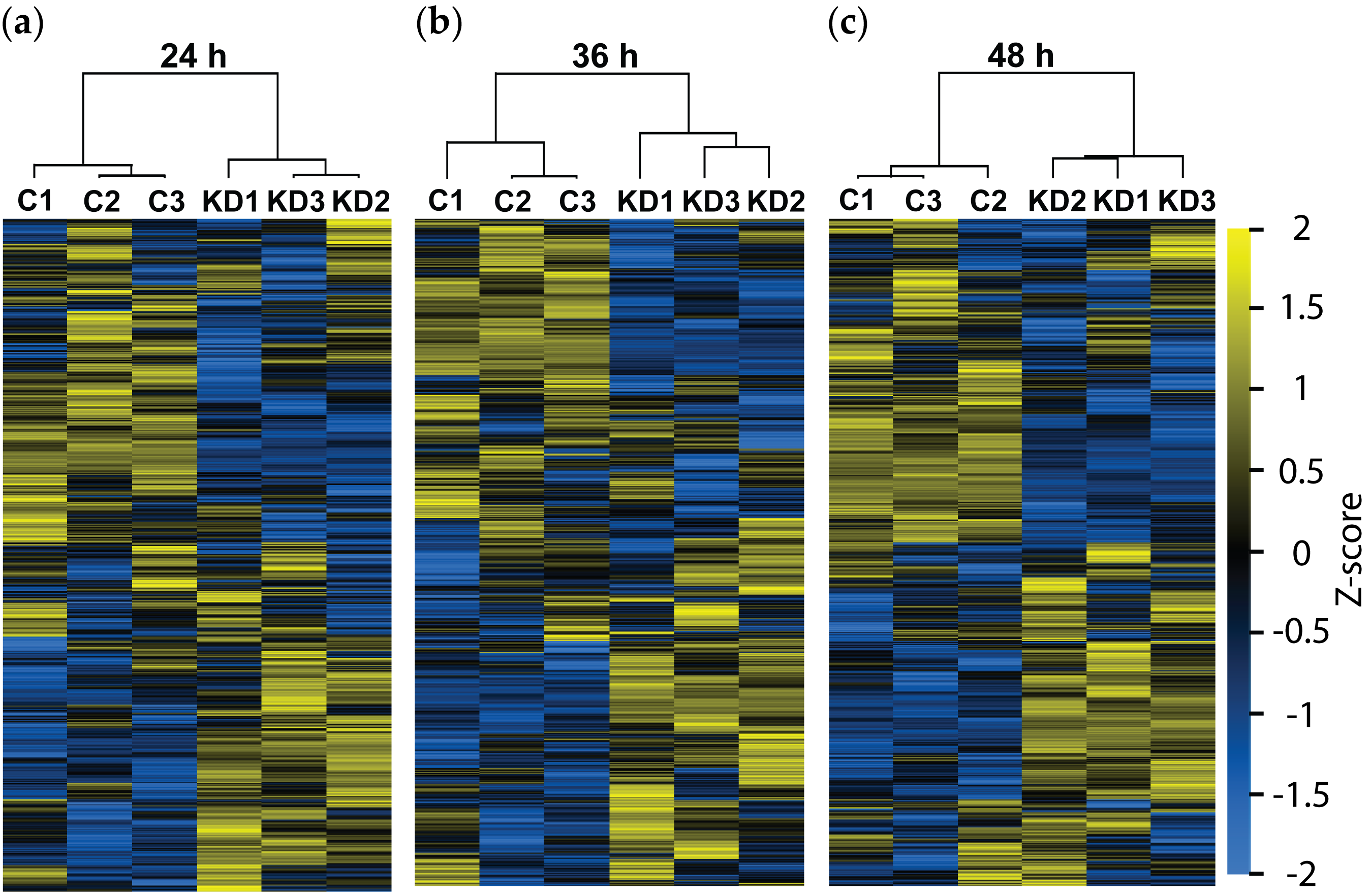

Supplement: Supplementary file 1 [file cancers-14-03074-s001.zip › DHRS7_FigureS1.tif]

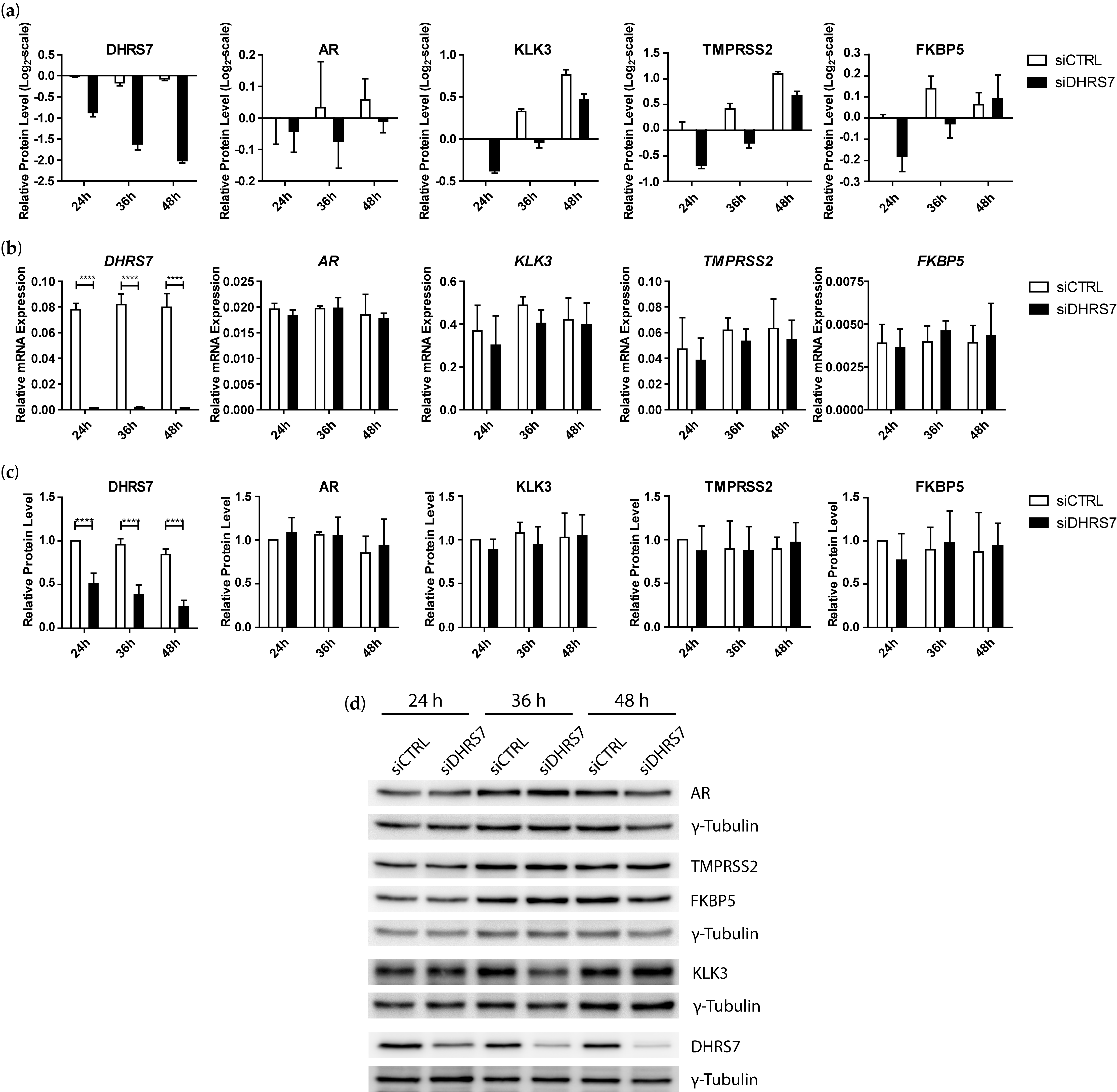

Supplement: Supplementary file 1 [file cancers-14-03074-s001.zip › DHRS7_FigureS2.tif]

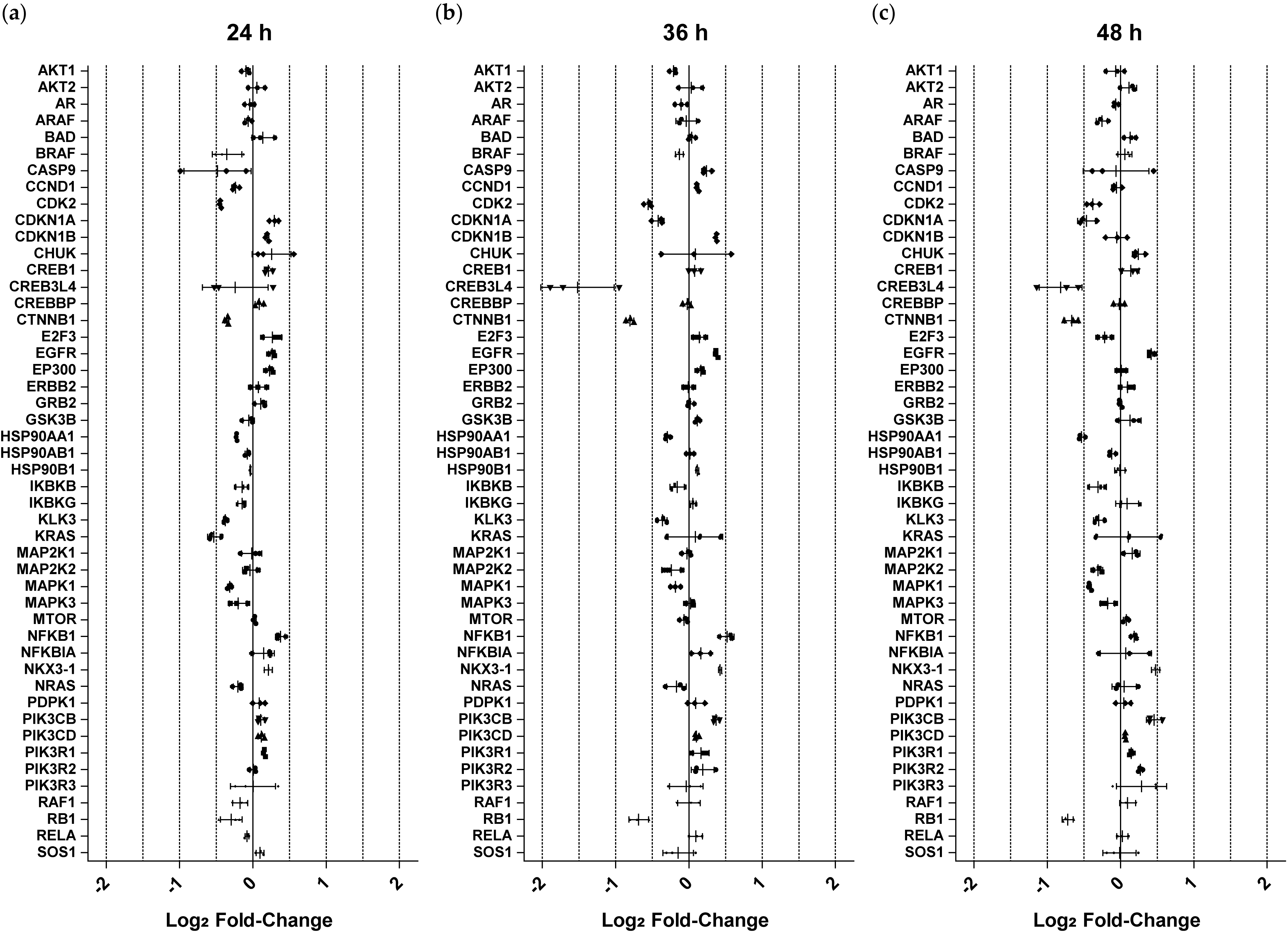

Supplement: Supplementary file 1 [file cancers-14-03074-s001.zip › DHRS7_FigureS3.tif]

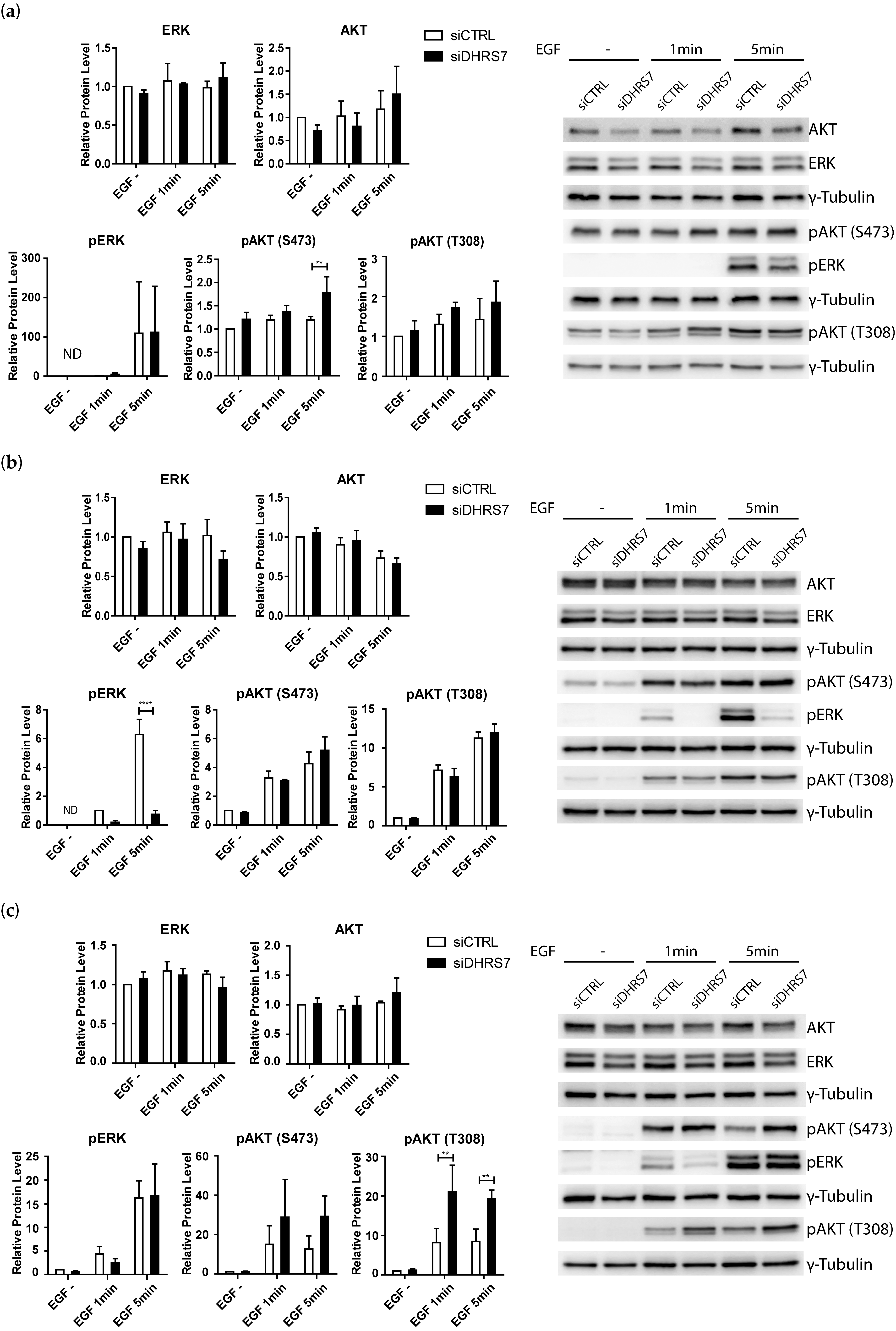

Supplement: Supplementary file 1 [file cancers-14-03074-s001.zip › DHRS7_FigureS4.tif]

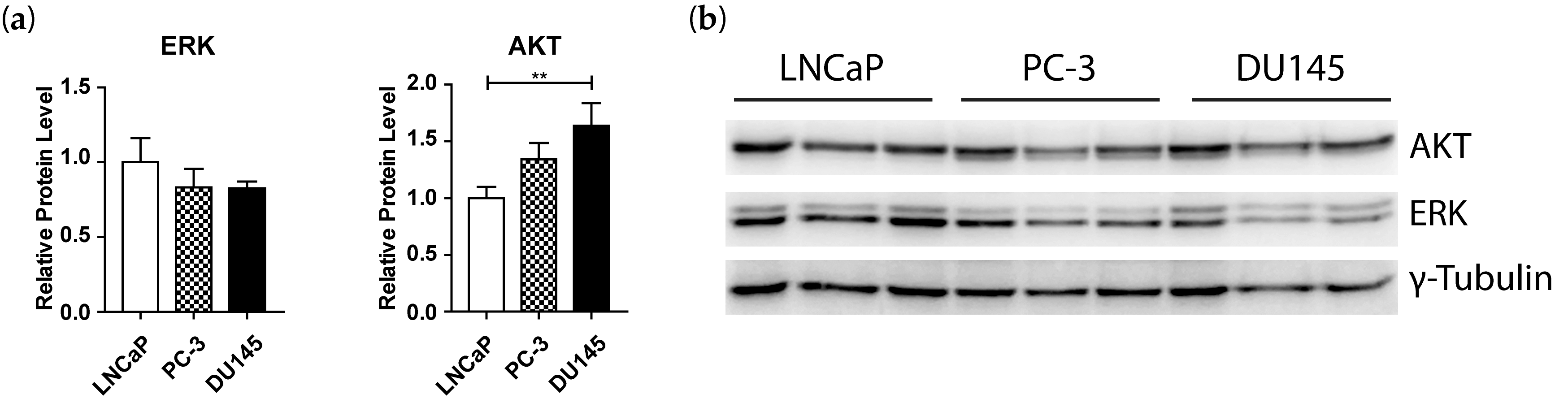

Supplement: Supplementary file 1 [file cancers-14-03074-s001.zip › DHRS7_FigureS5.tif]

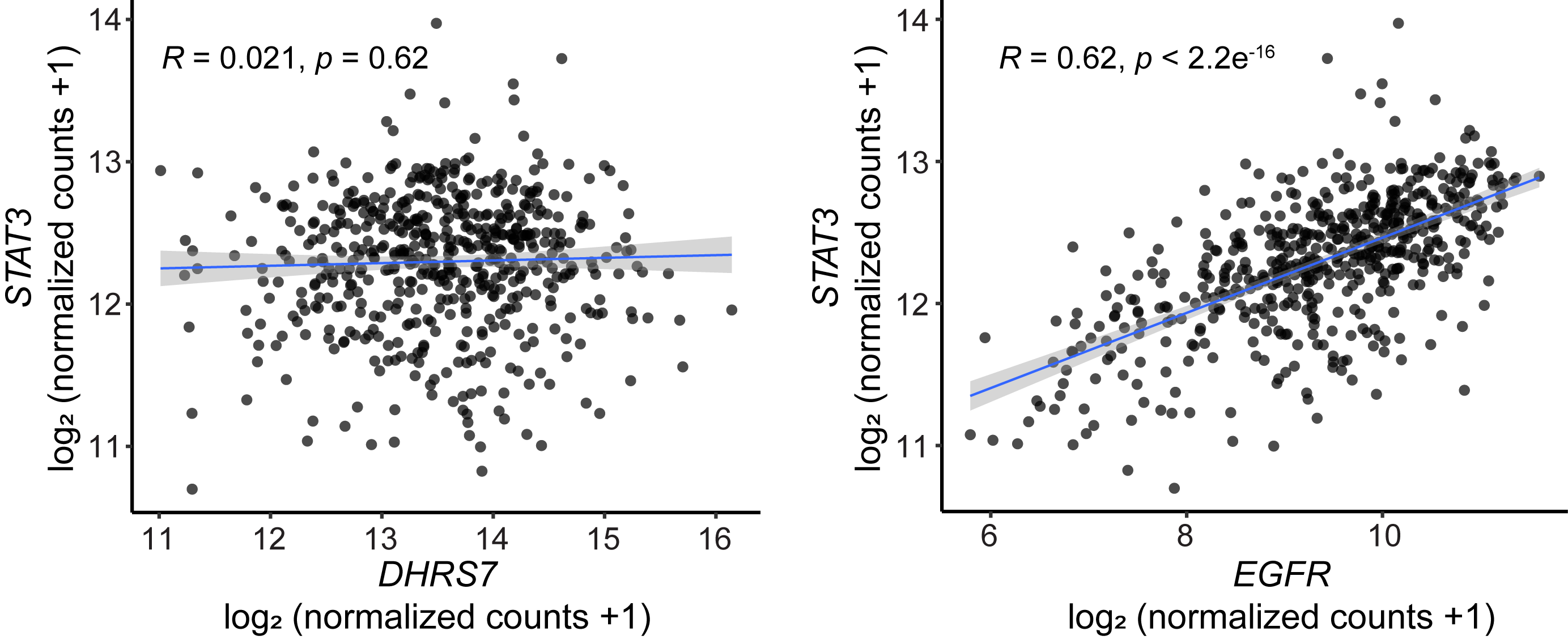

Supplement: Supplementary file 1 [file cancers-14-03074-s001.zip › DHRS7_FigureS6.tif]

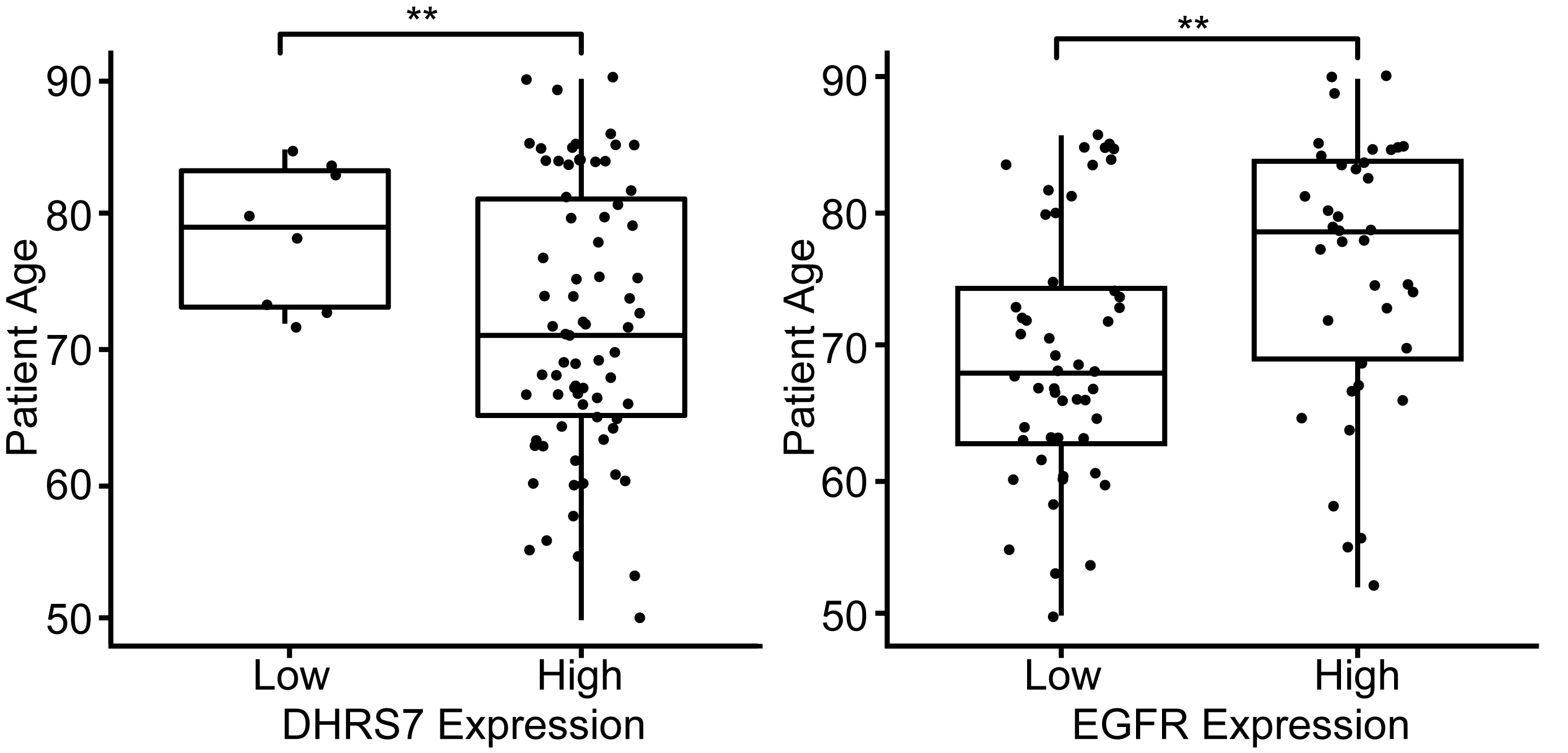

Supplement: Supplementary file 1 [file cancers-14-03074-s001.zip › DHRS7_FigureS7.tif]
